# Photoredox-catalyzed δ-aminomethylation of trifluoroacetamides with oximes as radical traps

**DOI:** 10.1039/d6ra04812a

**Published:** 2026-07-17

**Authors:** María Valerio Roa, Melissa A. Ashley, Katherine A. Xie, Tomislav Rovis

**Affiliations:** a Department of Chemistry, Columbia University New York NY 10027 USA tr2504@columbia.edu

## Abstract

Methods to achieve C–H functionalization constitute powerful tools in synthetic chemistry. Due to the ubiquity of amines in pharmaceuticals and natural products, selective amine functionalization is of great interest. However, site-selective functionalization of primary aliphatic amines poses a significant challenge, as the α-position to the amine is electronically biased and the least hindered terminal position is the most accessible. We and others have recently addressed this challenge by harnessing 1,5-hydrogen atom transfer (HAT) from an amidyl radical to selectively activate one position along the aliphatic chain for Giese couplings and Ni-catalyzed alkylations. Herein, we describe the use of Ir(iii) photoredox catalysis to selectively couple oximes to the δ-position of primary aliphatic amine-derived trifluoroacetamides through a HAT process. This method allows for the δ-functionalization of 2° and 3° trifluoroacetamides with good yields and excellent selectivity.

## Abstract 2

Photoredox-catalyzed site-selective δ-aminomethylation of primary amine derivatives *via* 1,5-hydrogen atom transfer.

## Graphical abstract

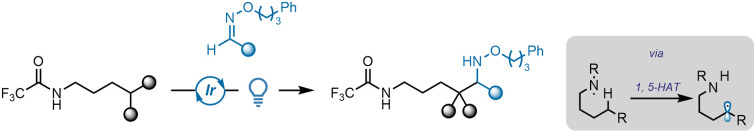

Due to their ubiquity in pharmaceuticals and natural products, amines constitute one of the most valuable synthetic building blocks.^1^ Therefore, functionalization of primary aliphatic amine chains is of significant interest. However, achieving site-selective functionalization of C(sp^3^)–H bonds remains a major challenge in synthetic chemistry due to their enthalpic stability (∼100 kcal mol^−1^).^2^ In the case of primary amines, site-selective functionalization is particularly challenging, as the α position of the amine is electronically biased, while the least hindered terminal position is the most accessible. Furthermore, most C(sp^3^)–H bonds in an aliphatic amine chain are largely indistinguishable on the basis of their bond dissociation energies.

Hydrogen atom transfer (HAT) methods have proven effective for site-selectively functionalizing otherwise unreactive C(sp^3^)–H bonds.^3^ These proceed *via* radical intermediates generated under mild conditions using visible-light photoredox catalysis.^4^ Notably, these approaches have been successfully applied to the site-selective functionalization of primary amine derivatives.^5–7^

In particular, targeting the δ-selective functionalization of primary amine derivatives, in 2016, our group and the Knowles group reported the formation of nitrogen-centered radicals by single electron oxidation of trifluoroacetamides and benzamides, respectively (Scheme 1). The reaction is proposed to proceed *via* an Ir(iii)* single electron oxidation of the amidyl anion, which then undergoes 1,5-HAT, generating a new carbon-centered radical that is site-selectively trapped with Michael acceptors.^8,9^ Similarly, in 2019, we reported a dual photoredox and nickel catalyzed system, in which a Ni(ii) catalyst is proposed to trap the carbon-centered radical, alkylating the δ-position of trifluoroacetamides using alkyl bromides (Scheme 1).^10^ Given the prevalence of nitrogen in various small molecules and pharmaceuticals, we sought to use aminating reagents that can function as radical traps, to site-selectively introduce an amine functionality at the δ-site. To this end, we hypothesized oximes would be best suited to trap the nucleophilic carbon-centered radicals. While oximes have been extensively used as coupling partners in transition metal-catalyzed reactions (particularly for C(sp^2^)–C(sp^3^) bond formation) and radical cascade reactions,^11^ they have been underexplored radical traps using photoredox catalysis in particular for C(sp^3^)–C(sp^3^) bond formation.^12^

**Scheme 1:**
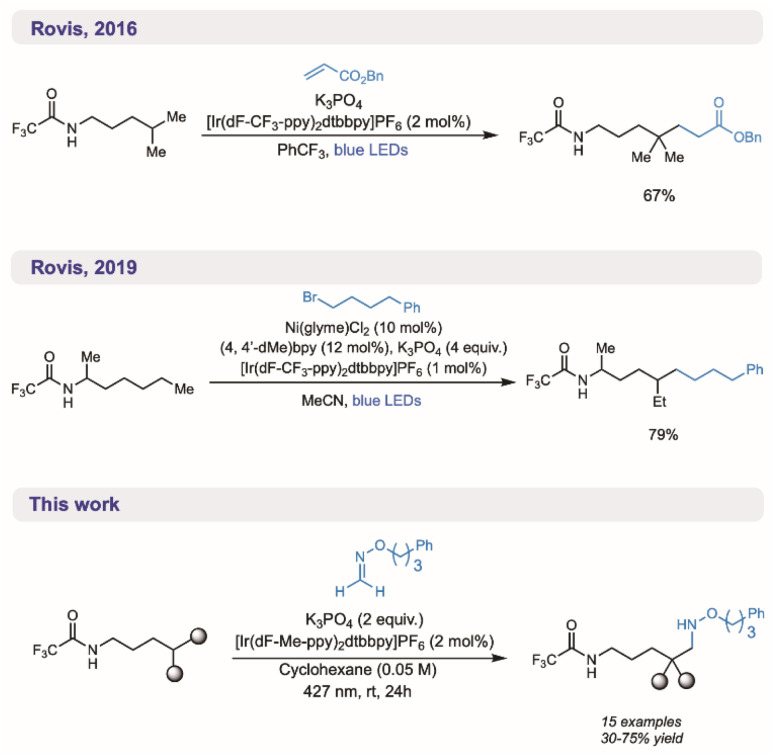
Precedent for the photoredox-enabled δ-selective functionalization (through HAT strategies) of primary amine derivatives and comparison to this current work.

Our initial hit was found using a formaldehyde derived benzyl oxime for the site-selective δ-aminomethylation of trifluoroacetamides, which preferentially engage in 1,5-HAT under a sufficiently strong phosphate base^8,10^ (Table 1). A solvent screen was executed using (Ir[dF(CF_3_)ppy]_2_(dtbpy))PF_6_ (*E*_1/2_(Ir^III/II^) = −1.37 V, 
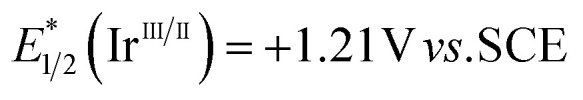
) at 3 mol% catalyst loading and K_3_PO_4_ as the base. Results from a solvent screen using benzyl oxime as a radical trap indicate unaccounted-for mass balance, particularly in trifluorotoluene when compared with cyclohexane. To probe this finding mechanistically, we monitored the reaction by NMR, which revealed product degradation and benzaldehyde formation. At this stage, it is not clear whether the oxyaminyl radical that is generated as a trap undergoes beta-scission to form the stable benzyl radical, which may subsequently oxidize, or whether the activated benzyl ether position undergoes competitive intermolecular HAT which is then followed by oxidation. In either case, we deemed it expedient to use a different substituent on the oxime.

**Table 1:** Optimization, control, and robustness studies^b^

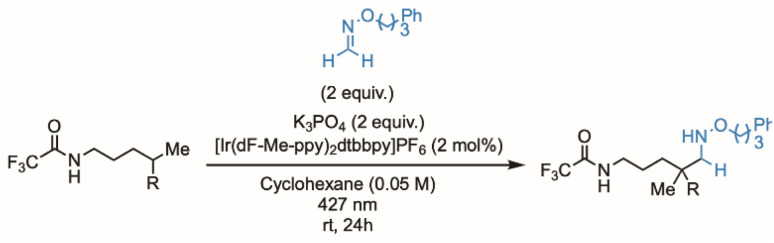			
Entry	R Group	Deviation	Yield (%)
1	H	None	67
2	Me	None	82
3	H	Trifluorotoluene	40^a^
4	H	DMSO	26^a^
5	H	DCE	24^a^
6	Me	[Ir(dF-CF_3_-ppy)_2_dtbbpy]PF_6_	56
7	H	No photocatalyst	0
8	H	No light	0
9	H	No base	0
10	Me	Base weighed under air	0

a NMR yield with 0.1 mmol of mesitylene as internal standard.

b All optimizations were run at 0.1 mmol scale using 1 equiv. trifluoroacetamide, 2 equiv. *O*–Phenylpropyl oxime, 2 equiv. base, and photocatalyst (2 mol%) over a period of 24 hours.

To deter this undesired pathway, we synthesized an oxime coupling partner which minimizes the presence of activated C–H bonds *via* extension of the alkyl chain, which increases the bond strength of the α-oxy C–H bonds. Using formaldehyde *O*–(3-phenylpropyl) as our model oxime under the aforementioned reaction conditions with cyclohexane as a solvent, we obtain our desired product in 56% yield, with no aldehyde byproduct. When utilizing the less oxidizing photocatalyst [Ir(dF(Me)ppy)_2_(dtbbpy)]PF_6_ (*E*_1/2_(Ir^III/II^) = −1.41 V, 
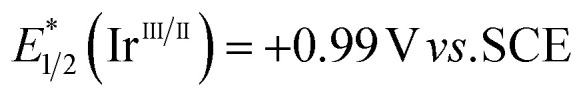
) at 2 mol% catalyst loading, the reaction delivers the desired product in 82% yield. Moreover, control experiments show that the reaction does not proceed without light, catalyst, base, or if the base is added under aerobic conditions.

With optimized conditions in hand, we explored the scope for primary amine derivatives (Scheme 3). We found that the method worked particularly well for tertiary δ-sites (1 to 8) with a decrease in yield observed with secondary (9 to 11) and primary δ-centers, only obtaining trace yields for the latter. This trend is consistent with the relative stabilities of the δ-centered radical following 1,5-HAT. Alkyl substituents in the position α of the amine have no particular effect on the yield of the δ-functionalized product (1, 2, and 5). In substrates with electronically biased C(sp^3^)–H bonds, such as those containing heteroatoms (7 and 10), we selectively functionalize the δ-site of the trifluoroacetamides in good yields with respect to the degree of substitution of the δ-center. This allows for the functionalization of ethers (7, 8, and 10). Cyclic systems (6) and (11) are also well-tolerated. A six-membered ring system with a secondary δ-center (11) was functionalized with 2 : 1 diastereoselectivity, with preference for the *trans* diastereomer.

The oxime scope was also explored. Electrophilic electron-deficient oximes are tolerated under the reaction conditions (12 to 14), albeit with decreased yield. Electron-deficient oximes that were not sufficiently electrophilic were not trapped by the δ-centered radical (15).

In order to explain the observed regioselectivity, we propose the following mechanism (Scheme 2). The trifluoroacetamide is deprotonated by the phosphate base and then is oxidized by the Ir(iii)* excited state, yielding a trifluoroacetamidyl radical. Then, through a 1,5-HAT event, a new carbon-centered radical is generated at the distal δ-position. Said carbon-centered radical undergoes a single-electron addition to the carbon of the oxime, resulting in a nitrogen-centered radical, which is subsequently reduced by the Ir(ii) state and protonated, furnishing the product and regenerating the Ir(iii) catalyst.

**Scheme 2:**
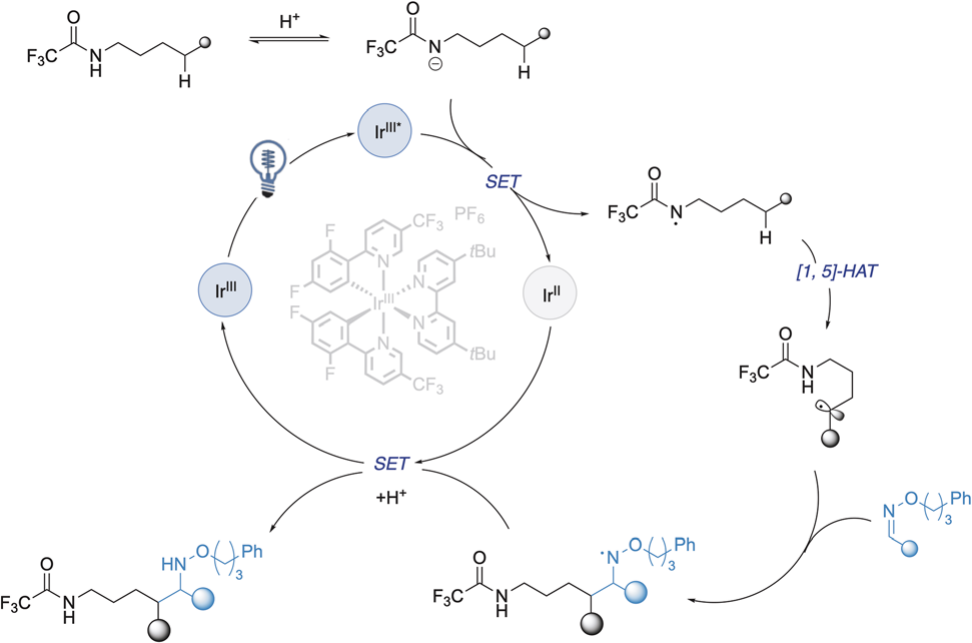
Proposed mechanism for the δ-selective functionalization of trifluoroacetamides using oximes as radical traps.

**Scheme 3:**
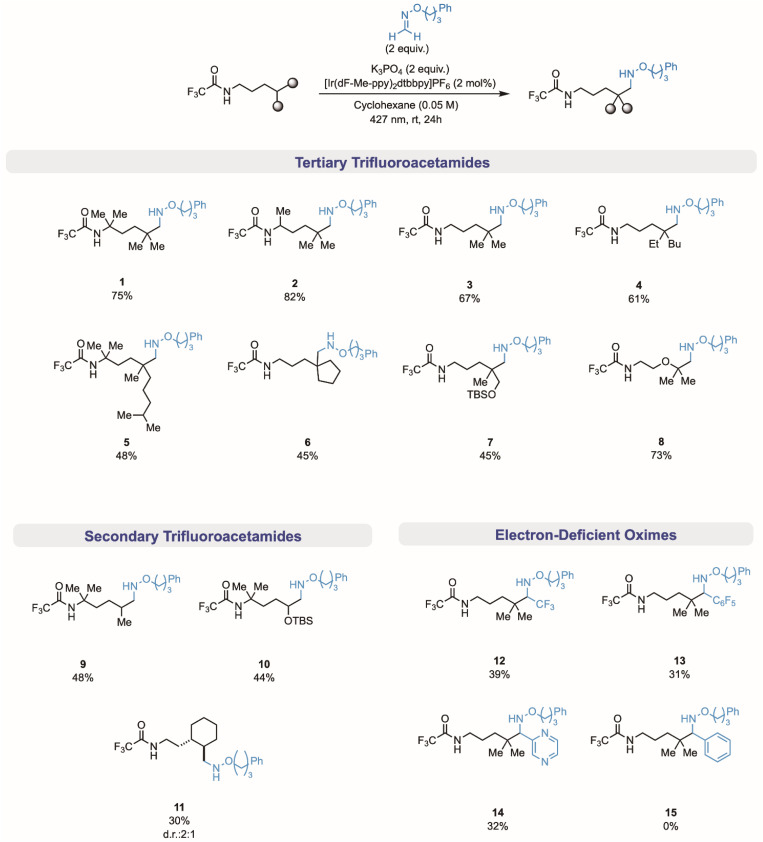
Scope of the reaction.

## Conclusions

Herein we report a method to site-selectively couple oximes to the δ-position of trifluoroacetamides under mild reaction conditions using photoredox catalysis. We evaluate the use of oximes, which introduce molecular complexity to otherwise simpler compounds due to the inclusion of nitrogen and oxygen heteroatoms, as radical traps. Due to their ubiquity in pharmaceuticals and biological products, the C(sp^3^)–H functionalization of amines is of great interest in the synthetic community. Thus, we anticipate this reaction will be of great use in medicinal chemistry.

## Author contributions

MAA and TR conceived the work. Experimental work was conducted by MVR, MAA and KAX. All authors contributed to the writing of the manuscript.

## Conflicts of interest

There are no conflicts to declare.

## Supplementary Material

RA-OLF-D6RA04812A-s001

## Data Availability

The data supporting this article have been included as part of the supplementary information (SI). Supplementary information is available. See DOI: https://doi.org/10.1039/d6ra04812a.

## References

[cit1] Zabolotna Y., Volochnyuk D. M., Ryabukhin S. V., Horvath D., Gavrilenko K. S., Marcou G., Moroz Y. S., Oksiuta O., Varnek A. (2022). J. Chem. Inf. Model..

[cit2] Bergman R. G. (2007). Nature.

[cit3] Capaldo L., Ravelli D. (2017). Eur. J. Org Chem..

[cit4] Prier C. K., Rankic D. A., MacMillan D. W. C. (2013). Chem. Rev..

[cit5] Chen D., Chu J. C. K., Rovis T. (2017). J. Am. Chem. Soc..

[cit6] Ashley M. A., Yamauchi C., Chu J. C. K., Otsuka S., Yorimitsu H., Rovis T. (2019). Angew. Chem., Int. Ed..

[cit7] Morcillo S. P., Dauncey E. M., Kim J. H., Douglas J. J., Sheikh D. S., Leonori D. (2018). Angew. Chem., Int. Ed..

[cit8] Chu J. C. K., Rovis T. (2016). Nature.

[cit9] Choi G. J., Zhu Q., Miller D. C., Gu C. J., Knowles R. R. (2016). Nature.

[cit10] Thullen S. M., Treacy S. M., Rovis T. (2019). J. Am. Chem. Soc..

[cit11] Gaspar B., Carreira E. (2009). J. Am. Chem. Soc..

[cit12] Bao W., Wu X. (2023). J. Org. Chem..

