# Supplementary material for: Photoredox-catalyzed δ-aminomethylation of trifluoroacetamides with oximes as radical traps
Source: RSC Adv. 2026 Jul 17;16(38):41420–3. doi: 10.1039/d6ra04812a (PMC13377984; doi:10.1039/d6ra04812a)
Supplement: Supplementary file 1 [file RA-016-D6RA04812A-s001.pdf]

This journal is © The Royal Society of Chemistry 2026

Supplementary Information

Photoredox-Catalyzed δ-Aminomethylation of Trifluoroacetamides with Oximes as Radical Traps

María Valerio Roa, Melissa A. Ashley, Katherine A. Xie, and Tomislav Rovis*.

Department of Chemistry, Columbia University, New York, NY 10027, United States

*tr2504@columbia.edu

Table of contents

General Information……………………………………………………………………………S-2

Starting Material Synthesis and Characterization Data………………………………………..S-3

Product Synthesis and Characterization Data………………………………………………….S-5

Model for Benzaldehyde Formation from Formaldehyde *O*-Benzyl Oxime……………..……...S-10

Starting material NMR Spectra………………………………………………………………...S-11

Product NMR Spectra………………………………..…………………………………...S-16

References………………………………………………………………………………...S-37

General Information

All reactions were carried out in anhydrous solvents and performed under ambient conditions unless otherwise noted. Commercial reagents and anhydrous solvents were purchased from Sigma-Aldrich and Fisher Scientific. All catalytic reactions were carried out under N_2 in 1dram vials fitted with Teflon caps under irradiation from PR160-427nm Kessil 40W LED lamp. Thin layer chromatography was performed on SiliCycle® 250 µm, 60 Å plates. Chromatographic purification was accomplished by flash chromatography on SiliCycle® Silica Flash® 40-63 µm, 60 Å or Teledyne ISCO Combi*Flash*®*Rf*+ LumenTM instrument using Redi*Sep*®*R*f high performance silica gold column (catalog No. 69-2203-344). Visualization was accomplished with Seebach's "magic" stain.[1] Photocatalysts [Ir(dF-CF_3-ppy)$_2$(dtbbpy)]PF_6[2] and [Ir(dF-Me-ppy)$_2$(dtbbpy)]PF_6[3] were synthesized according to the reported procedures. Unless otherwise noted, ^{1}H NMR (400 MHz), ^{13}C (101 MHz), and ^{19}F (376 MHZ) spectra were taken on a Bruker 500 MHz spectrometer at ambient temperature and recorded in $CDCl_3$. Chemical shifts (δ) are in parts per million relative to $CDCl_3$ (^{1}H: 7.26 ppm, ^{13}C: 77.16 ppm). Data for ^{1}H and ^{13}C NMR is reported as follows: chemical shift (δ ppm), multiplicity (s = singlet, d = doublet, t = triplet, q = quartet, m = multiplet, brs = broad singlet), coupling constant (Hz), integration. High resolution mass spectra (HRMS) were obtained from Columbia University Mass Spectrometry Facility on a JOEL JMSHZ110HF mass spectrometer using ESI^+/$ASAP^+$ ionization model.

Starting material synthesis and characterization data

Trifluoroacetamides were prepared according to literature procedure. These compounds have been previously reported.[4]

General procedure A

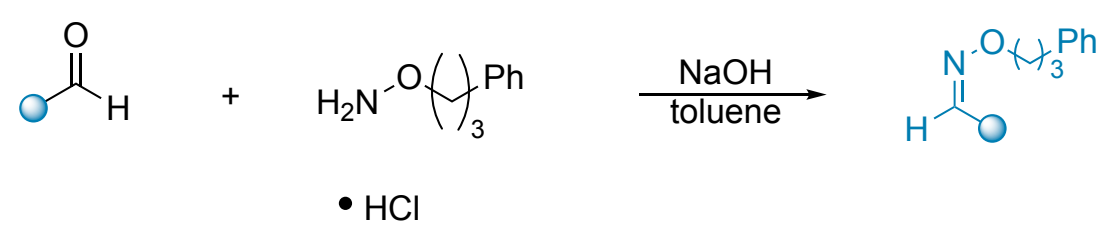

A mixture of hydroxylamine hydrochloride (1.0 equiv), aldehyde (1.0-3.0 equiv), and NaOH (1.05 equiv.) in toluene was stirred at room temperature for 24 h. The organic phase was separated with DCM, washed with sodium bicarbonate, and dried over sodium sulfate The combined organics were concentrated *in vacuo* and purified by flash column chromatography (10% EtOAc/Hex) to afford the desired product.

Formaldehyde *O*-(3-phenylpropyl) oxime

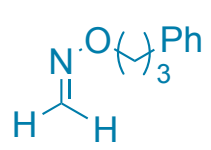

Prepared according to General Procedure A from O-(3-phenylpropyl) hydroxylamine hydrochloride (3.0 g, 15.98 mmol, 1.0 equiv) and 37% formaldehyde in water (1.27 ml, 16.25 mmol, 1.01 equiv). 2.3062 g, 88% yield. **^{1}H NMR (500 MHz, Chloroform-d) δ** 7.31-7.29 (m, 2H), 7.21-7.19 (m, 3H), 7.04 (d, J = 15 Hz, 1H), 6.44 (d, J = 15 Hz, 1H), 4.12 (t, J = 10 Hz, 2H), 2.72 (t, J = 10 Hz, 2H), 2.04-1.94 (m, 2H). Characterization data is consistent with previously reported data.[5]

Formaldehyde *O*-benzyl oxime

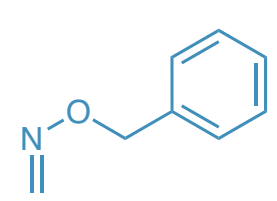

Prepared according to literature procedure from *O*-benzylhydroxylamine hydrogenchloride (2.0g, 12.53 mmol), 37% formaldehyde/H2O solution (1.01 ml, 13.28 mmol), and NaOH (531.2 mg, 13.28 mmol) in toluene (1 M). Colorless oil (99%). **^{1}H NMR** ($CDCl_3$, 300 MHz) δ 7.39-7.28 (m, 5H), 7.09 (d, *J* = 8.2 Hz, 1H), 6.47 (d, *J* = 8.2 Hz, 1H), 5.13 (s, 2H). Characterization data is consistent with previously reported data.[5]

2,3,4,5,6-pentafluorobenzaldehyde *O*-(3-phenylpropyl) oxime

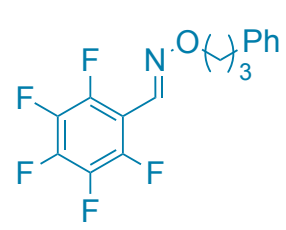

Prepared according to General Procedure A from O-(3-phenylpropyl) hydroxylamine hydrochloride (200 mg, 1.32 mmol, 1.0 equiv) and pentafluorobenzaldehyde (162.98µl, 1.32 mmol, 1.0 equiv). **^{1}H NMR** ($CDCl_3$, 500 MHz) δ 8.15 (s, 1H), 7.31-7.28 (m, 2H), 7.22-7.18 (m, 3H), 4.26 (t, *J* = 6.5 Hz, 2H), 2.74, (dd, *J* = 8.6, 6.8 Hz, 2H), 2.09-2.03 (m, 2H). **^{13}C**

NMR (126 MHz, $CDCl_3$) δ 146.07 (apparent m), 144.07 (apparent m), 142.56 (apparent m), 141.69, 140.51 (apparent m), 138.96 (apparent m), 137.24 (apparent m), 136.96 (apparent m) 128.62, 128.55, 126.07, 107.98 (td, *J* = 13.3, 4.4 Hz), 74.60, 32.15, 30.80. ^{19}**F NMR** (471MHz, $CDCl_3$) δ -139.29 – -139.27 (m), - 151.44 (apparent tt, *J* = 20.8, 2.5 Hz), -160.8 – -160.92 (m). **HRMS-ASAP** (positive) M = $C_{16}H_{12}F_5NO$: calculated $(M+H)^+$ *m/z* 330.0917; found $(M+H)^+$ *m/z* 330.0918.

Pyrazine-2-carbaldehyde *O*-(3-phenylpropyl) oxime

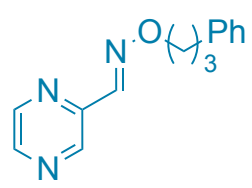

Prepared according to General Procedure A from O-(3-phenylpropyl) hydroxylamine hydrochloride (500 mg, 2.67 mmol, 1.0 equiv) and pyrazine -2-carbaldehyde (280.95 µl, 3.20 mmol, 1.2 equiv). 288.2 mg, 75% yield. ^{1}**H NMR** ($CDCl_3$, 500 MHz) δ 9.05 (d, *J* = 1.5 Hz, 1H), 8.55 (dd, *J* = 2.6, 1.6 Hz, 1H), 8.52 (d, *J* = 2.6 Hz, 1H), 8.17 (s, 1H), 7.32-7.27 (s, 2H), 7.23-7.17 (s, 3H), 4.29 (t, *J* = 6.5 Hz, 2H), 2.75 (dd, *J* = 8.6, 6.8 Hz, 2H), 2.12-2.05 (m, 2H). ^{13}**C NMR** (126 MHz, $CDCl_3$) δ 147.77, 146.94, 144.40, 144.27, 143.05, 141.67, 128.61, 128.55, 126.07, 74.57, 32.16, 30.83. **HRMS-ASAP** (positive) M = $C_{14}H_{15}F_3N_3O$: calculated $(M+H)^+$ *m/z* 242.1293; found $(M+H)^+$ *m/z* 242.1290.

2,2,2-trifluoroacetaldehyde *O*-(3-phenylpropyl) oxime

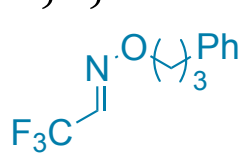

Prepared according to General Procedure A from O-(3-phenylpropyl) hydroxylamine hydrochloride (200 mg, 1.32 mmol, 1.0 equiv) and trifluoroacetaldehyde (176µl, 1.32 mmol, 1.0 equiv). ^{1}**H NMR** ($CDCl_3$, 400 MHz) δ 7.32-7.27 (m, 2H), 7.22-7.18 (m, 3H), 5.72 (d, *J* = 6.9 Hz, 1H), 3.79 (t, *J* = 6.5 Hz, 2H), 2.67 (dd, *J* = 8.5, 6.9 Hz, 2H), 1.96-1.89 (m, 2H) (impurity present). ^{13}**C NMR** (101 MHz, $CDCl_3$) δ 141.72, 128.56, 128.52, 126.12, 122.30 (q, *J* = 282.7 Hz), 81.58 (q, *J* = 32.62 Hz), 74.63, 32.38, 29.98. ^{19}**F NMR** (376 MHz, $CDCl_3$) δ -79.00, -79.01. **HRMS-ASAP** (positive) M = $C_{11}H_{12}F_3NO$: calculated $(M+H)^+$ *m/z* 232.0949; found $(M+H)^+$ *m/z* 232.0956.

Product synthesis and characterization data

General procedure B

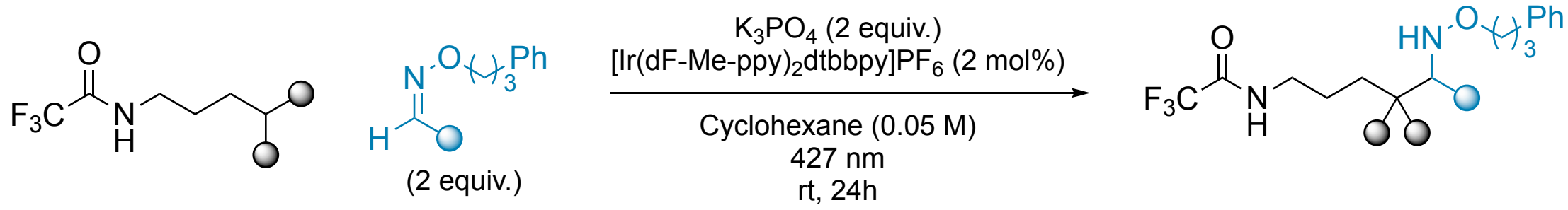

Trifluoroacetamide (0.1 mmol, 1.0 eq.), oxime (0.2 mmol, 2.0 eq.), and [Ir(dF-Me-ppy)$_2$dtbbpy]PF_6 (2 mol%, 2.0 mg) were added to a 1 dram vial. Potassium phosphate tribasic (0.2 mmol, 2.0 eq., 42.5 mg) and cyclohexane (0.05 M, 2 mL) were added under an atmosphere of nitrogen. The resulting solution was allowed to stir illuminated with blue LED (Kessil, 160 W, 427 nm) for 24 hours. The crude was concentrated *in vacuo* and purified by flash column chromatography (5-25% ethyl acetate/hexanes) to afford the desired product.

2,2,2-trifluoro-N-(2,5,5-trimethyl-6-((3-phenylpropoxy)amino)hexan-2-yl)acetamide (1)

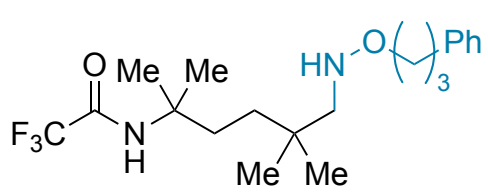

Prepared according to General Procedure B from N-(2,5-dimethylhexan-2-yl)-2,2,2-trifluoroacetamide (22.7 mg, 0.101 mmol) and formaldehyde O-(3-phenylpropyl) oxime (32.6 mg, 0.2 mmol). 75% yield. **^{1}H NMR** ($CDCl_3$, 400 MHz) δ 7.31-7.26 (m, 2H), 7.21-7.15 (m, 3H), 6.32 (brs, 1H), 5.42 (brs, 1H), 3.67 (t, J = 6.5 Hz, 2H), 2.76 (s, 2H), 2.69-2.65 (m, 2H), 1.92-1.85 (m, 2H), 1.68-1.64 (m, 2H), 1.38 (s, 6H), 1.25-1.21 (m, 2H), 0.91 (s, 6H). **^{13}C NMR** (101 MHz, $CDCl_3$) δ 156.28 (q, J = 35.8 Hz), 142.10, 128.52, 128.46, 125.93, 115.82 (q, J = 289.5 Hz), 72.84, 60.91, 55.40, 34.61, 33.71, 33.05, 33.52, 30.48, 26.47, 26.23. **^{19}F NMR** (376 MHz, $CDCl_3$) δ -75.92. **HRMS-ESI** (positive) M = $C_{20}H_{31}F_3N_2O_2$: calculated $(M+H)^+$ *m/z* 389.2416; found $(M+H)^+$ *m/z* 389.2421.

N-(5,5-dimethyl-6-((3-phenylpropoxy)amino)hexan-2-yl)-2,2,2-trifluoroacetamide (2)

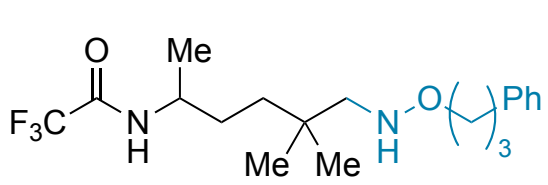

Prepared according to General Procedure B from 2,2,2-trifluoro-N-(pentan-2-yl)acetamide (18.3 mg, 0.1 mmol) and formaldehyde O-(3-phenylpropyl) oxime (33.1 mg, 0.2 mmol). 82% yield. **^{1}H NMR (500 MHz, $CDCl_3$)** δ 7.31-7.26 (m, 2H), 7.21-7.16 (m, 3H), 6.22 (brs, 1H), 3.93 (hept, J = 6.9 Hz, 1H), 3.67 (t, J = 6.5 Hz, 2H), 2.78-2.71(m, 2H), 2.66 (dd, J = 8.8, 6.8 Hz, 2H), 1.92-1.85 (m, 2H), 1.54-1.44 (m, 2H), 1.29-1.24 (m, 2H), 1.21 (d, J = 6.5 Hz, 3H), 0.90 (s, 6H). **^{13}C NMR (126 MHz, $CDCl_3$)** δ 156.42 (apparent m), 141.96, 128.37 (d, 7.56 Hz), 117.05, 114.76, 72.82, 61.04, 47.20, 35.91, **^{19}F NMR (471 MHz, $CDCl_3$)** δ -74.98. **HRMS-ESI** (positive) M = $C_{19}H_{29}F_3N_2O_2$: calculated $(M+H)^+$ *m/z* 375.2260; found $(M+H)^+$ *m/z* 375.2259.

N-(4,4-dimethyl-5-((3-phenylpropoxy)amino)pentyl)-2,2,2-trifluoroacetamide (3)

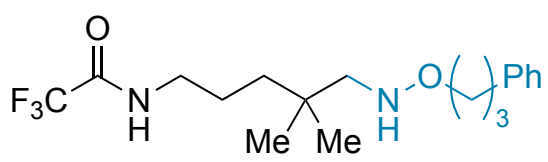

Prepared according to General Procedure B from 2,2,2-trifluoro-N-(4-methylpentyl)acetamide (19.7 mg, 0.1 mmol) and formaldehyde O-(3-phenylpropyl) oxime (32.6 mg, 0.2 mmol). 67% yield. **^{1}H NMR** (500 MHz, $CDCl_3$) δ 7.31-7.28 (m, 2H), 7.21-7.17 (m, 3H), 6.49 (brs, 1H), 3.70-3.66 (m, 2H), 3.33 (q, J=10.00, 5.00 Hz, 2H), 2.75 (s, 2H), 2.73-2.65 (m, 2H), 1.97-1.86 (m, 2H), 1.59-1.48 (m, 2H), 1.34-1.26 (m, 2H), 0.91 (s, 6H). **^{13}C NMR** (126 MHz, $CDCl_3$) δ 157.34, 141.97, 128.43-128.30 (m), 125.82, 72.81, 61.20, 40.62, 36.98, 34.24, 33.21, 32.38, 32.09, 30.32, 29.71, 26.06, 23.55**.** **^{19}F NMR** (471 MHz, $CDCl_3$) δ -74.94. **HRMS-ESI** (positive) M = $C_{18}H_{28}F_3N_2O_2$: calculated $(M+H)^+$ *m/z* 361.2103; found $(M+H)^+$ *m/z* 361.2095.

N-(4-ethyl-4-(((3-phenylpropoxy)amino)methyl)octyl)-2,2,2-trifluoroacetamide (4)

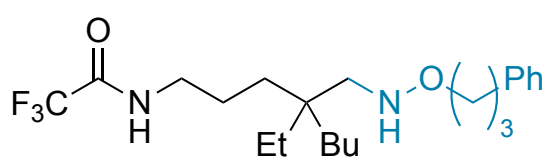

Prepared according to General Procedure B from N-(4-ethyloctyl)-2,2,2-trifluoroacetamide (30.2 mg, 0.101 mmol) and formaldehyde O-(3-phenylpropyl) oxime (33.13 mg, 0.203 mmol). 61% yield. **^{1}H NMR** ($CDCl_3$, 400 MHz) δ 7.31-7.26 (m, 2H), 7.21-7.16 (m, 3H), 6.59 (brs, 1H), 5.35 (brs, 1H), 3.66 (t, J = 6.5 Hz, 2H), 3.31 (q, J = 6.7 Hz, 2H), 2.76 (s, 2H), 2.69-2.65 (m, 2H), 1.94-1.84 (m, 2H), 1.55-1.48 (m, 2H), 1.32-1.12 (m, 10H), 0.91 (t, J = 7.2 Hz, 3H), 0.79 (t, J = 7.5 Hz, 3H). **^{13}C NMR** (101 MHz, $CDCl_3$) δ 157.34 (q, J = 36.8 Hz), 142.08, 128.53, 128.47, 125.94, 116.04 (q, J = 287.9 Hz), 72.93, 56.25, 40.77, 37.91, 34.65, 32.49, 32.05, 30.44, 27.60, 25.20, 23.62, 22.68, 14.23, 7.52. **^{19}F NMR** (376 MHz, $CDCl_3$) δ -75.82. **HRMS-ESI** (positive) M = $C_{22}H_{35}F_3N_2O_2$: calculated $(M+H)^+$ *m/z* 417.27298; found $(M+H)^+$ *m/z* 417.2739.

N-(4,8-dimethyl-4-(((3-phenylpropoxy)amino)methyl)nonyl)-2,2,2-trifluoroacetamide (5)

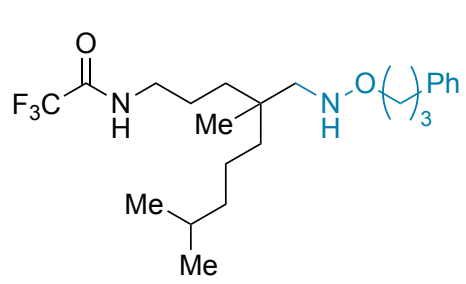

Prepared according to General Procedure B from N-(4,8-dimethylnonyl)-2,2,2-trifluoroacetamide (27.0 mg, 0.101 mmol) and formaldehyde O-(3-phenylpropyl) oxime (33.13 mg, 0.203 mmol). 48% yield. **^{1}H NMR** ($CDCl_3$, 400 MHz) δ 7.31-7.26 (m, 2H), 7.21-7.17 (m, 3H), 6.56 (brs, 1H), 5.38 (brs, 1H), 3.66 (t, J =6.5 Hz, 1H), 3.32 (q, J = 6.7 Hz, 1H), 2.80-2.73, m, 2H), 2.69-2.65 (m, 2H), 1.92-1.85 (m, 2H), 1.59-1.49 (m, 3H), 1.30-1.11 (m, 8H), 0.88-0.86 (m, 9H). **^{13}C NMR** (101 MHz, $CDCl_3$) δ 157.32 (q, J = 36.7 Hz), 142.08, 128.53, 128.46, 125.94, 116.03 (q, J = 287.9 Hz), 72.92, 59.20, 40.77, 39.92, 38.80, 35.69, 35.00, 32.50, 30.44, 28.02, 23.81, 23.23, 22.75, 21.22**.** **^{19}F NMR** (376 MHz, $CDCl_3$) δ -75.83. **HRMS-ASAP (positive)** M = $C_{23}H_{37}F_3N_2O_2$: calculated (M+H)+ *m/z* 431.2885; found (M+H)+ m/z 431.2893

2,2,2-trifluoro-N-(3-(1-(((3-phenylpropoxy)amino)methyl)cyclopentyl)propyl)acetamide (6)

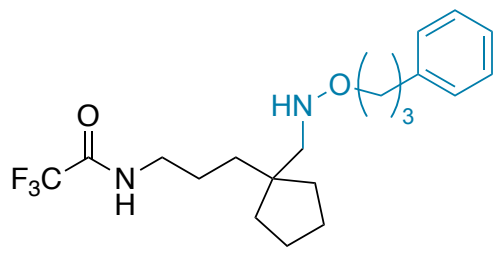

Prepared according to General Procedure B from N-(3-cyclopentylpropyl)-2,2,2-trifluoroacetamide (22.3 mg, 0.1 mmol) and formaldehyde O-(3-phenylpropyl) oxime (32.6 mg, 0.2 mmol). 45% yield. **^{1}H NMR** (500 MHz, $CDCl_3$) δ 7.36-7.32 (m, 2H), 7.25-7.23 (m, 3H), 6.61 (brs, 1H), 3.73 (t, J = 10.00, 2H), 3.40 (q, J = 10.00, 5.00 Hz, 2H), 2.90 (s, 2H), 2.73 (t, J = 10.00 Hz, 2H), 1.97-1.93 (m, 2H), 1.69-1.66 (m, 2H), 1.65-1.61 (m, 3H), 1.54-1.49 (m, 2H), 1.45-1.40 (m, 4H), 1.32 (s, 1H). **^{13}C NMR** (126 MHz, $CDCl_3$) δ 157.32, 157.03, 141.94, 128.38 (d, J = 6.30), 125.82, 117.06, 114.77, 72.87, 58.40, 44.60, 40.59, 36.68, 34.46, 32.36, 30.33, 24.69, 24.15. **^{19}F NMR** (471 MHz, $CDCl_3$) δ -74.90. **HRMS-ESI** (positive) M = $C_{20}H_{30}F_3N_2O_2$: calculated $(M+H)^+$ *m/z* 387.2260; found $(M+H)^+$ *m/z* 387.2264.

N-(5-((tert-butyldimethylsilyl)oxy)-4-methyl-4-(((3-phenylpropoxy)amino)methyl)pentyl)-2,2,2-trifluoroacetamide (7)

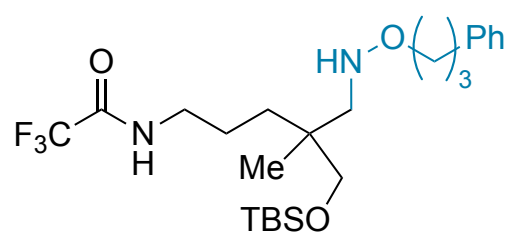

Prepared according to General Procedure B from N-(5-((tert butyldimethylsilyl)oxy)-4-methylpentyl)-2,2,2-trifluoroacetamide (32.7 mg, 0.1 mmol) and formaldehyde O-(3-phenylpropyl) oxime (32.6 mg, 0.2 mmol). 45% yield. **^{1}H NMR** (500 MHz, $CDCl_3$) δ 7.30-7.26 (m, 2H), 7.20-7.17 (m, 3H), 6.76 (brs, 1H), 3.66-3.63 (m, 2H), 3.38-3.30 (m, 4H), 2.86 (s, 2H), 2.66 (t, J = 12.5 Hz, 2H), 1.89-1.85 (m, 2H), 1.63-1.56 (m, 2H), 1.51-1.47 (m, 1H), 1.26-1.18 (m, 2H), 0.89 (s, 9H), 0.82 (s, 3H), 0.07-0.02 (m, 5H). **^{13}C NMR** (126 MHz, $CDCl_3$) δ 157.55, 157.25, 142.09, 128.51 (d, J = 10.08 Hz), 125.94, 117.20, 114.91, 72.71, 70.68, 58.21, 40.73, 38.16, 32.49, 31.82, 30.49, 25.95, 22.80, 20.97, 18.28, -5.50. (d, J = 6.30 Hz). **^{19}F NMR** (471 MHz, $CDCl_3$) δ -74.88. **HRMS-ESI (positive)** M = $C_{24}H_{41}F_3N_2O_3Si$: calculated $(M+H)^+$ *m/z* 491.2917; found $(M+H)^+$ *m/z* 491.2932.

2,2,2-trifluoro-*N*-(2-((2-methyl-1-((3-phenylpropoxy)amino)propan-2-yl)oxy)ethyl)acetamide (8)

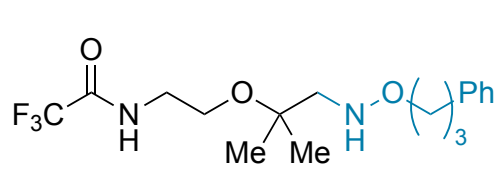

Prepared according to General Procedure B from 2,2,2-trifluoro-*N*-(2-isopropoxyethyl)acetamide (20.1 mg, 0.101 mmol) and formaldehyde *O*-(3-phenylpropyl) oxime (33.13 mg, 0.2 mmol). 66% yield. ^{1}**H NMR** (CDCl3, 400 MHz) δ 7.30-7.26 (m, 2H), 7.20-7.17 (m, 3H), 5.62 (brs, 1H), 3.69 (t, *J* = 6.5 Hz, 2H), 3.54-3.48 (m, 4H), 2.97 (s, 2H), 2.66 (dd, *J* = 8.8, 6.8 Hz, 2H), 1.92-1.85 (m, 2H), 1.21 (s, 6H). **^{13}C NMR** (101 MHz, CDCl3) δ 157.35, (q, *J* = 37.0 Hz), 142.01, 128.50, 128.47, 125.95, 116.05 (q, *J* = 287.7 Hz), 74.67, 73.06, 59.46, 59.37, 40.50, 32.48, 30.41, 24.55. **^{19}F NMR** (376 MHz, $CDCl_3$) δ -75.75. **HRMS-ASAP** (positive) M = $C_{17}H_{25}F_3N_2O_3$: calculated (M+H)+ *m/z* 363.1895; found (M+H)+ *m/z* 363.1895.

N-(2,5-dimethyl-6-((3-phenylpropoxy)amino)hexan-2-yl)-2,2,2-trifluoroacetamide (9)

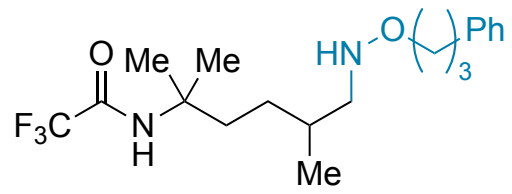

Prepared according to General Procedure B from 2,2,2-trifluoro-N-(2-methylhexan-2-yl)acetamide (21.1 mg, 0.1 mmol) and formaldehyde O-(3-phenylpropyl) oxime (32.6 mg, 0.2 mmol). 48% yield. **^{1}H NMR** ($CDCl_3$, 400 MHz) δ 7.29-7.27 (m, 2H), 7.19-7.16 (m, 3H), 6.06 (brs, 1H), 3.68 (t, J=6.0 Hz, 2H), 2.83-2.72 (m, 2H), 2.66 (t, J=6.0 Hz, 2H), 1.91-1.86 (m, 2H), 1.83-1.77 (m, 1H), 1.74-1.64 (m, 3H), 1.38 (d, J=4.0 Hz, 6H), 1.15-1.08 (m, 1H), 0.93 (d, J=4.0 Hz, 3H). **^{13}C NMR** (101 MHz, $CDCl_3$) δ 155.89 (apparent m), 141.95, 128.35 (d, J=9.09 Hz), 125.76, 116.78, 73.12, 58.02, 55.33, 36.95, 32.35, 30.90, 30.33, 29.68, 28.59, 26.29, 26.25, 18.06. **^{19}F NMR** (376 MHz, $CDCl_3$) δ -75.17.

N-(4-((tert-butyldimethylsilyl)oxy)-5-((3-phenylpropoxy)amino)pentyl)-2,2,2-trifluoroacetamide (10)

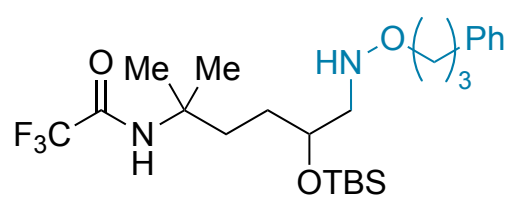

Prepared according to General Procedure N-(4-((tert-butyldimethylsilyl)oxy)butyl)-2,2,2-trifluoroacetamide (30.2 mg, 0.101 mmol) and formaldehyde O-(3-phenylpropyl) oxime (33.1 mg, 0.2 mmol). 31% yield. **^{1}H NMR** ($CDCl_3$, 400 MHz) δ 7.30-7.27 (m, 2H), 7.20-7.16 (m, 3H), 6.46 (brs, 1H), 5.67 (brs, 1H), 3.96 (dq, J = 6.8, 5.1 Hz, 1H), 3.66 (t, J = 6.5 Hz, 2H), 3.37 (dq, J = 31.3, 6.6 Hz, 2H), 3.01-2.82 (m, 2H), 2.66 (dd, J = 8.8, 6.8 Hz, 2H), 1.91-1.84 (m, 2H), 1.68-1.61 (m, 2H), 1.57-1.50 (m, 2H), 0.90 (s, 9H), 0.11 (s, 3H), 0.08 (s, 3H). **^{13}C NMR** (101 MHz, $CDCl_3$) δ 157.15 (q, J = 37 Hz), 141.96, 1.28.40, 128.34, 125.81, 115.88 (q, J = 287.9 Hz), 72.90, 68.46, 47.07, 40.02, 32.62, 32.38, 20.35, 25.83, 24.36, 18.08, -4.55, -4.66. **^{19}F NMR** (376 MHz, CDCl3) δ -75.89. **HRMS-ASAP** (positive) M = $C_{22}H_{37}F_3N_2O_3Si$: calculated $(M+H)^+$ m/z 463.2604; found $(M+H)^+$ *m/z* 463.2599.

2,2,2-trifluoro-N-(2-(2-(((3-phenylpropoxy)amino)methyl)cyclohexyl)ethyl)acetamide (11)

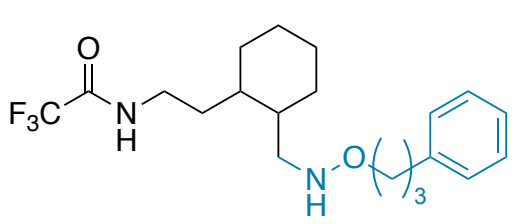

Prepared according to General Procedure B from N-(2-cyclohexylethyl)-2,2,2-trifluoroacetamide (22.3 mg, 0.1 mmol) and formaldehyde O-(3-phenylpropyl) oxime (33.1 mg, 0.2 mmol). 30% yield, 2:1 dr. **^{1}H NMR** (500 MHz, $CDCl_3$) δ 7.29-7.26 (m, 2H), 7.22-7.18 (m, 3H), 6.50 (brs, 1H), 4.06-4.02 (m, 2H), 3.41-3.33 (m, 2H), 2.70-2.67 (m, 2H), 1.97-1.95 (m, 2H), 1.88-1.86 (m, 1H), 1.76-1.73 (m, 4H), 1.43-1.33 (m, 3H), 1.26-1.22 (m, 4H), 1.08-1.03 (m, 1H), 0.89-0.87 (m, 1H).**^{13}C NMR** (126 MHz, $CDCl_3$) δ 141.89, 128.39 (d, J = 6.30 Hz), 125.84, 72.95, 55.39, 39.45, 39.15, 37.80 (d, J = 5.04 Hz), 32.23, 32.15, 31.65, 31.07, 30.32, 29.71, 29.38, 29.02, 25.71 (d, J = 2.52), 14.13. **^{19}F NMR** (471 MHz, $CDCl_3$) δ -74.84. **HRMS-ESI (positive)** M = $C_{20}H_{30}F_3N_2O_2$: calculated $(M+H)^+$ m/z 387.2259; found $(M+H)^+$ m/z 387.2245.

2,2,2-trifluoro-N-(6,6,6-trifluoro-4,4-dimethyl-5-((3-phenylpropoxy)amino)hexyl)acetamide (12)

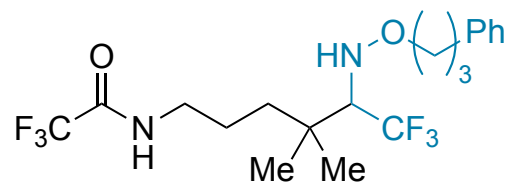

Prepared according to General Procedure B from 2,2,2-trifluoro-*N*-(4-methylpentyl)acetamide (20.0 mg, 0.101 mmol) and 2,2,2-trifluoroacetaldehyde *O*-(3-phenylpropyl) oxime (46.9 mg, 0.203 mmol). 13.1 mg, 31% yield. ^{1}**H NMR** ($CDCl_3$, 400 MHz) δ 7.31-7.27 (m, 2H), 7.21-7.16 (m, 3H), 6.29 (brs, 1H), 3.74-3.64 (m, 2H), 3.32 (q, *J* = 6.6 Hz, 1H), 3.16 (q, *J* = 8.7 Hz, 1H), 2.66 (dd, *J* = 8.5, 6.9 Hz, 1H), 1.92-1.85 (m, 2H), 1.61-1.33 (m, 4H), 1.03 (m, 6H). ^{13}**C NMR** (101 MHz, $CDCl_3$) δ 157.36 (q, *J* = 37.0 Hz), 141.95, 128.56, 128.51, 126.72 (q, *J* = 286.0 Hz), 126.00, 115.98 (q, *J* = 287.9 Hz), 73.45, 68.37 (q, *J* = 24.7 Hz), 40.50, 37.56, 37.54, 35.78, 32.38, 30.00, 24.88 (q, *J* = 2.0 Hz), 24.71 (q, *J* = 2.5 Hz), 23.63. ^{19}**F NMR** (376 MHz, $CDCl_3$) δ -66.05, -66.08, -75.92.

N-(4,4-dimethyl-5-(perfluorophenyl)-5-((3-phenylpropoxy)amino)pentyl)-2,2,2-trifluoroacetamide (13)

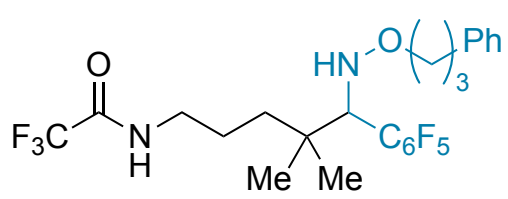

Prepared according to General Procedure B from 2,2,2-trifluoro-*N*-(4-methylpentyl)acetamide (20.0 mg, 0.101 mmol) and 2,3,4,5,6-pentafluorobenzaldehyde *O*-(3-phenylpropyl) oxime (66.8 mg, 0.203 mmol). 34% yield. ^{1}**H NMR** ($CDCl_3$, 400 MHz) δ 7.29-7.25 (m, 2H), 7.21-7.12 (m, 3H), 6.26 (brs, 1H), 4.35 (s, 1H), 3.64 (t, *J* = 6.4 Hz, 2H), 3.33 (qq, *J* = 13.4, 6.6 Hz, 2H), 2.57 (dd, *J*=8.5, 6.8, 2H), 1.87-1.80 (m, 2H), 1.66-1.543 (m, 4H), 1.38-1.29 (m, 1H), 0.89 (md, 6H). ^{13}**C NMR** (101 MHz, $CDCl_3$) δ 157.19 (d, J = 37.37 Hz), 141.75, 128.33, 125.84, 117.28, 114.41, 113.97, 72.98, 64.34, 40.46, 37.40, 36.91, 32.11, 30.70, 29.94, 24.50, 23.92 (d, J=4.04 Hz), 23.63. ^{19}**F NMR** (376 MHz, $CDCl_3$) δ -75.89, -139.88 (dd, J=7.52, 7.52 Hz), -142.07, -155.39 (t, J=18.8 Hz), -161.92 – -162.22 (m). **HRMS-ASAP** (positive) M = $C_{24}H_{26}F_8N_2O_2$: calculated $(M+H)_+$ *m/z* 527.1945; found $(M+H)_+$ *m/z* 527.1927.

N-(4,4-dimethyl-5-((3-phenylpropoxy)amino)-5-(pyrazin-2-yl)pentyl)-2,2,2-trifluoroacetamide (14)

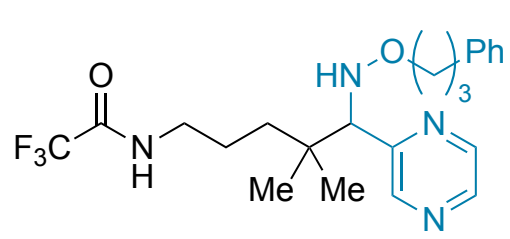

Prepared according to General Procedure B from 2,2,2-trifluoro-N-(4-methylpentyl)acetamide (19.7 mg, 0.1 mmol) and pyrazine-2-carbaldehyde O-(3-phenylpropyl) oxime (120.5 mg, 0.5 mmol). 32% yield. ^{1}**H NMR** (400 MHz, $CDCl_3$) δ 8.57 (dd, J = 2.6, 1.5 Hz, 1H), 8.52 (d, J = 1.5 Hz, 1H), 8.49 (d, J = 2.5 Hz, 1H), 7.28-7.24 (m, 2H), 7.19-7.15 (m, 1H), 7.12-7.10 (m, 2H) 6.45 (s, 1H), 3.93 (s, 1H), 3.66 (qt, J = 10.0, 6.5 Hz, 2H), 3.32 (qd, J = 6.9, 2.9 Hz, 2H), 2.52 (dd, J = 8.7, 6.8 Hz, 2H), 1.85-1.78 (m, 2H), 1.64 (dd, J = 13.3, 6.7, 2.4 Hz, 2H), 1.48–1.31 (m, 2H), 0.88 (d, J = 22.7 Hz, 6H). ^{13}**C NMR** (101 MHz, $CDCl_3$) 157.40, 157.03, 155.68, 145.73, 143.62, 143.31, 141.79, 128.34 (d, J = 3.03 Hz), 125.81, 117.31, 114.45, 73.01, 70.23, 40.54, 36.77 (d, J = 6.06 Hz), 32.13, 29.99, 24.68, 23.68, 23.47. ^{19}**F NMR** (376 MHz, $CDCl_3$) δ -75.80. **HRMS-ASAP** (positive) M = $C_{22}H_{29}F_3N_4O_2$: calculated $(M+H)^+$ *m/z* 439.2321; found $(M+H)^+$ *m/z* 439.2319.

Model for Benzaldehyde Formation from Formaldehyde *O*-Benzyl Oxime

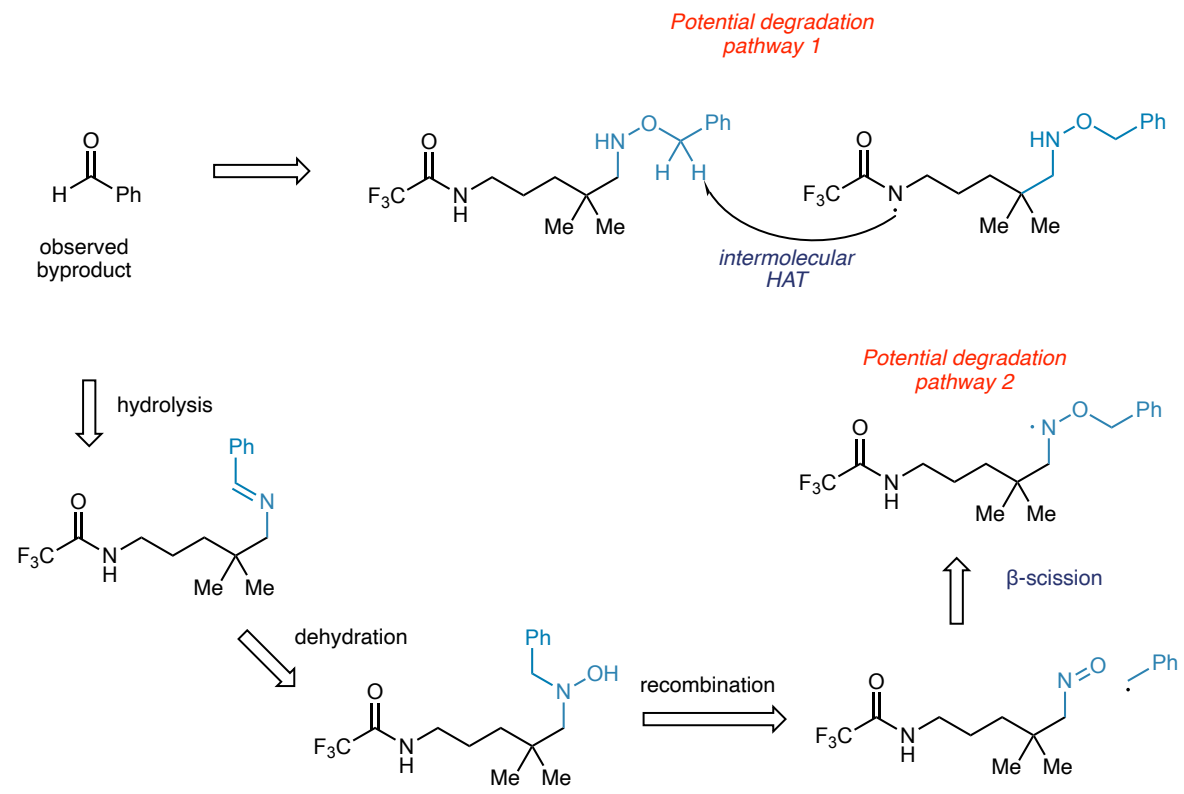

Starting Material NMR Spectra

Formaldehyde *O*-(3-phenylpropyl) oxime

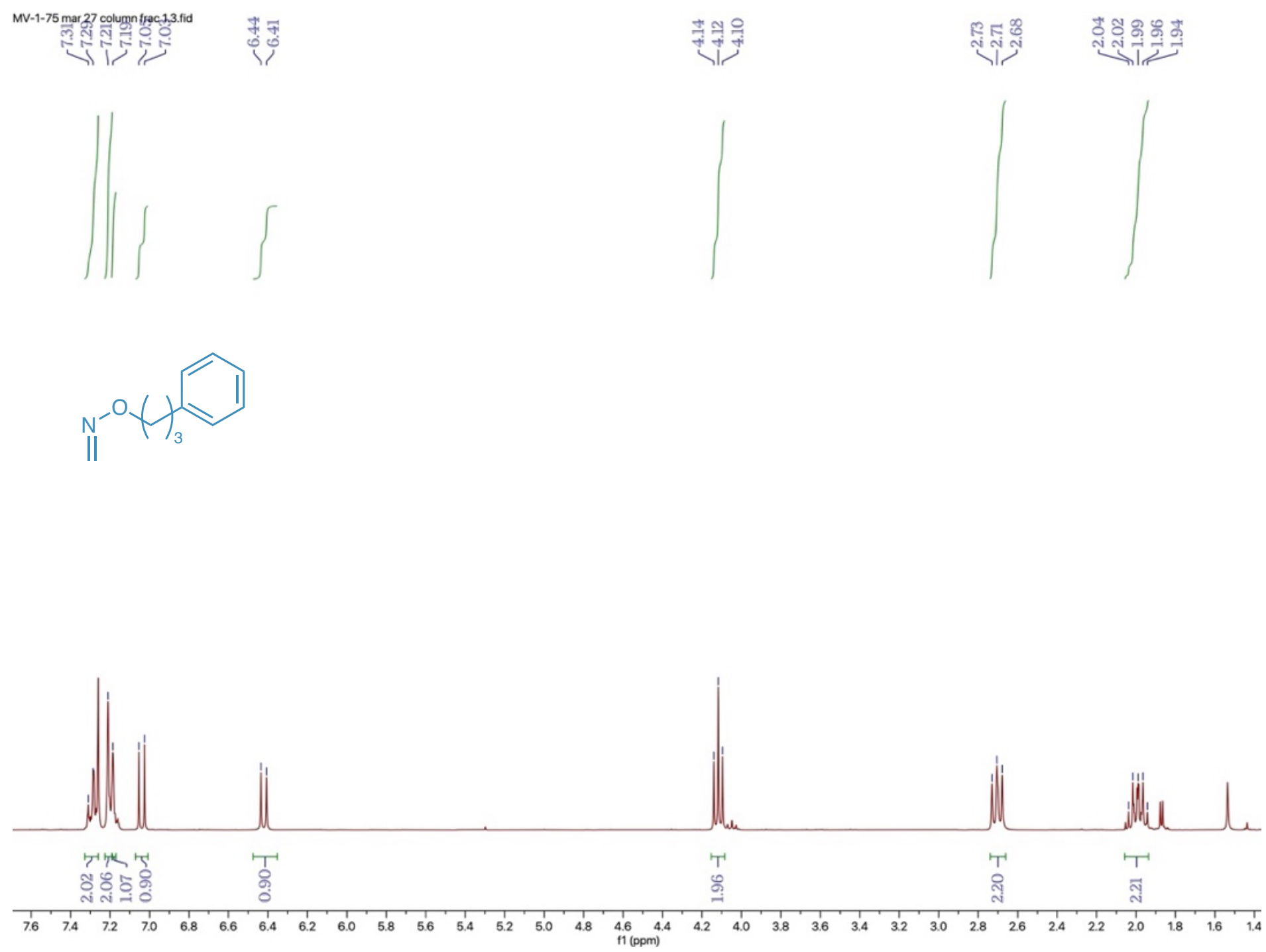

Formaldehyde *O*-benzyl oxime

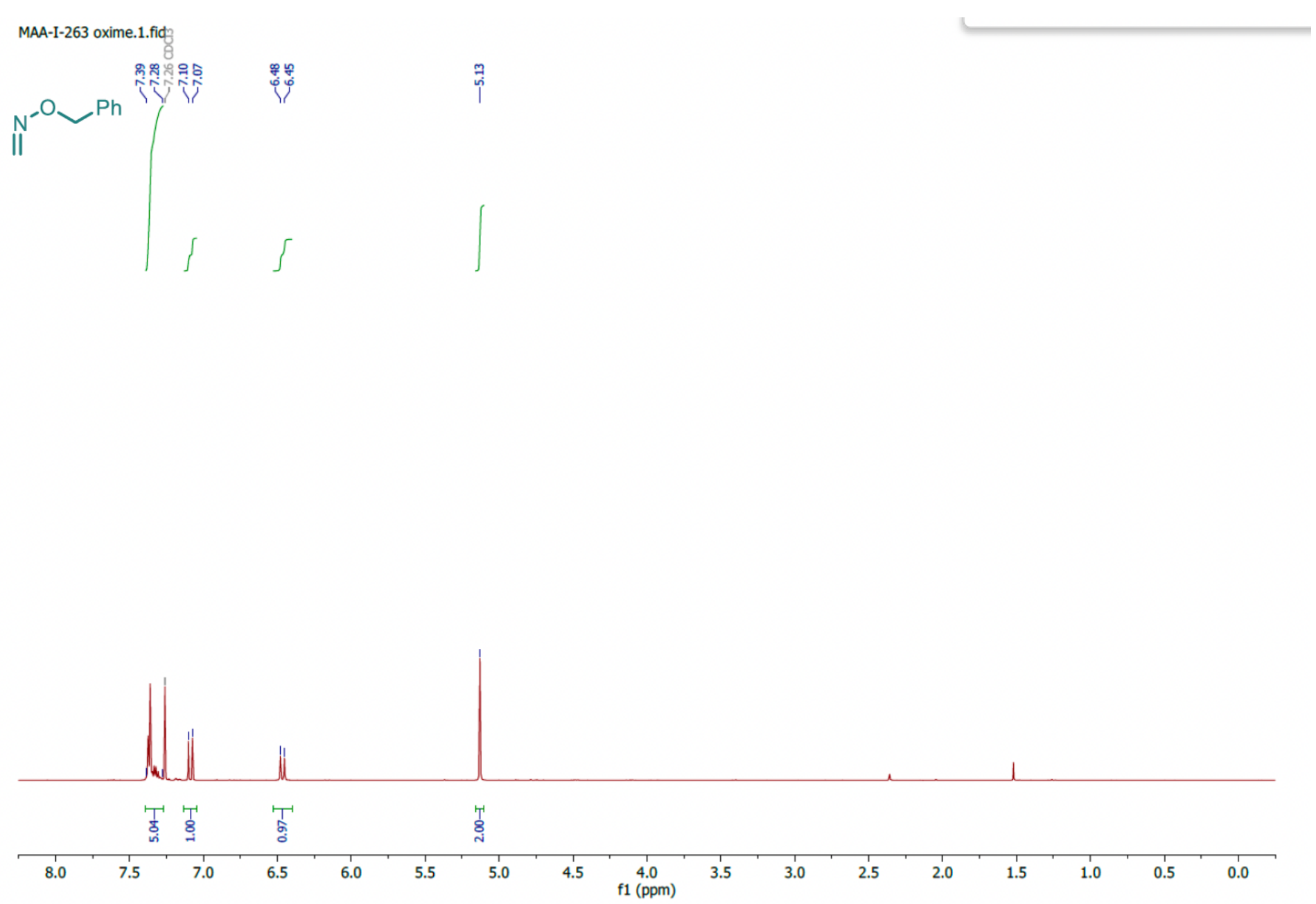

2,3,4,5,6-pentafluorobenzaldehyde *O*-(3-phenylpropyl) oxime

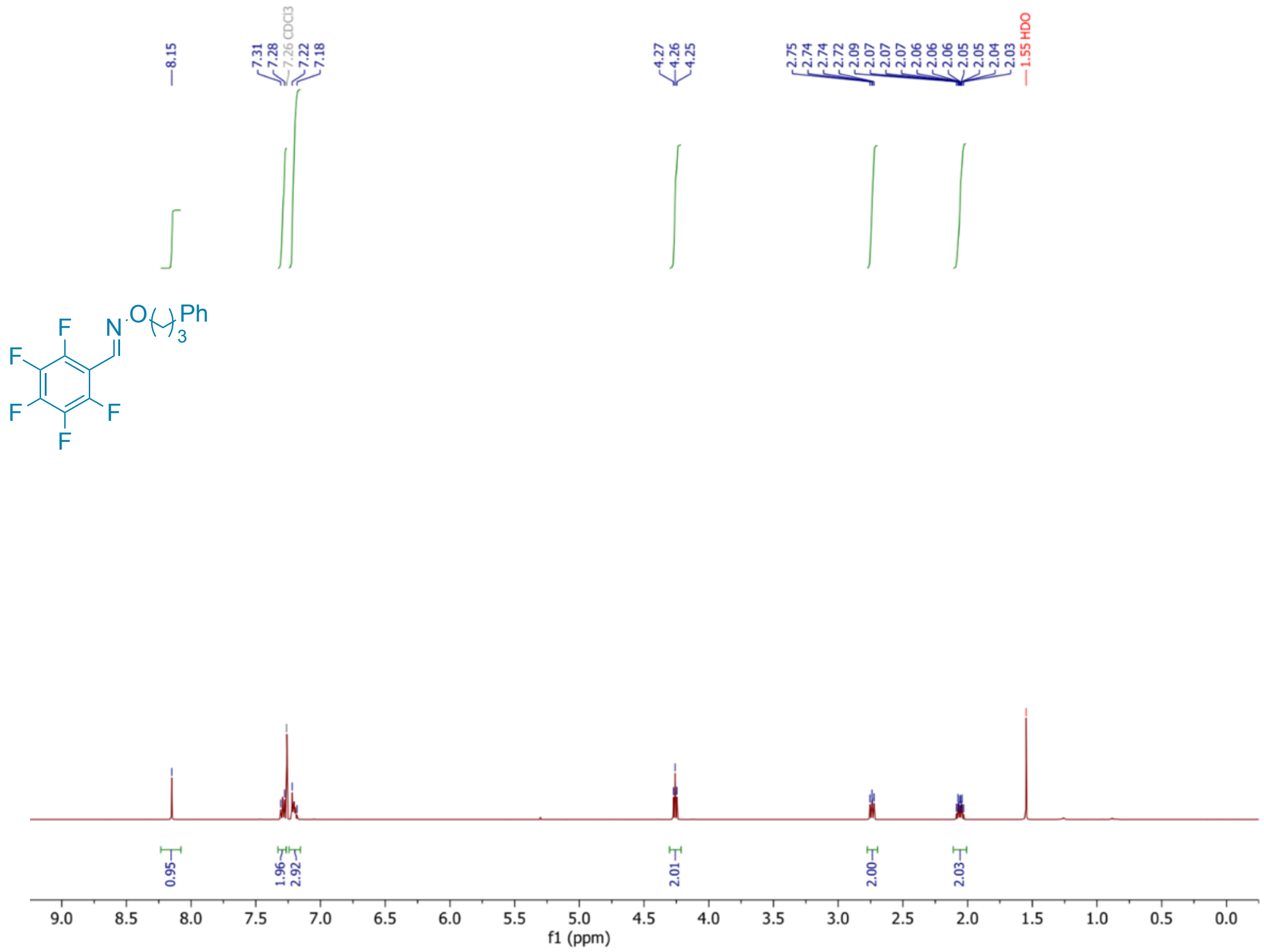

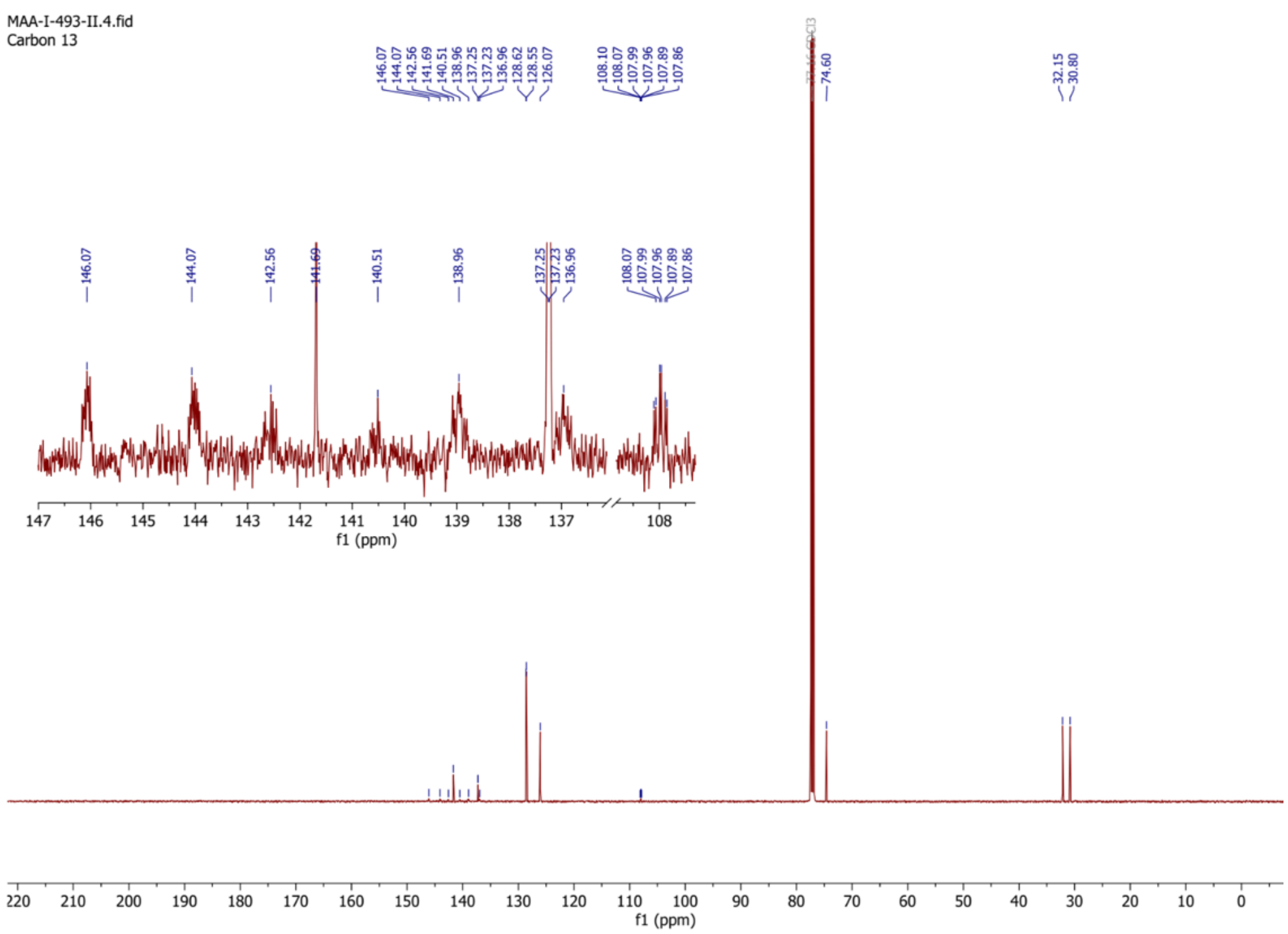

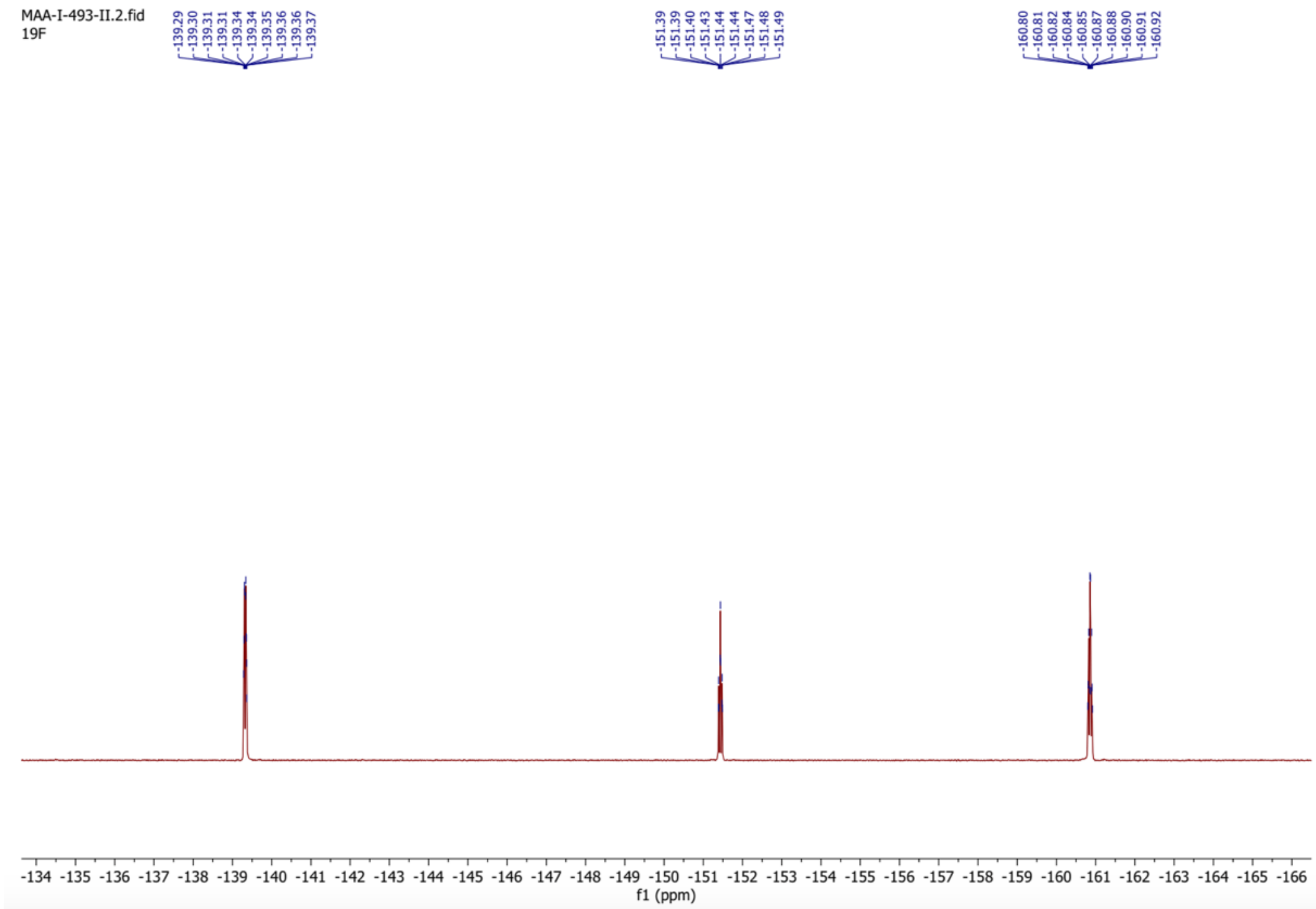

Pyrazine-2-carbaldehyde *O*-(3-phenylpropyl) oxime

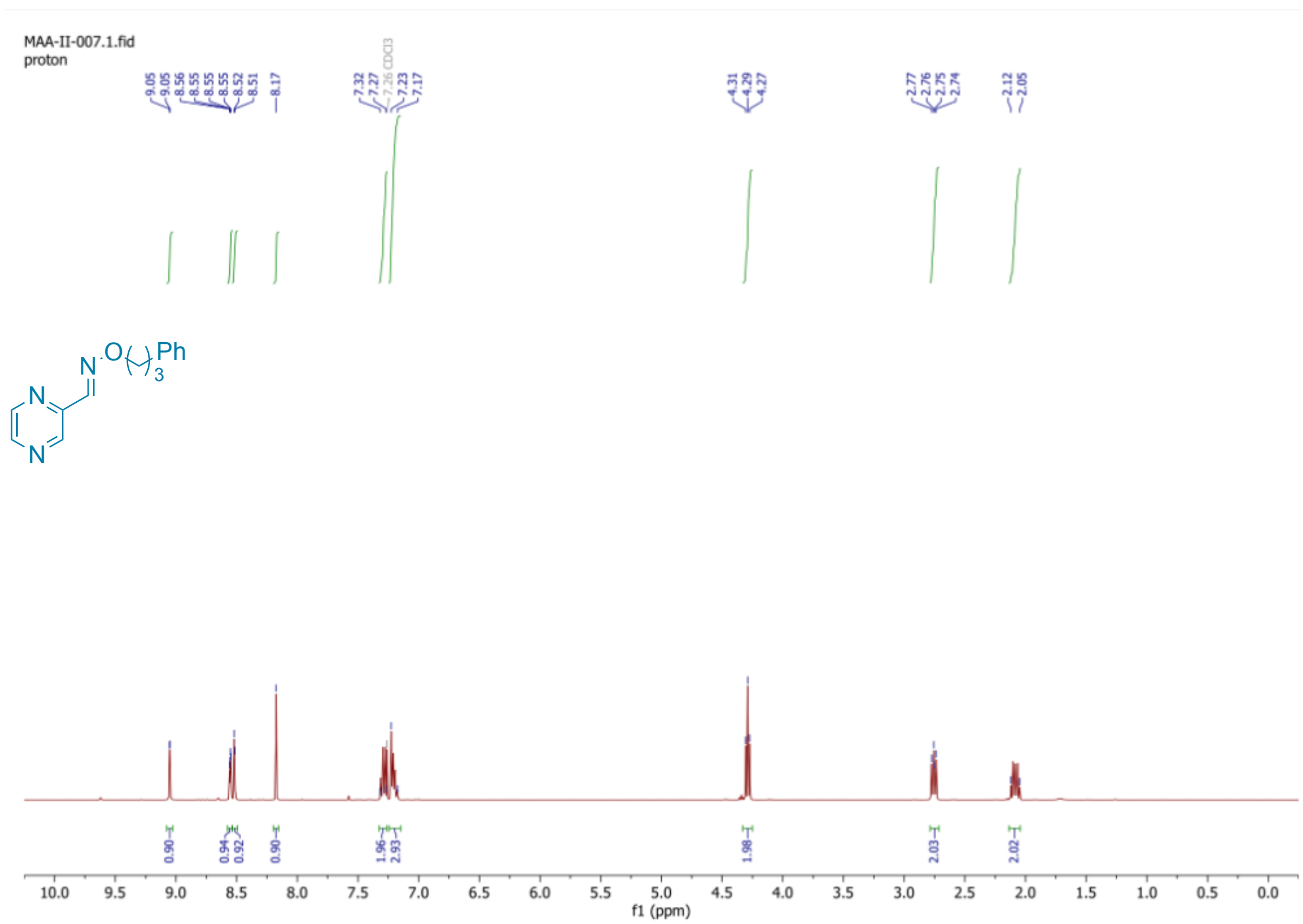

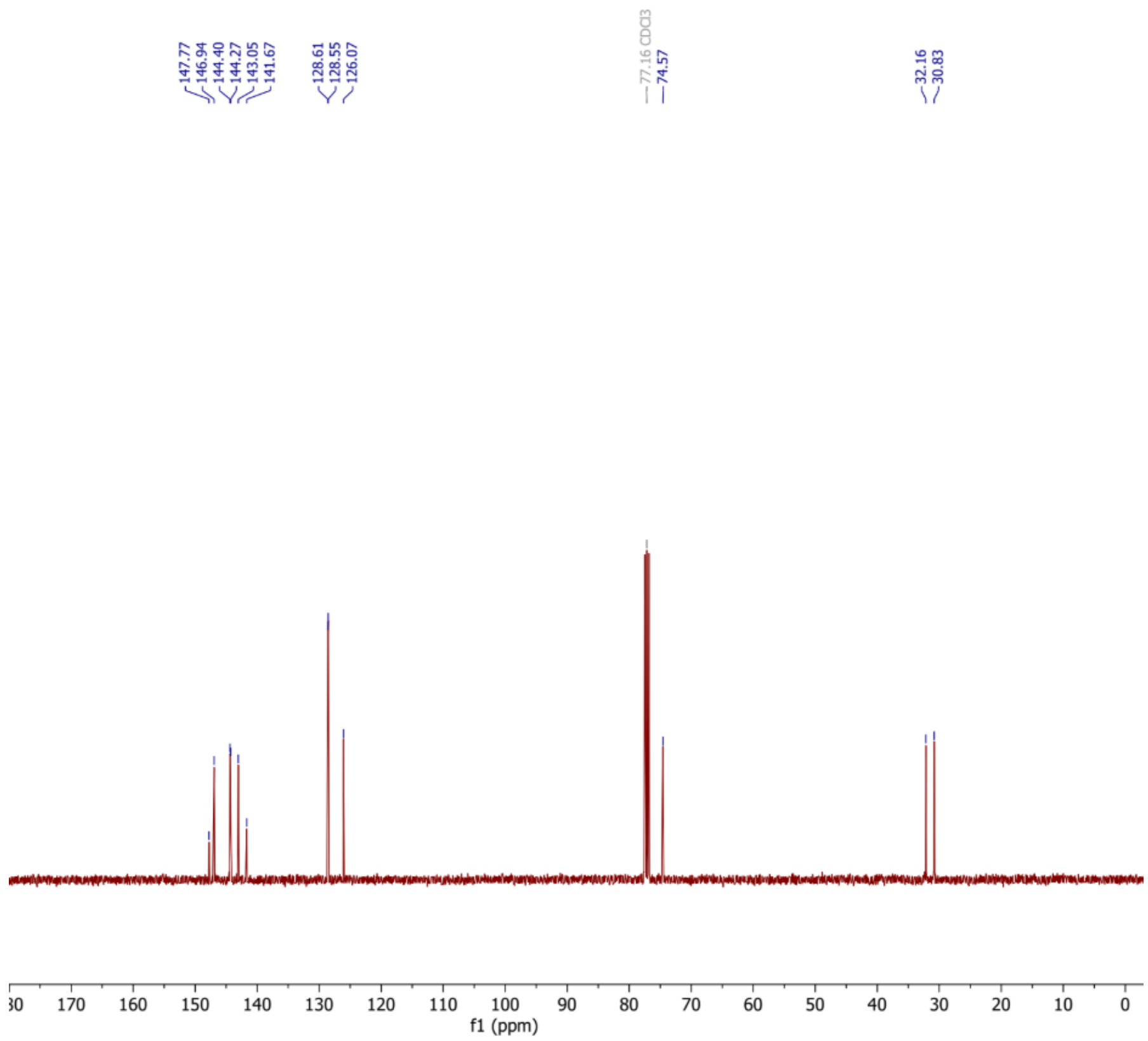

2,2,2-trifluoroacetaldehyde *O*-(3-phenylpropyl) oxime

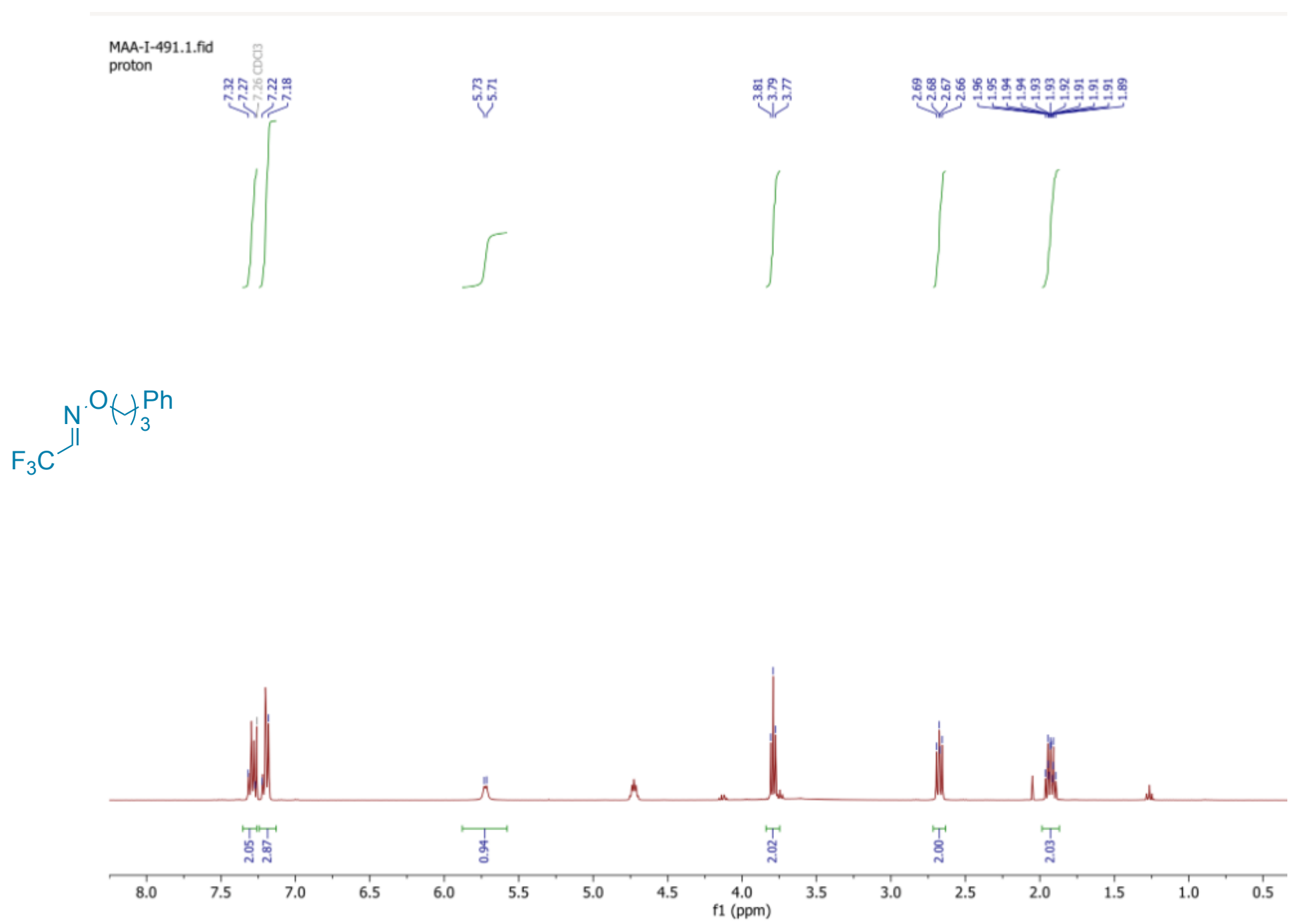

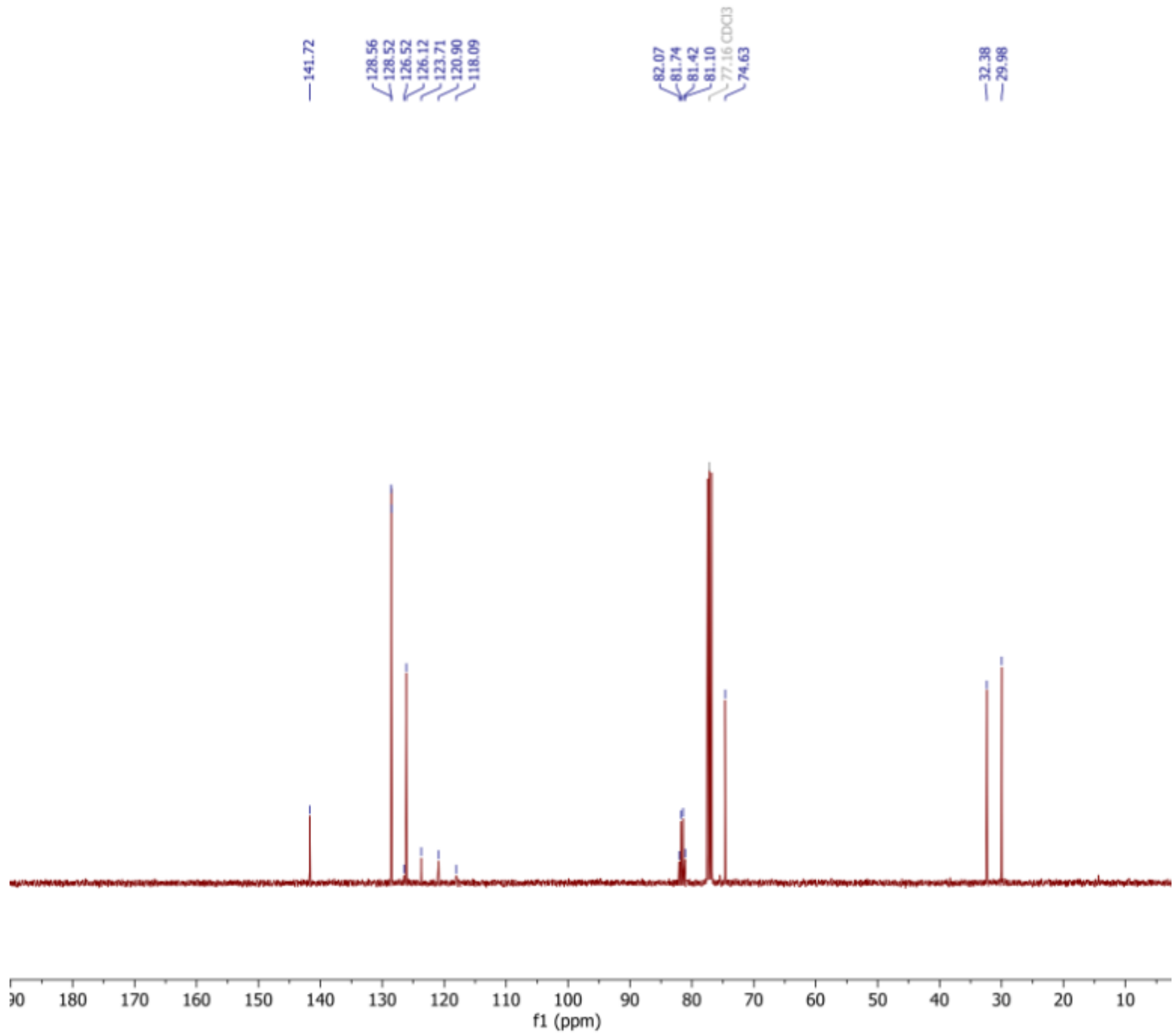
141.72
128.56
128.52
126.52
126.12
123.71
120.90
118.09
82.07
81.74
81.42
81.10
77.16 CDCl3
74.63
32.38
29.98
f1 (ppm)

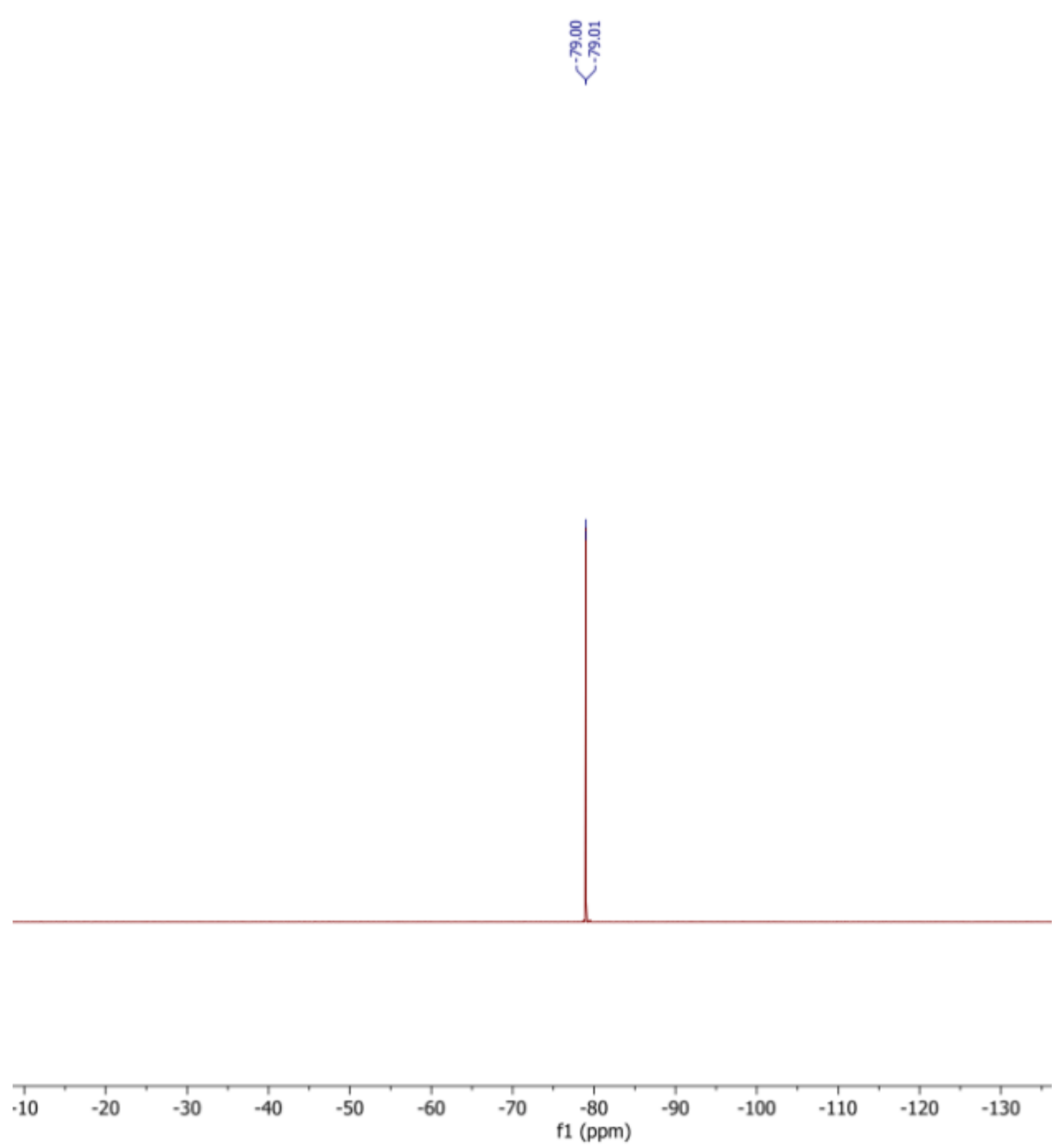
-79.00
-79.01
f1 (ppm)

Product NMR Spectra

2,2,2-trifluoro-N-(2,5,5-trimethyl-6-((3-phenylpropoxy)amino)hexan-2-yl)acetamide (1)

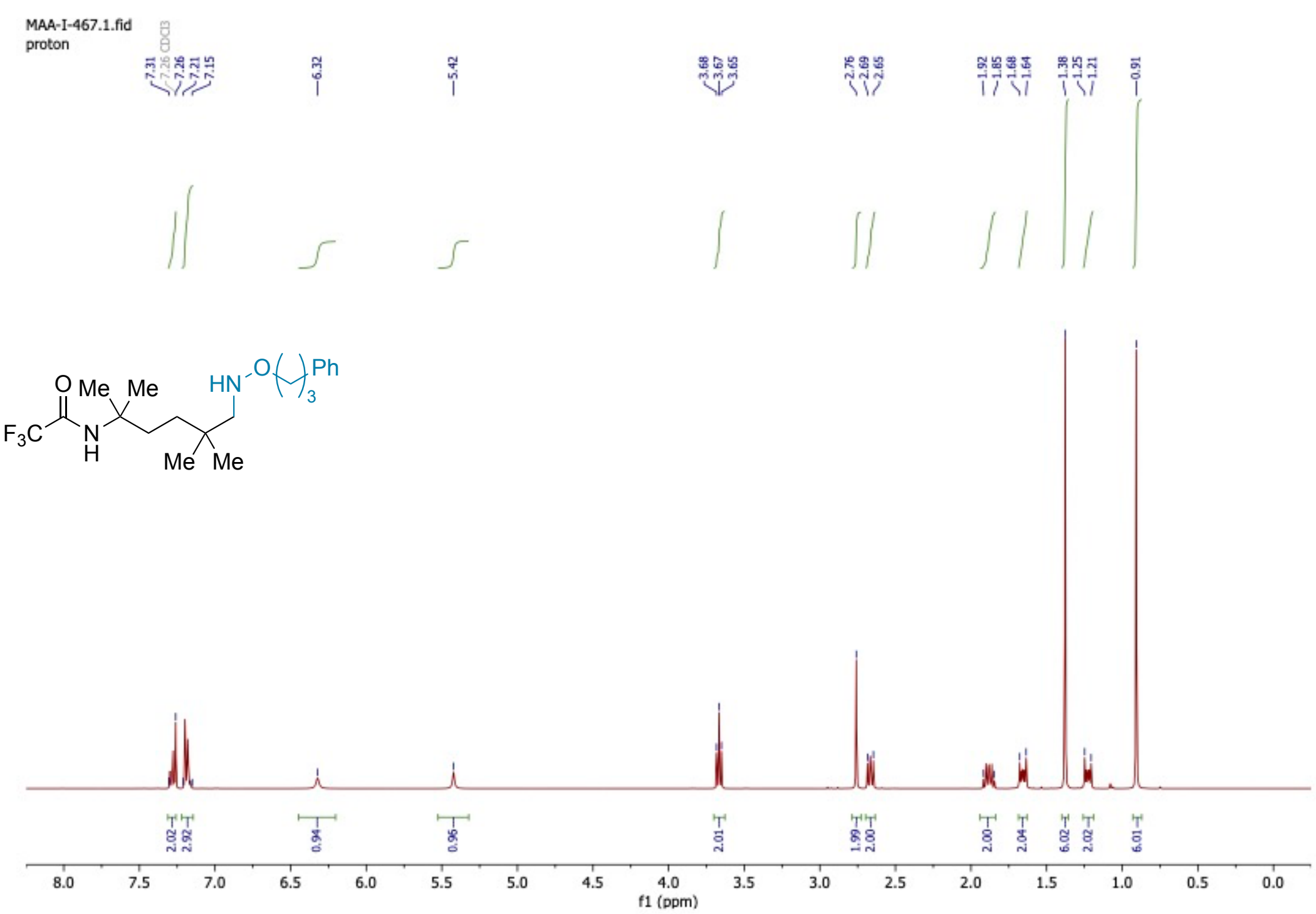

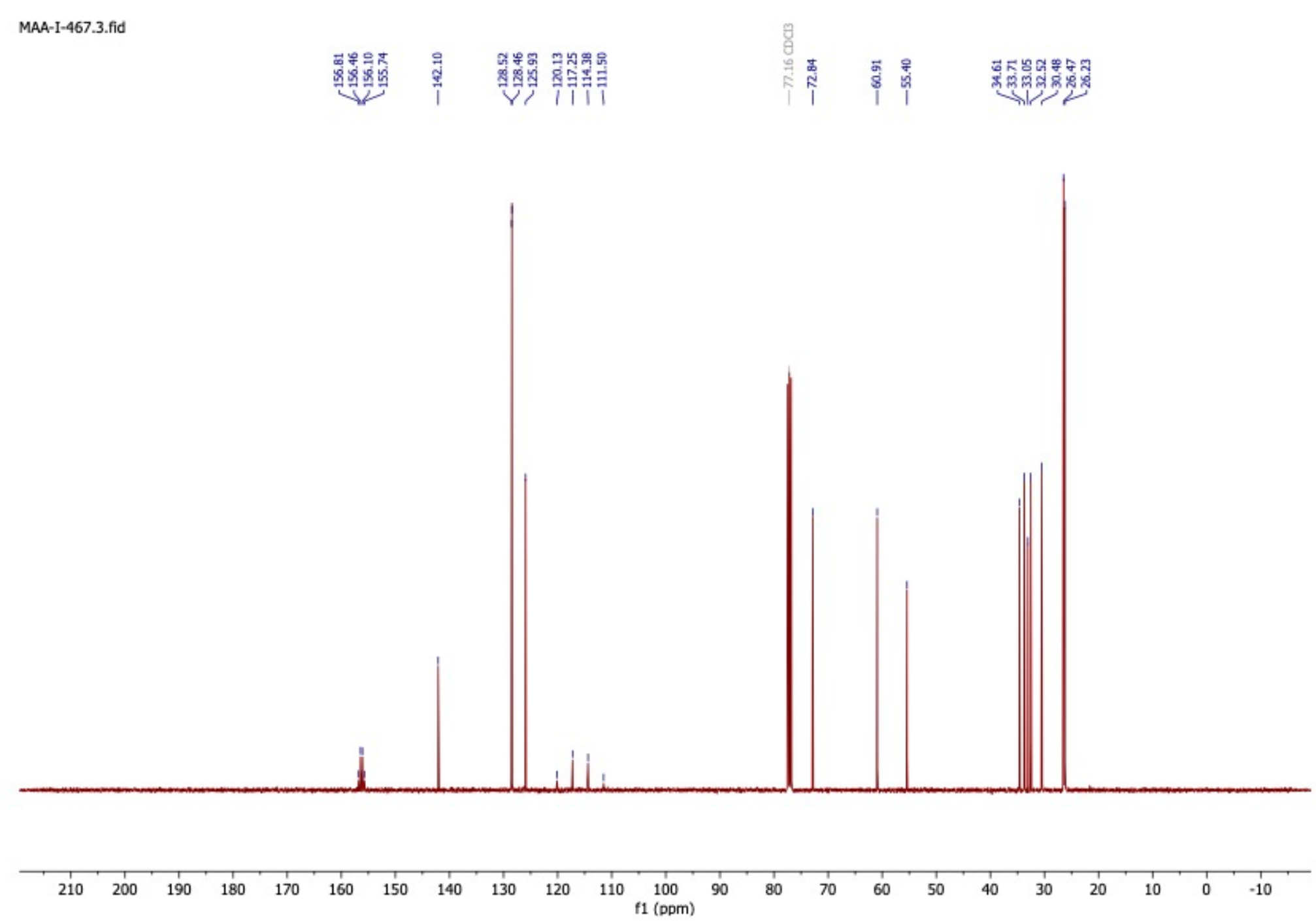

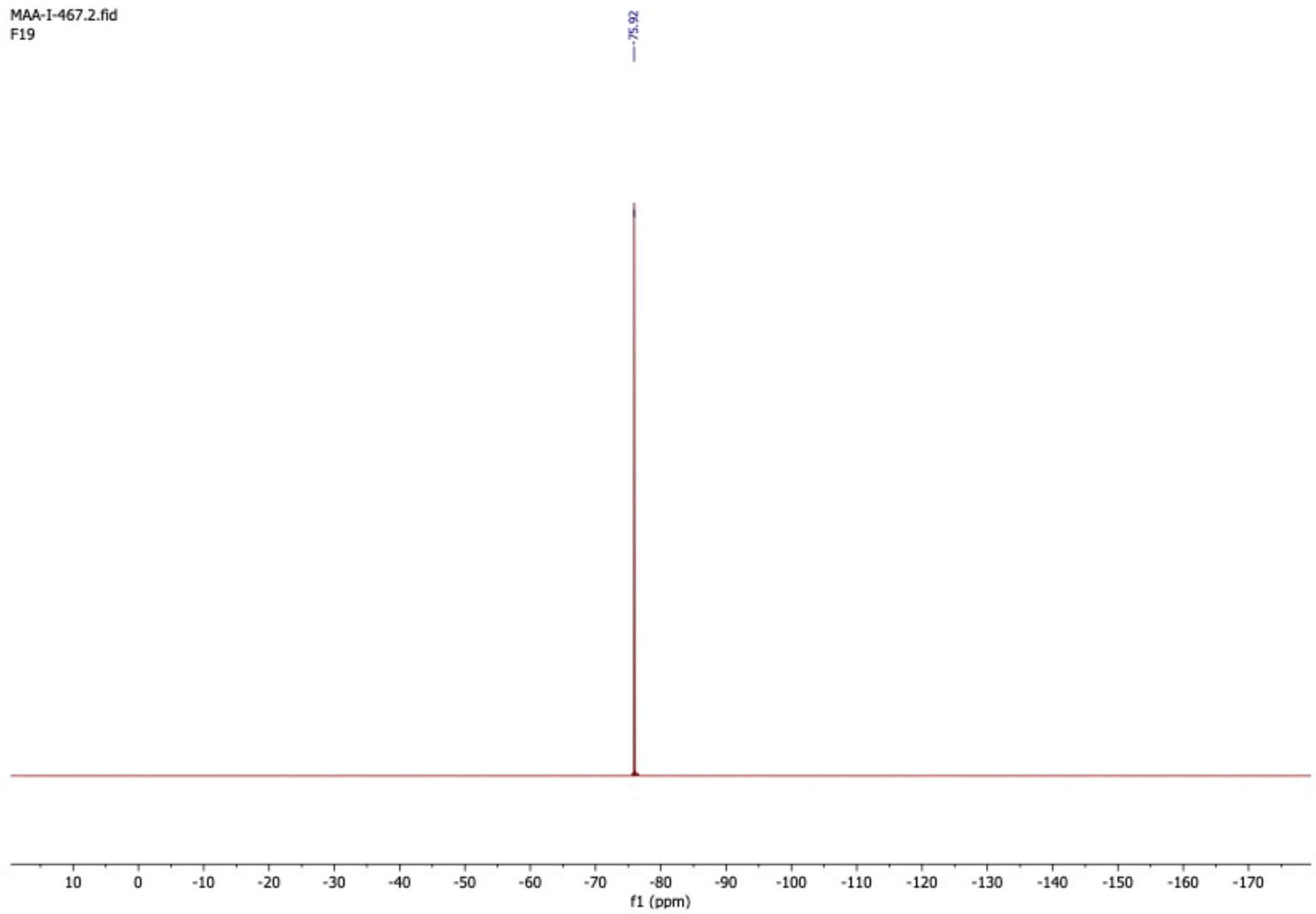

N-(5,5-dimethyl-6-((3-phenylpropoxy)amino)hexan-2-yl)-2,2,2-trifluoroacetamide (2)

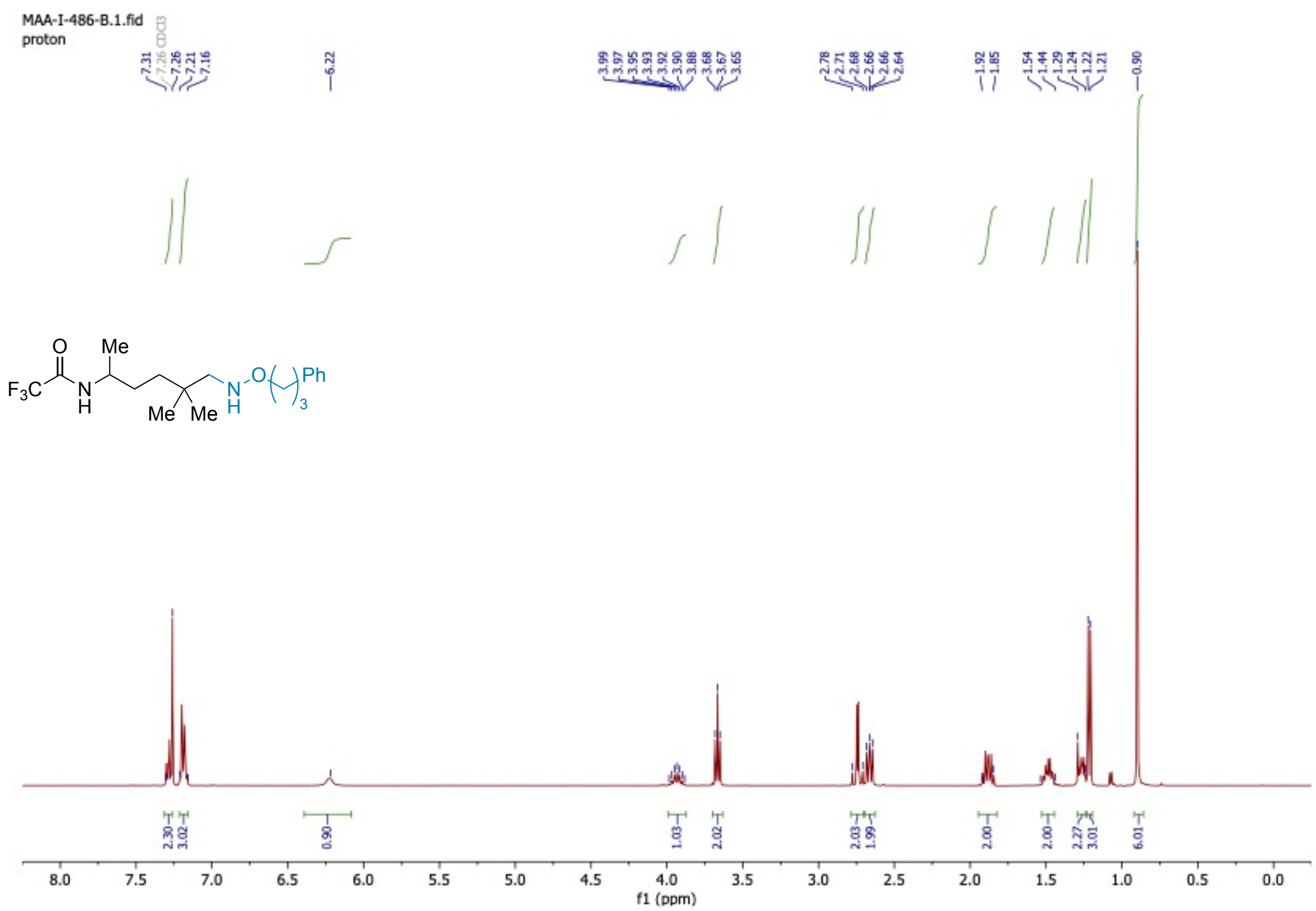

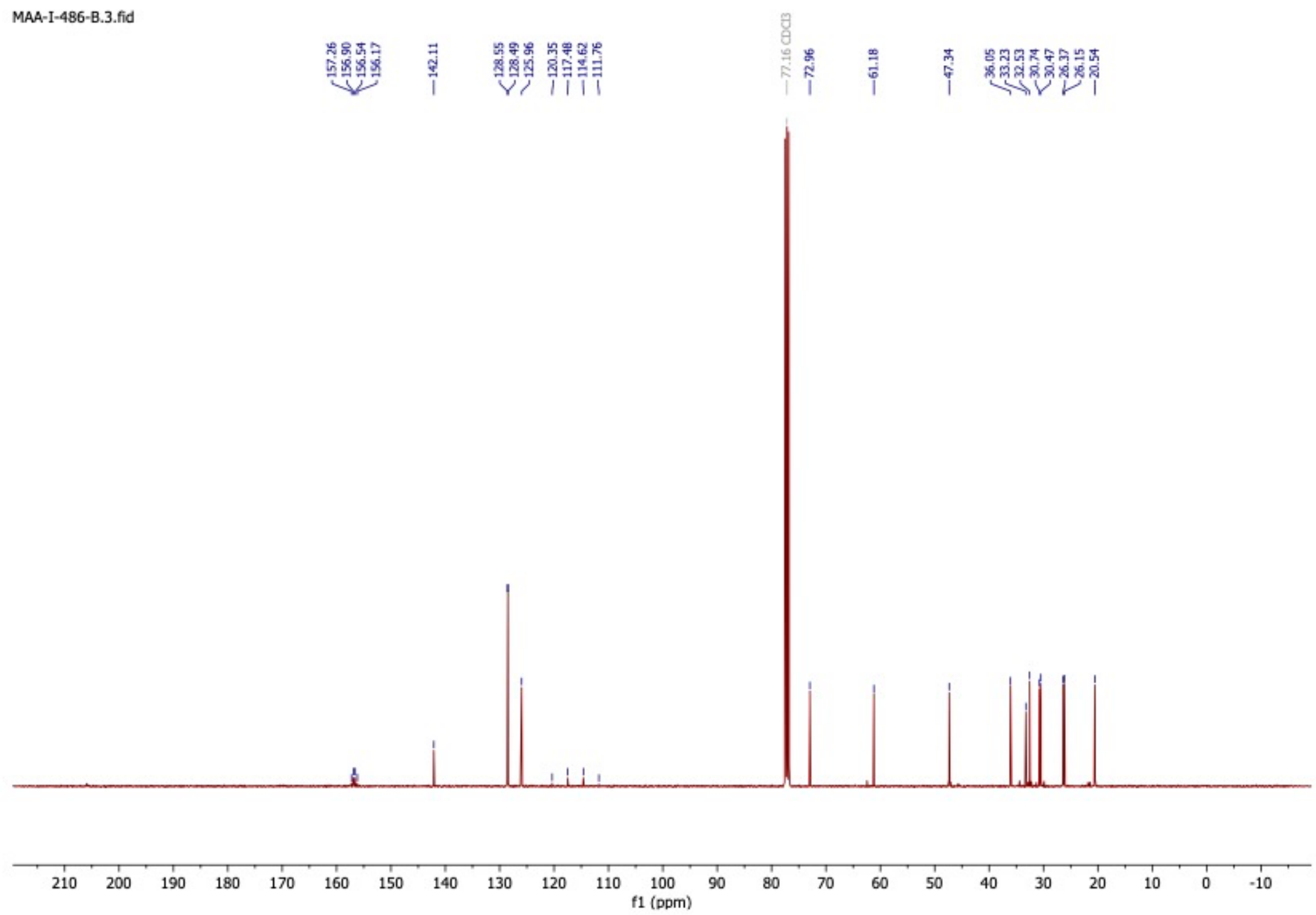
MAA-I-486-B.3.fid
157.26
156.90
156.54
156.17
142.11
128.55
128.49
125.96
120.35
117.48
114.62
111.76
77.16 CDCl3
72.96
61.18
47.34
36.05
33.23
32.53
30.74
30.47
26.37
26.15
20.54
f1 (ppm)

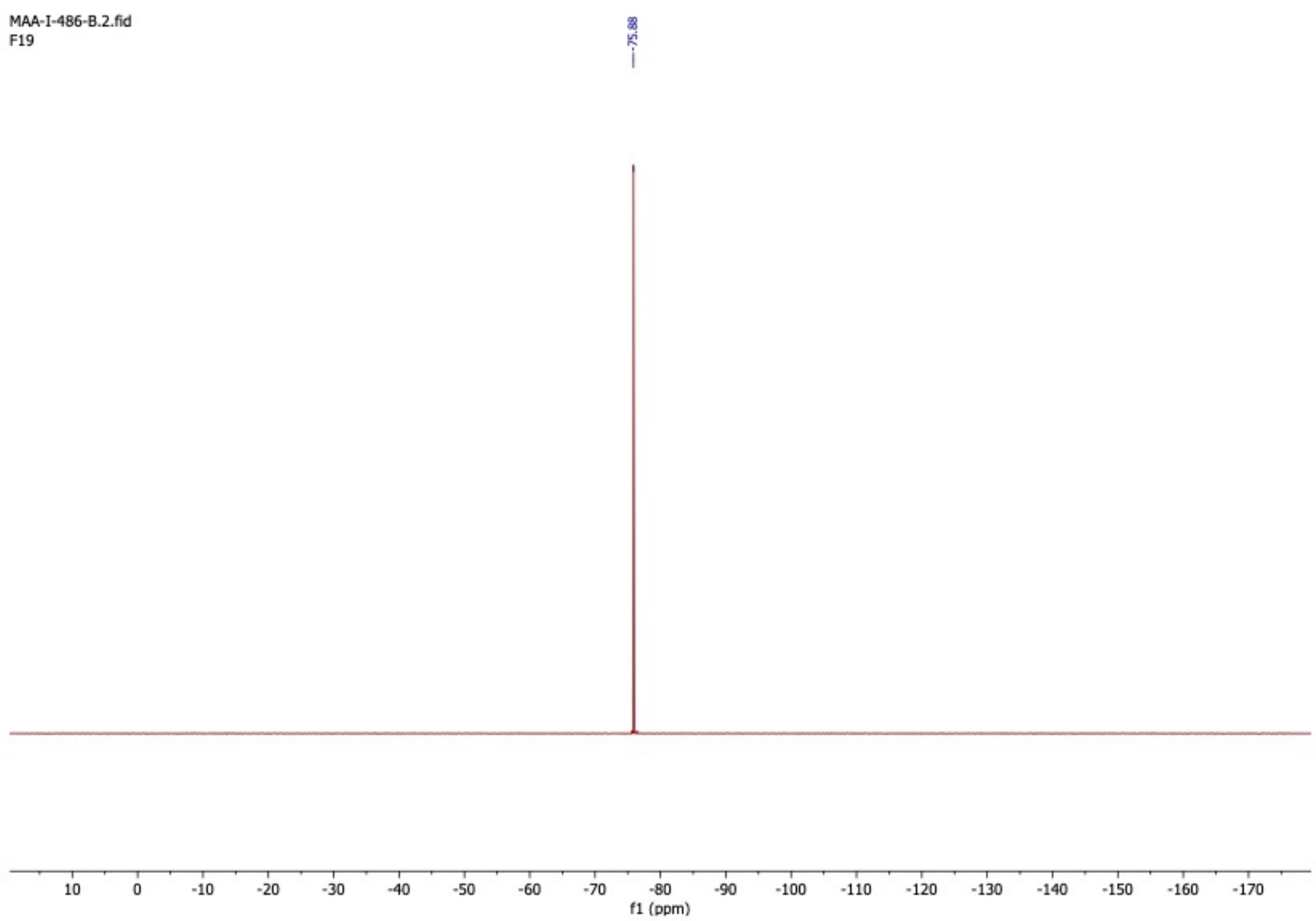
MAA-I-486-B.2.fid
F19
-75.88
f1 (ppm)

N-(4,4-dimethyl-5-((3-phenylpropoxy)amino)pentyl)-2,2,2-trifluoroacetamide (3)

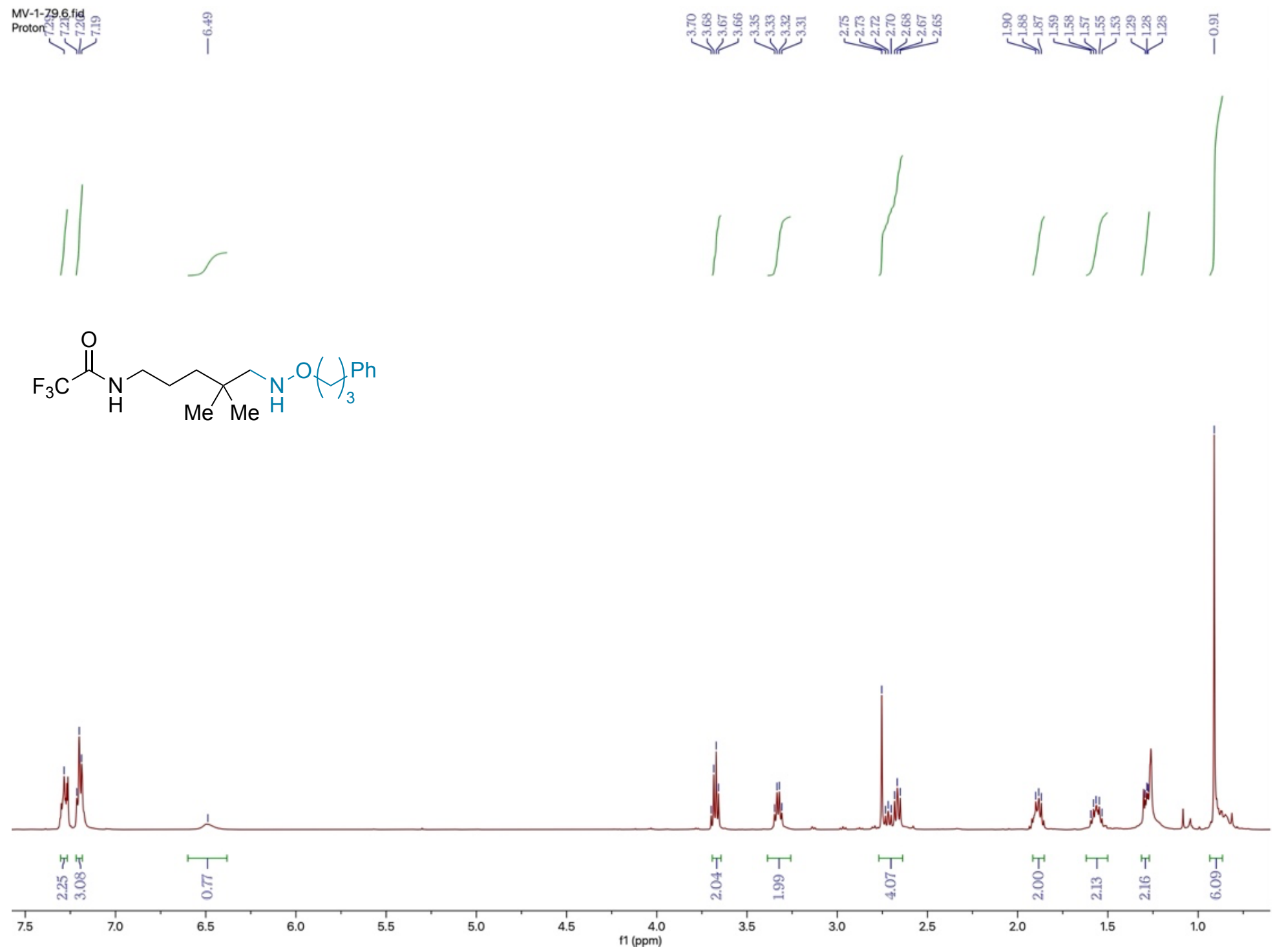

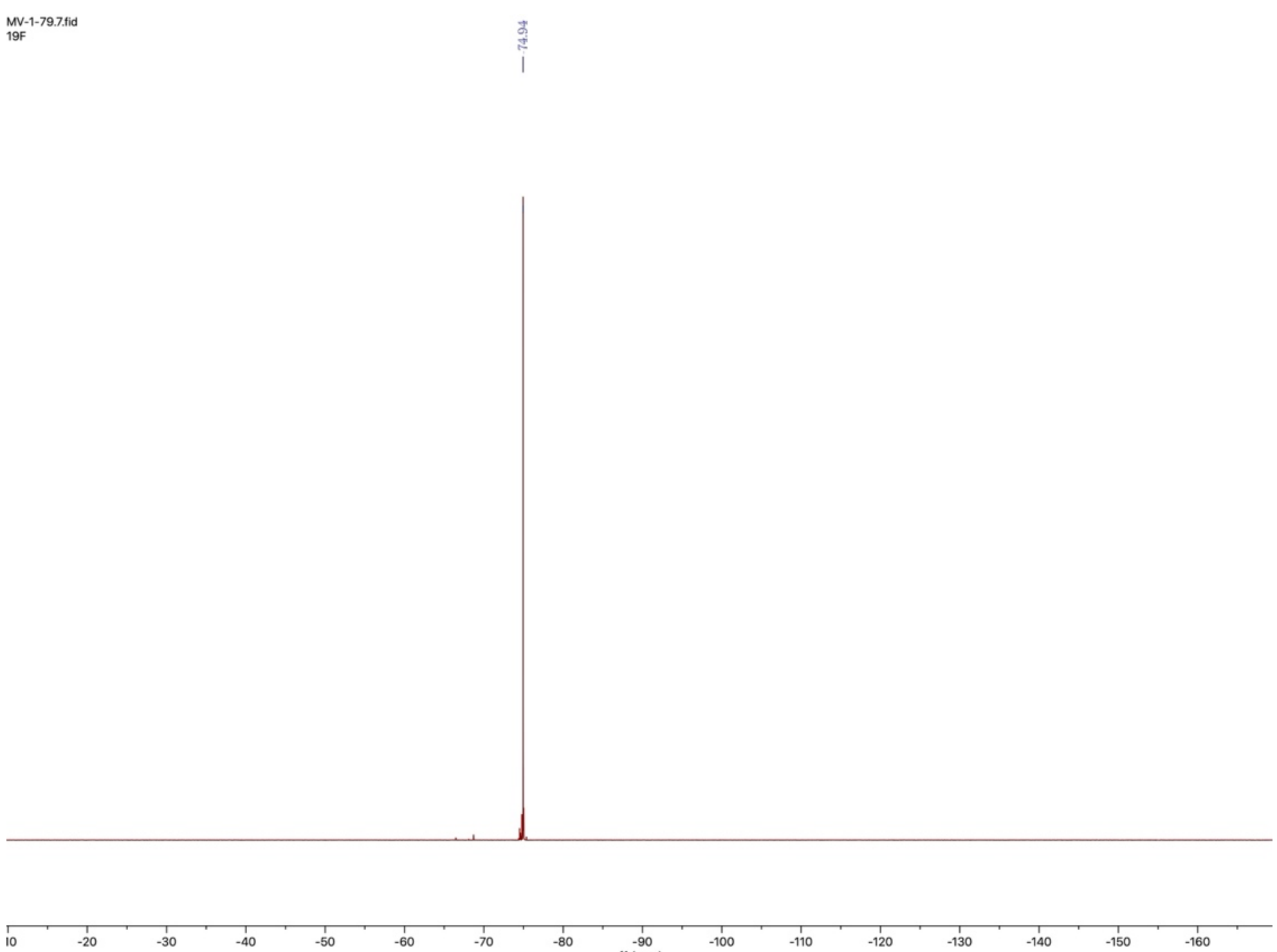

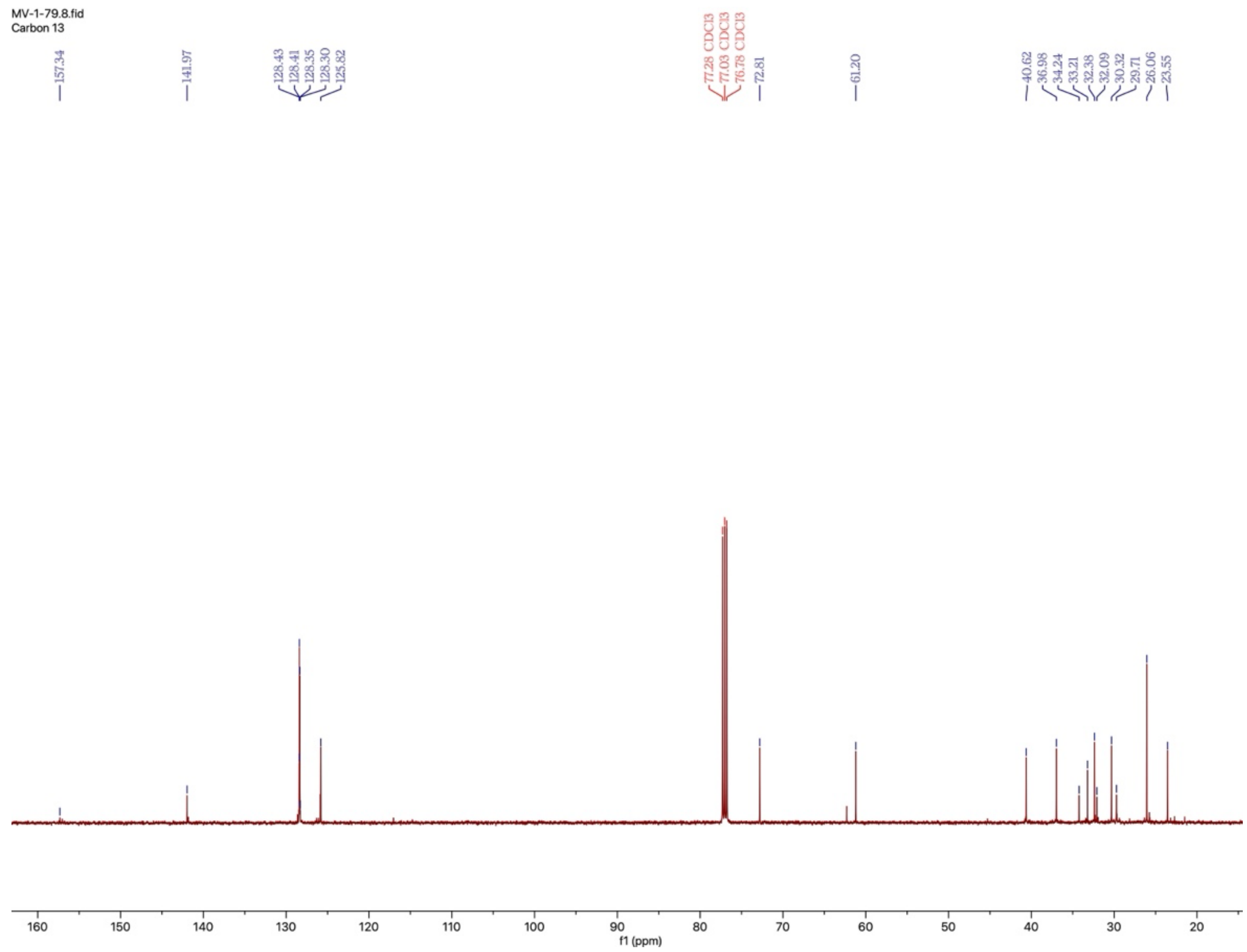

N-(4-ethyl-4-(((3-phenylpropoxy)amino)methyl)octyl)-2,2,2-trifluoroacetamide (4)

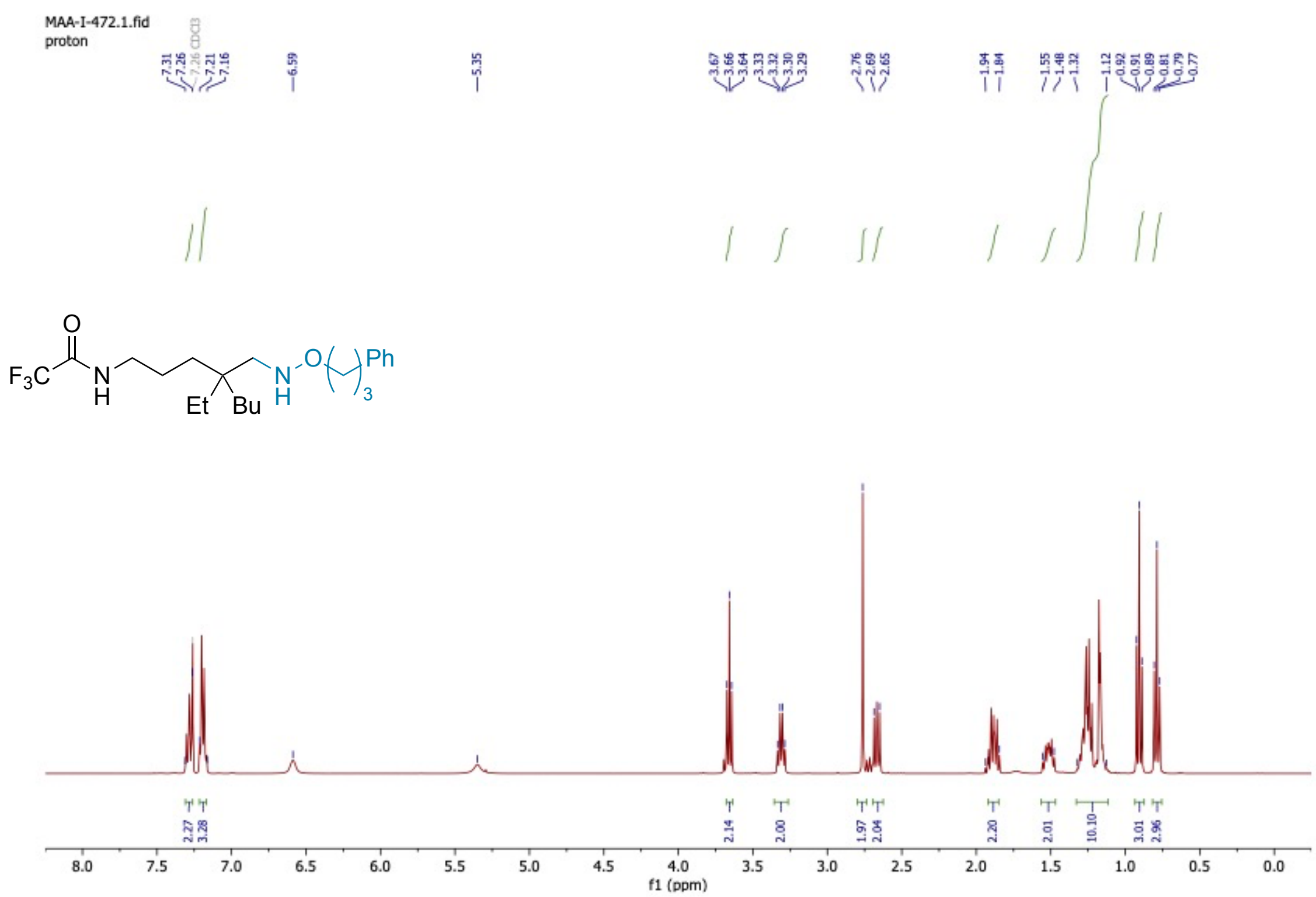

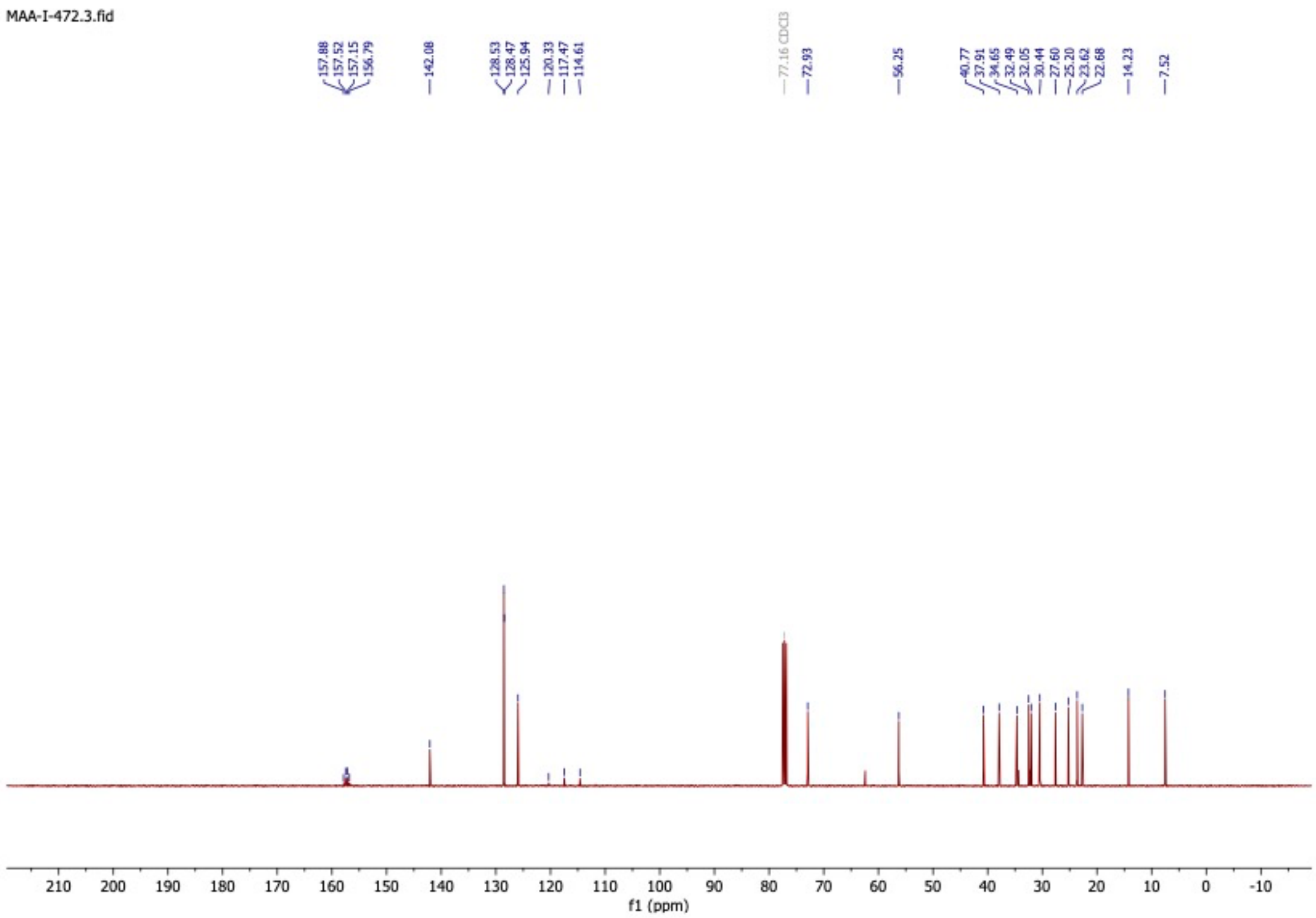
MAA-I-472.3.fid
157.88
157.52
157.15
156.79
142.08
128.53
128.47
125.94
120.33
117.47
114.61
77.16 CDCl3
72.93
56.25
40.77
37.91
34.65
32.49
32.05
30.44
27.60
25.20
23.62
22.68
14.23
7.52
f1 (ppm)

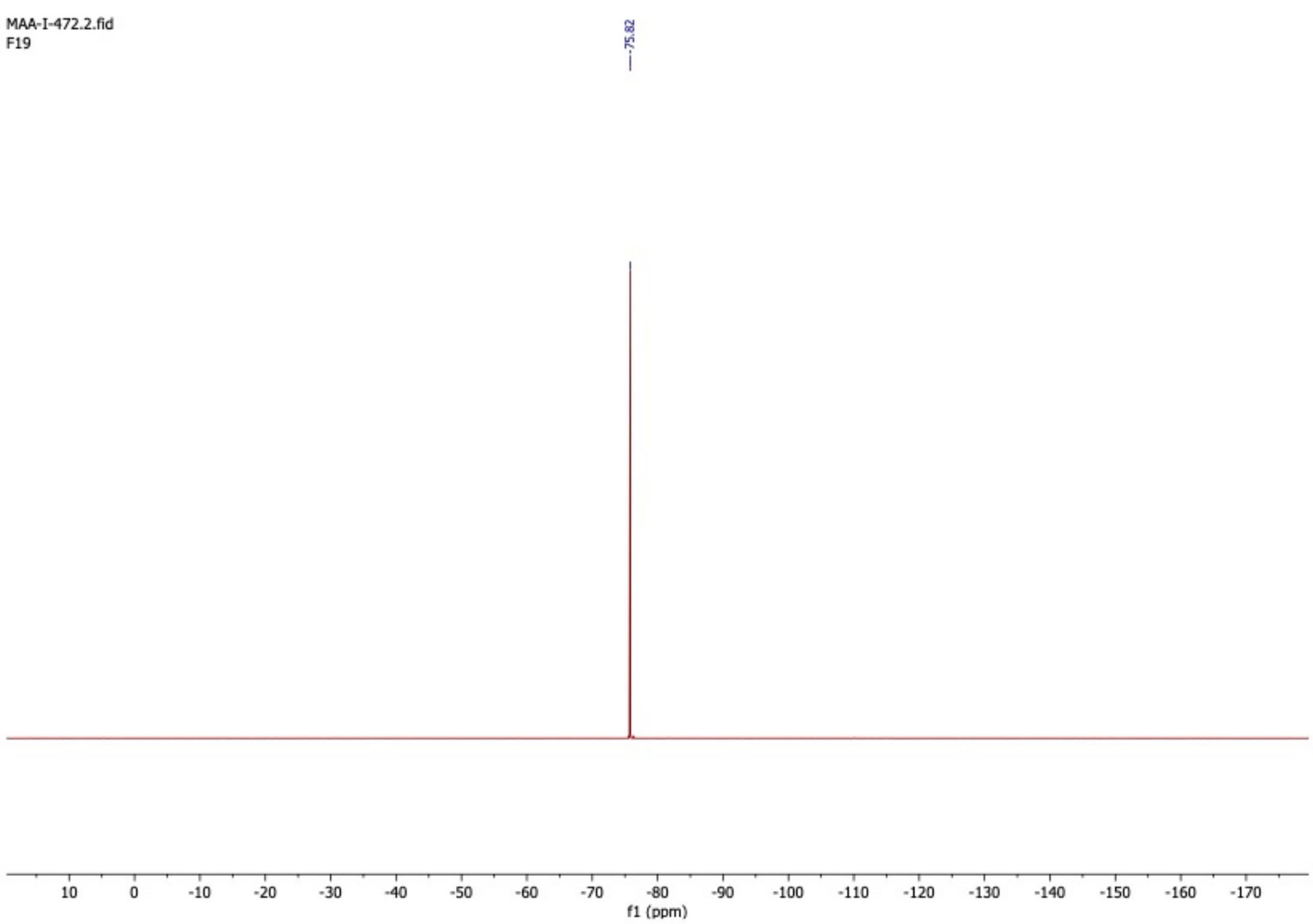
MAA-I-472.2.fid
F19
-75.82
f1 (ppm)

N-(4,8-dimethyl-4-(((3-phenylpropoxy)amino)methyl)nonyl)-2,2,2-trifluoroacetamide (5)

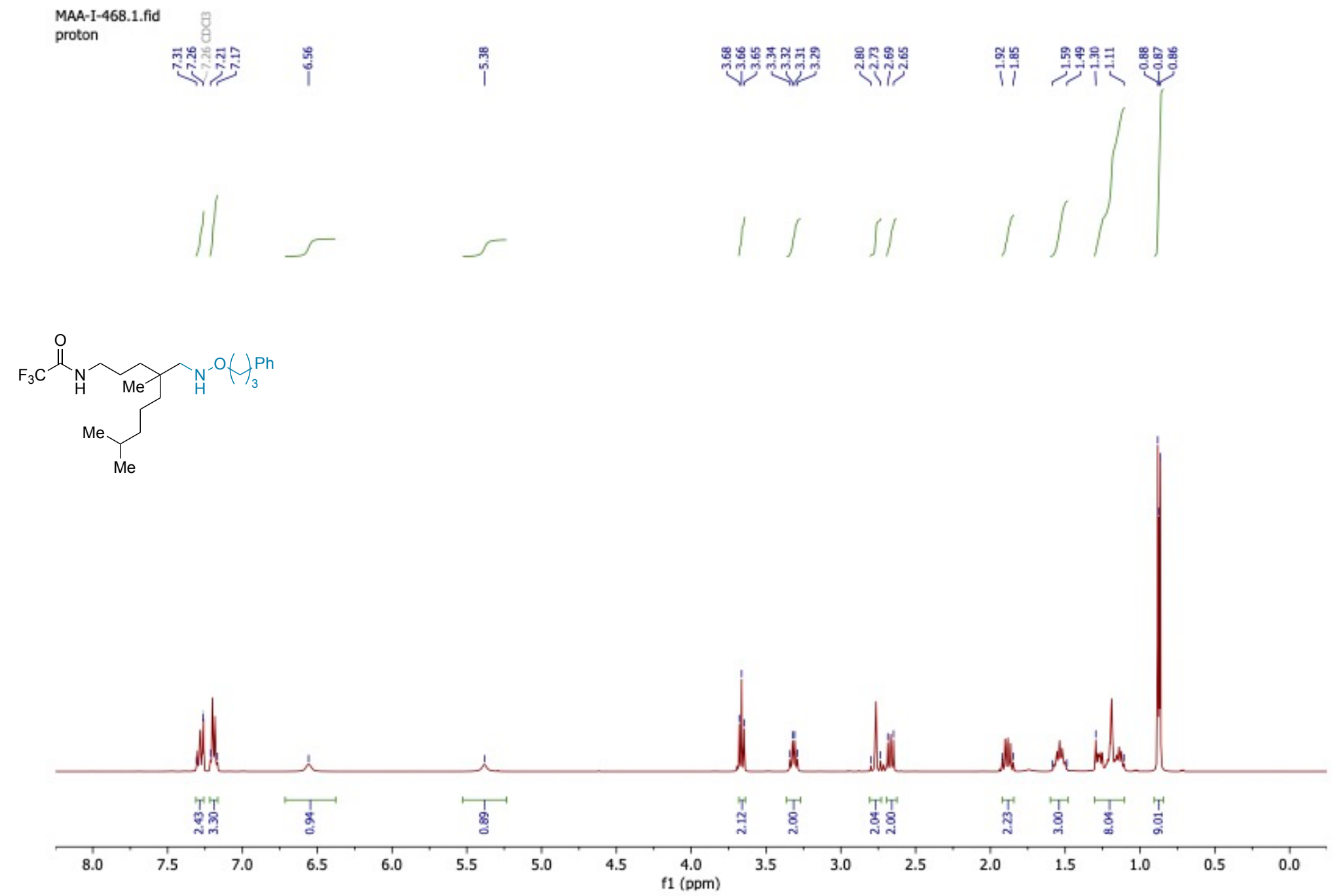

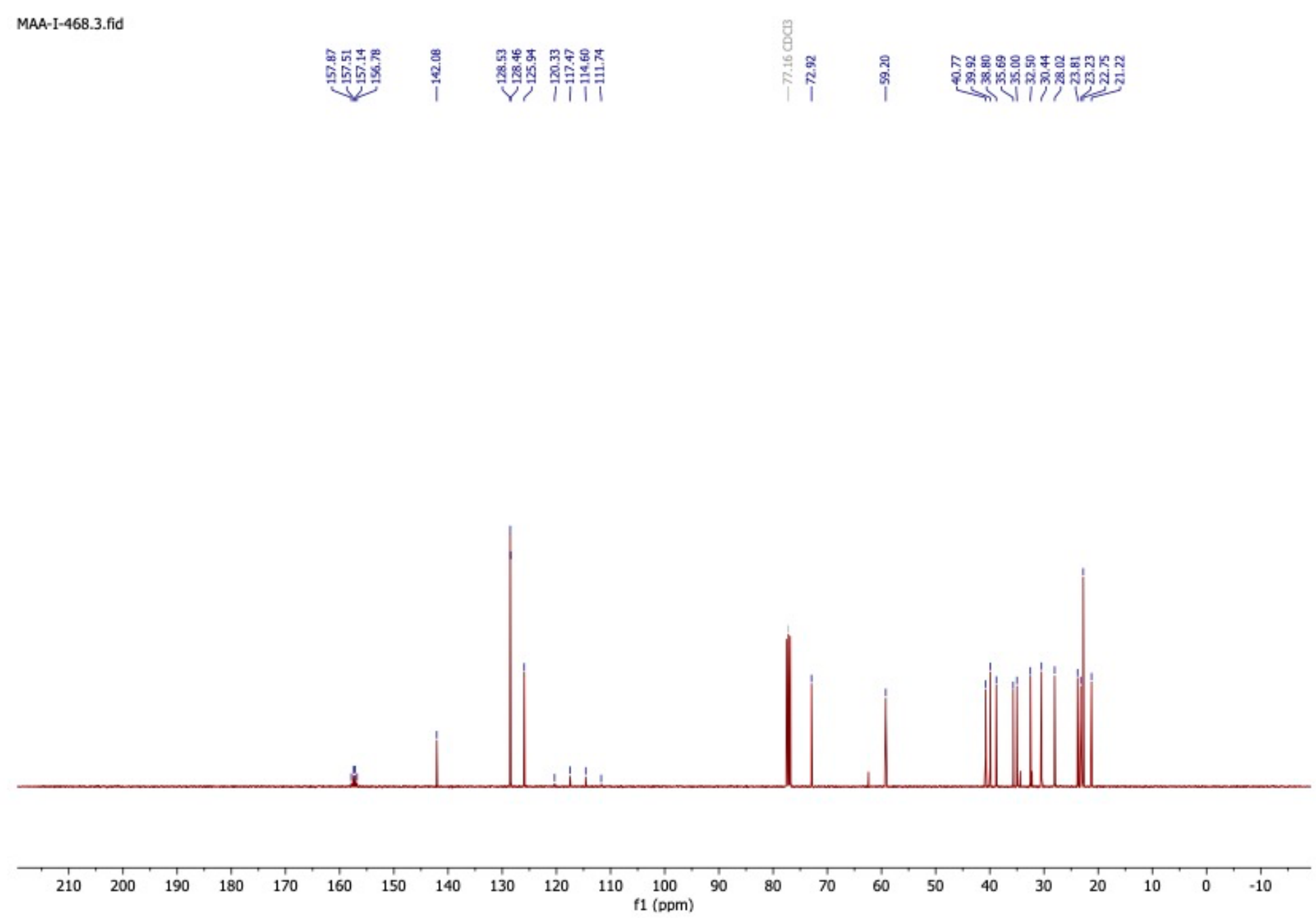

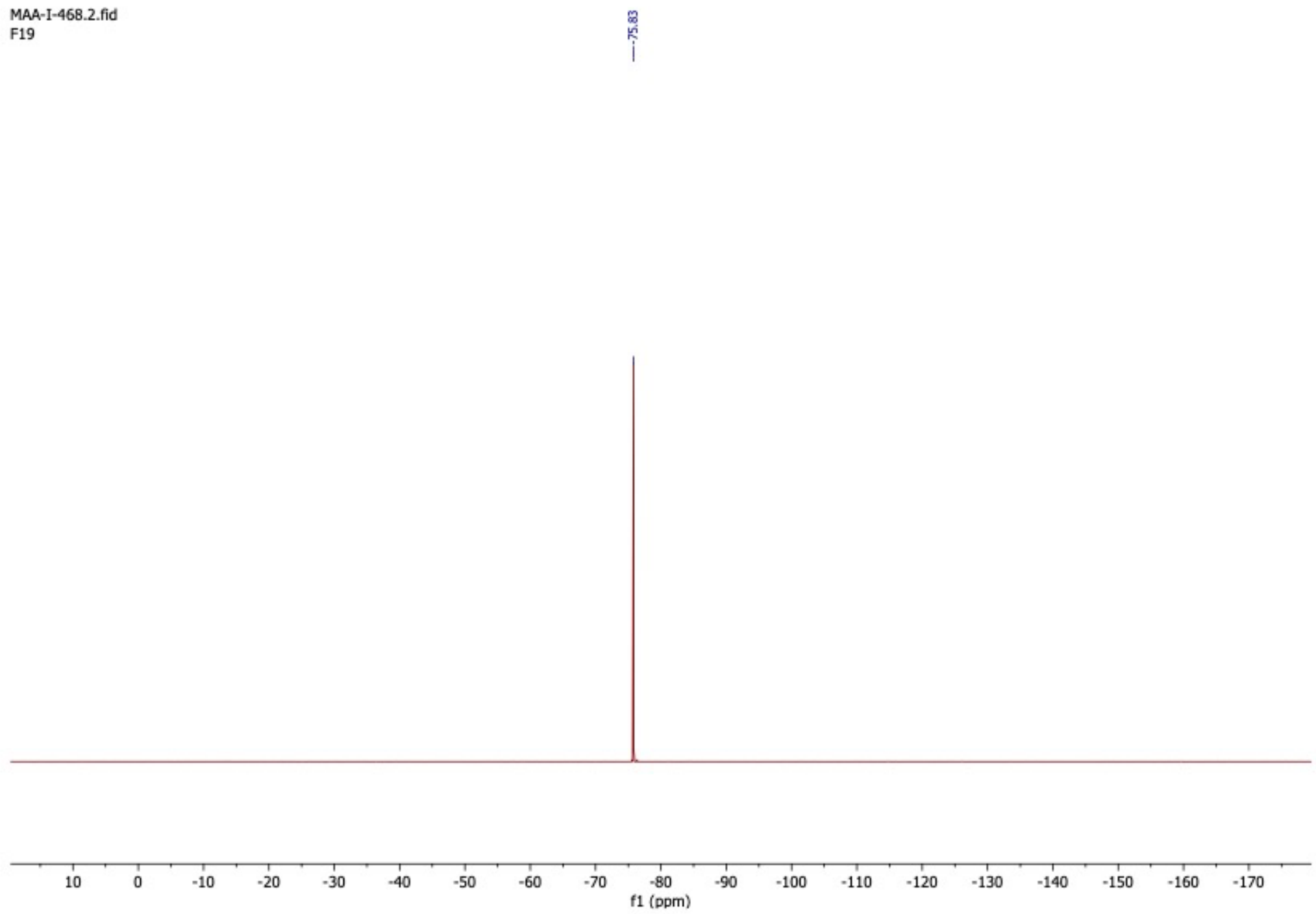

2,2,2-trifluoro-N-(3-(1-(((3-phenylpropoxy)amino)methyl)cyclopentyl)propyl)acetamide (6)

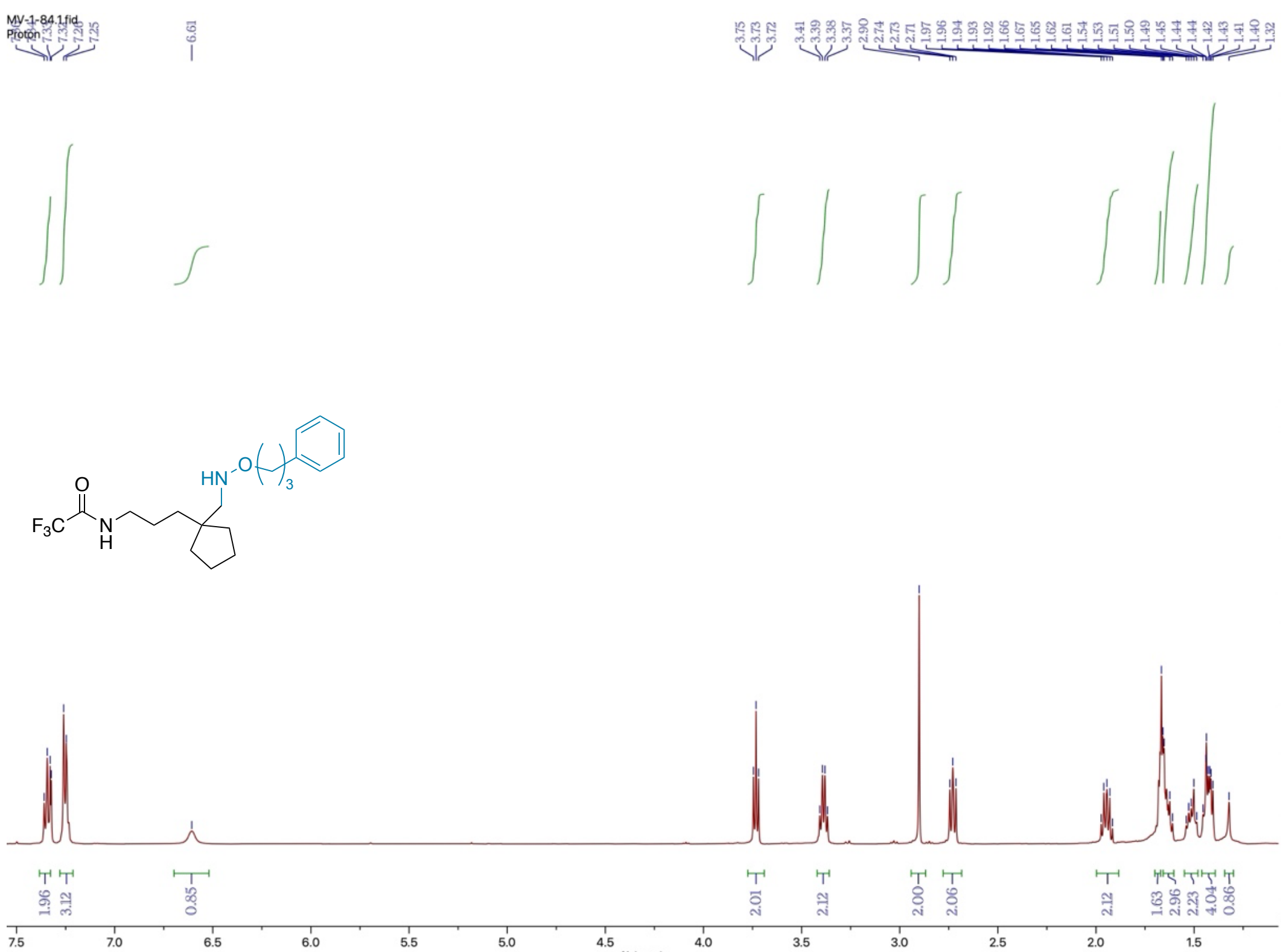

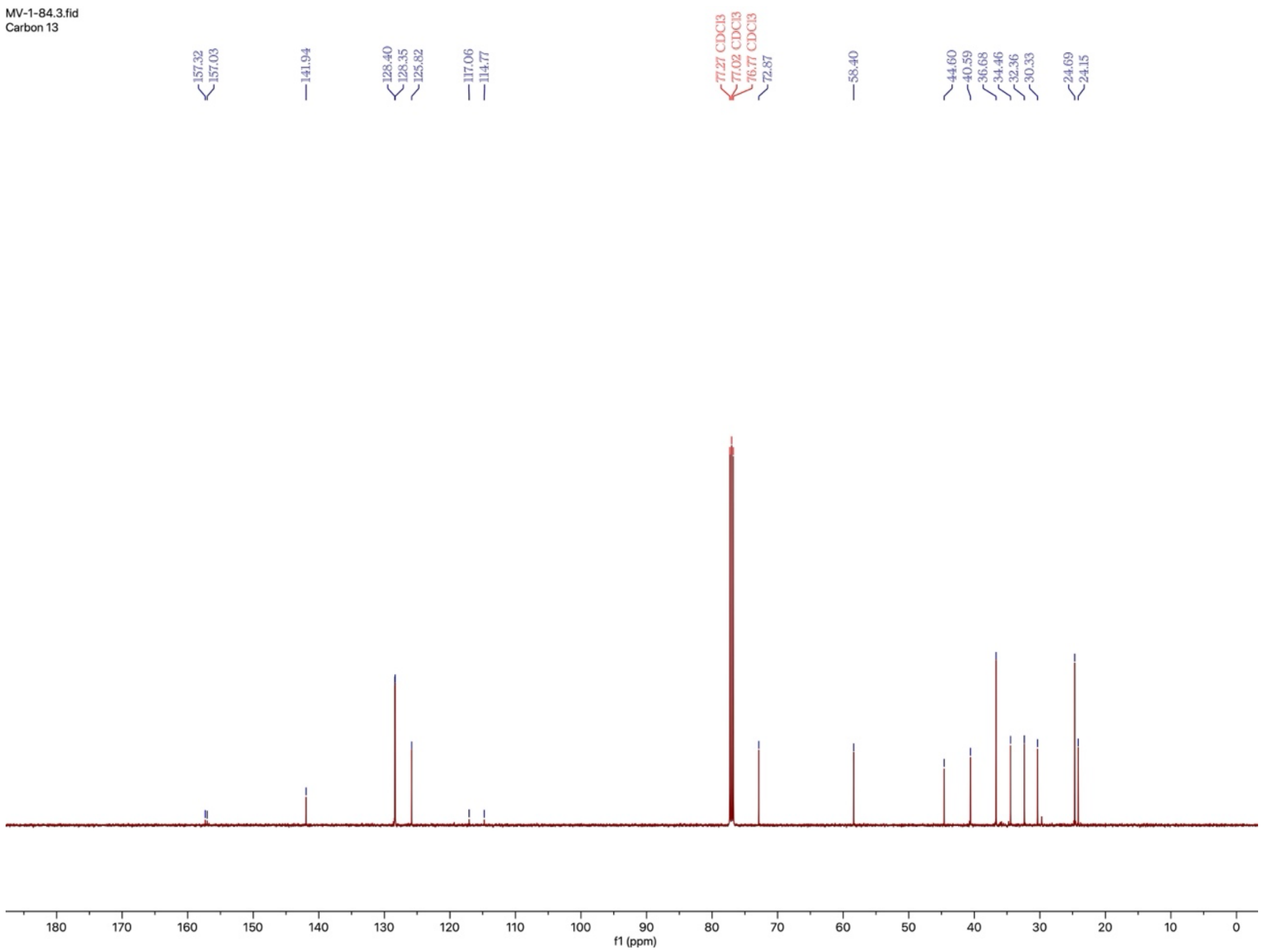
MV-1-84.3.fid
Carbon 13
157.32
157.03
141.94
128.40
128.35
125.82
117.06
114.77
77.27 CDCl3
77.02 CDCl3
76.77 CDCl3
72.87
58.40
44.60
40.59
36.68
34.46
32.36
30.33
24.69
24.15
f1 (ppm)

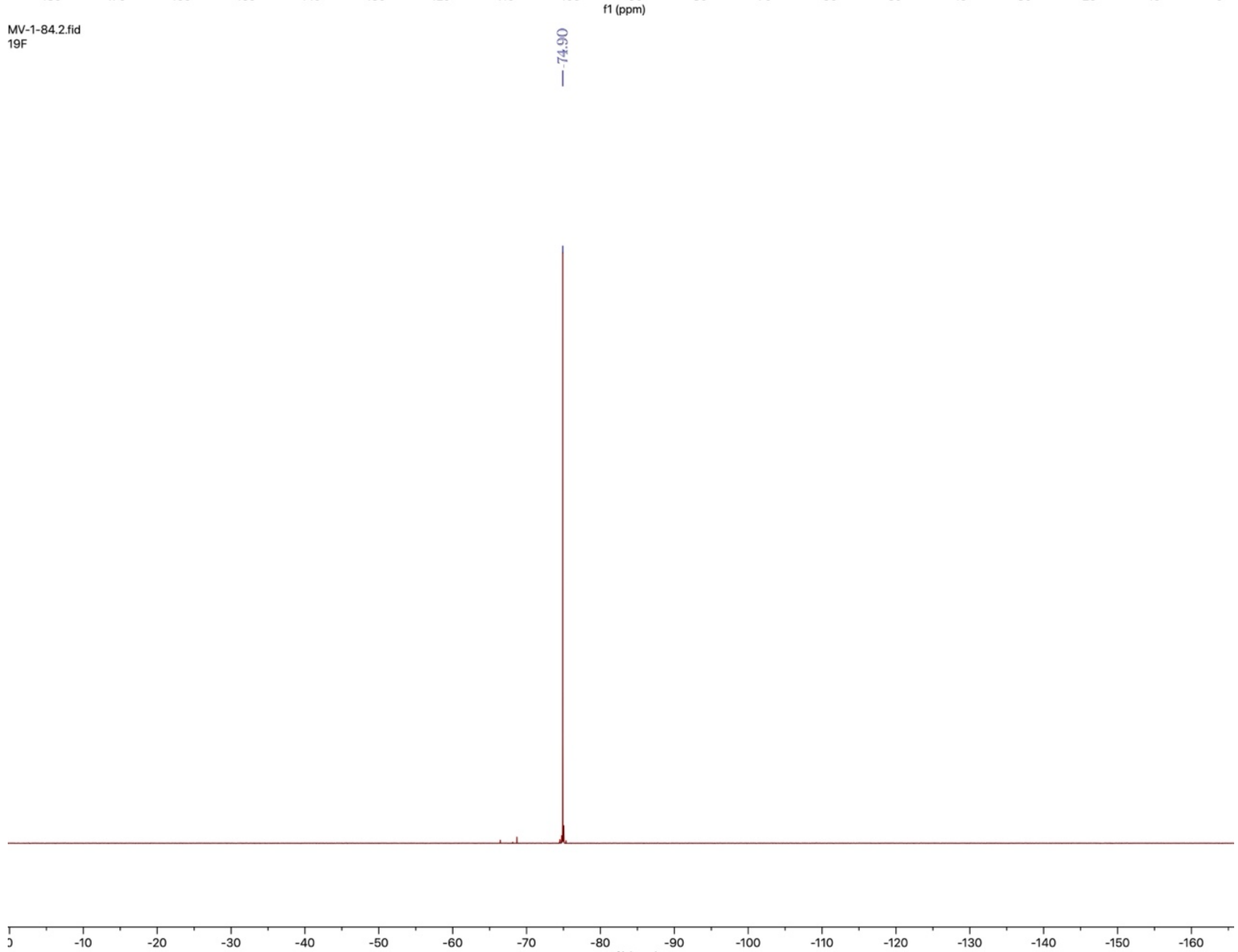
MV-1-84.2.fid
19F
-74.90
f1 (ppm)

N-(5-((tert-butyldimethylsilyl)oxy)-4-methyl-4-(((3-phenylpropoxy)amino)methyl)pentyl)-2,2,2-trifluoroacetamide (7)

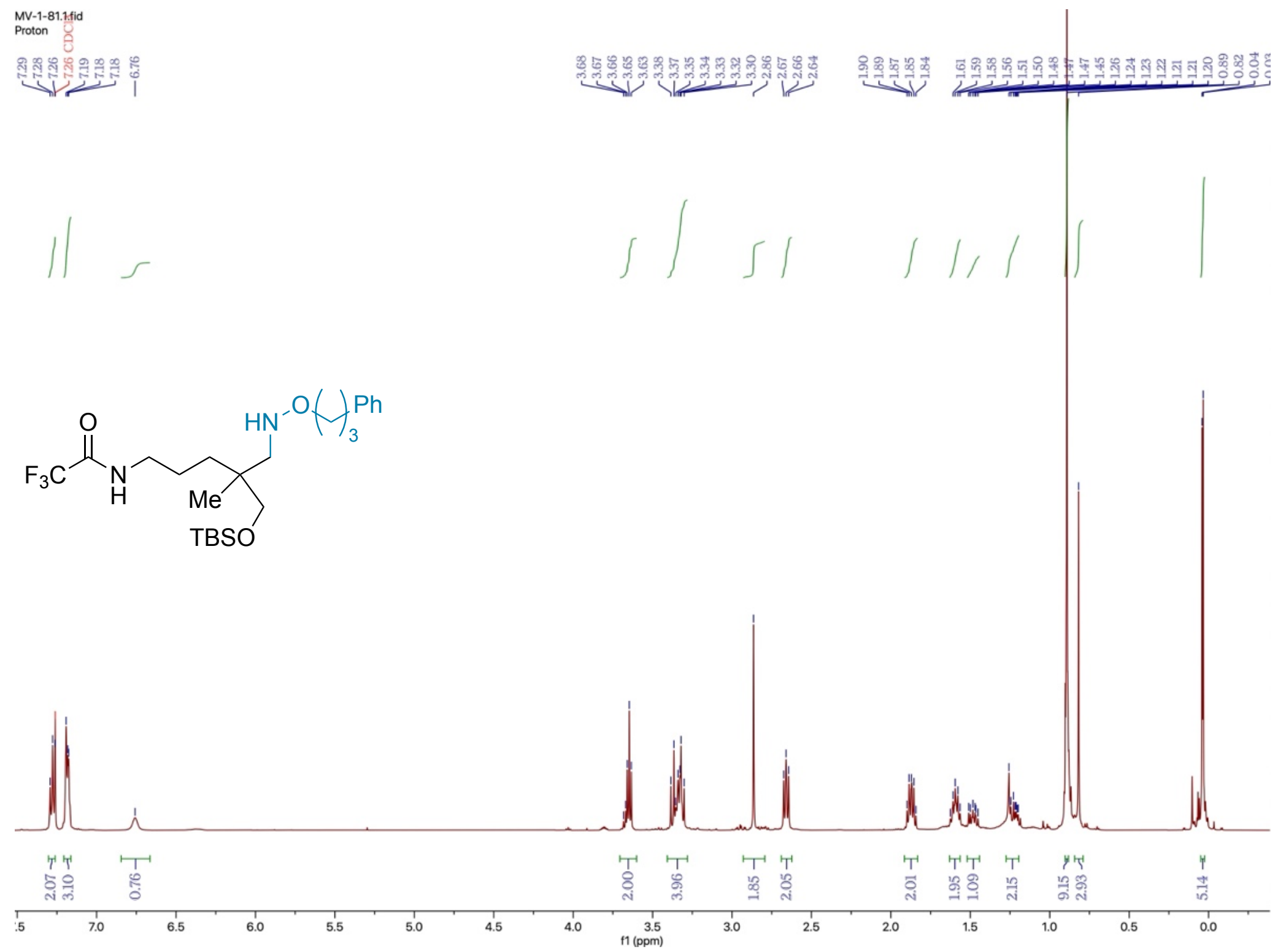

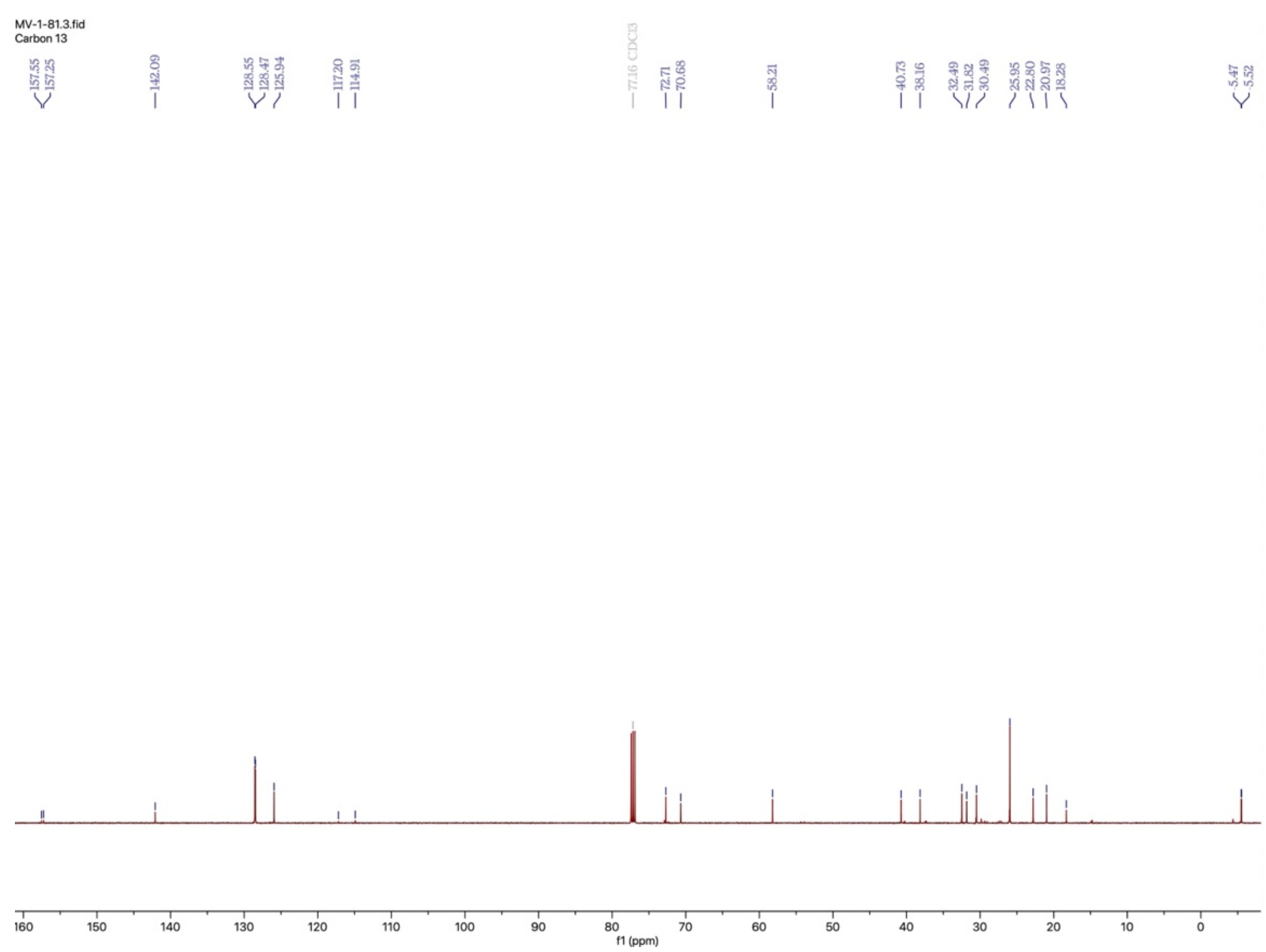

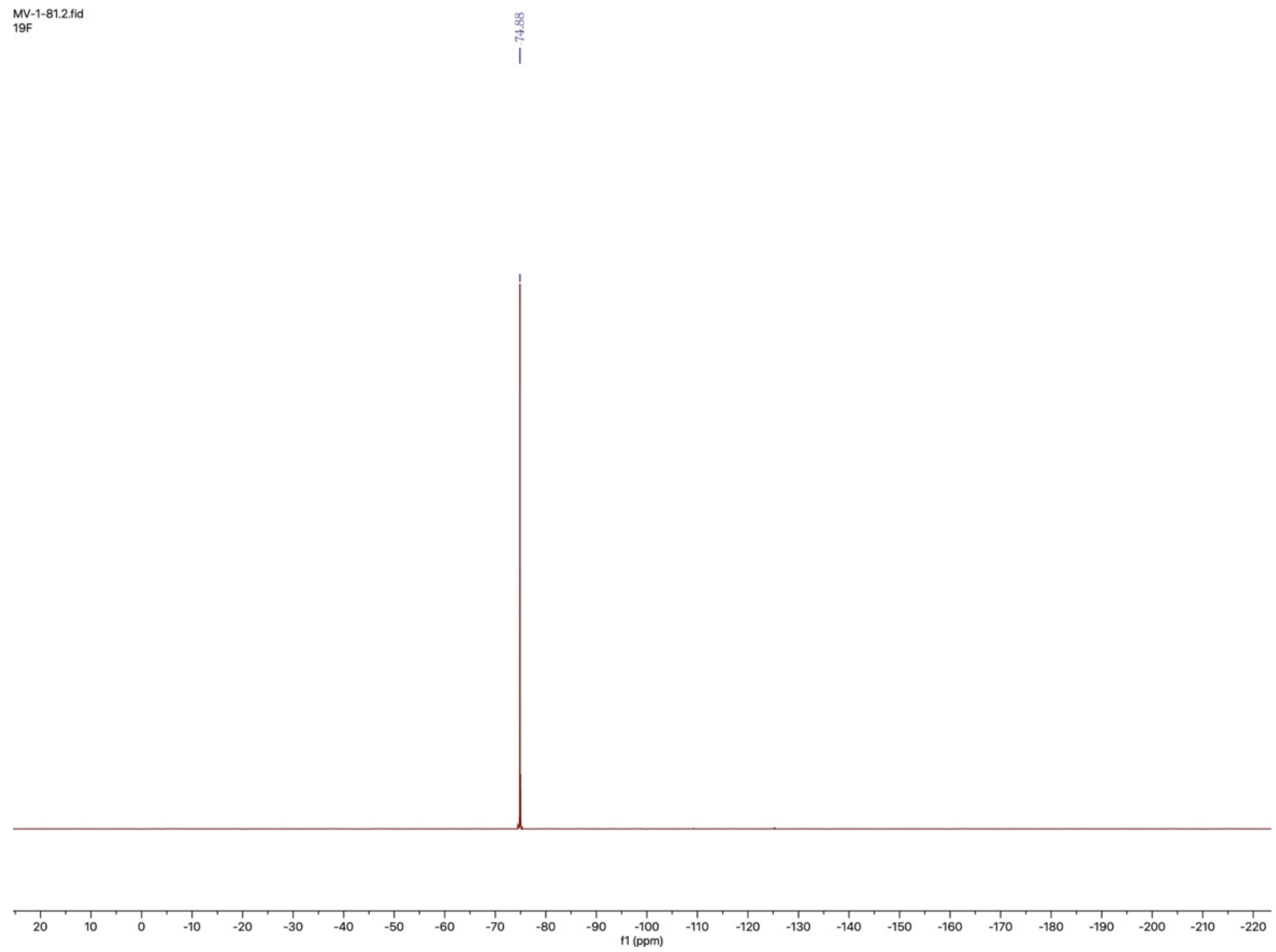

2,2,2-trifluoro-*N*-(2-((2-methyl-1-((3-phenylpropoxy)amino)propan-2-yl)oxy)ethyl)acetamide (8)

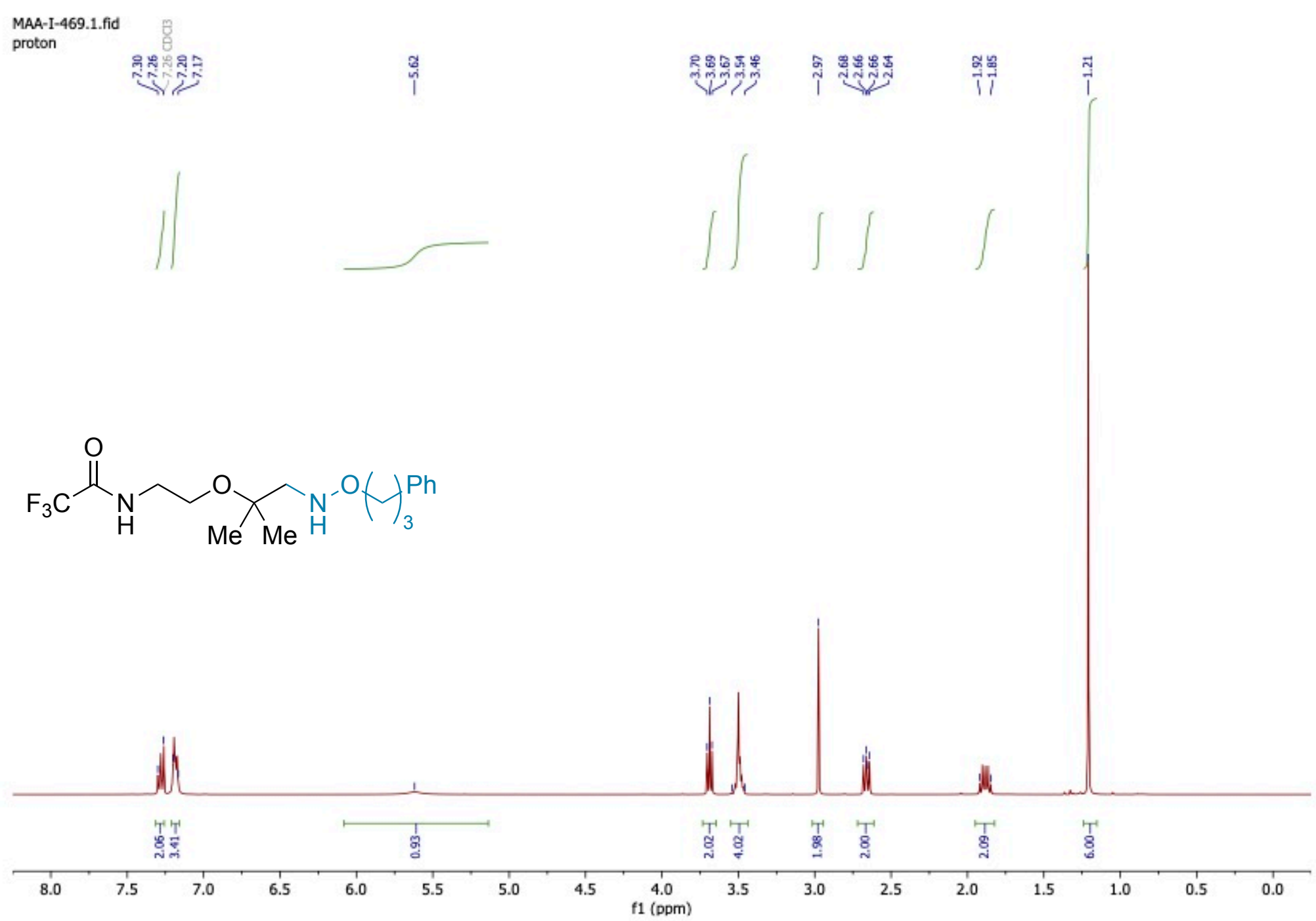

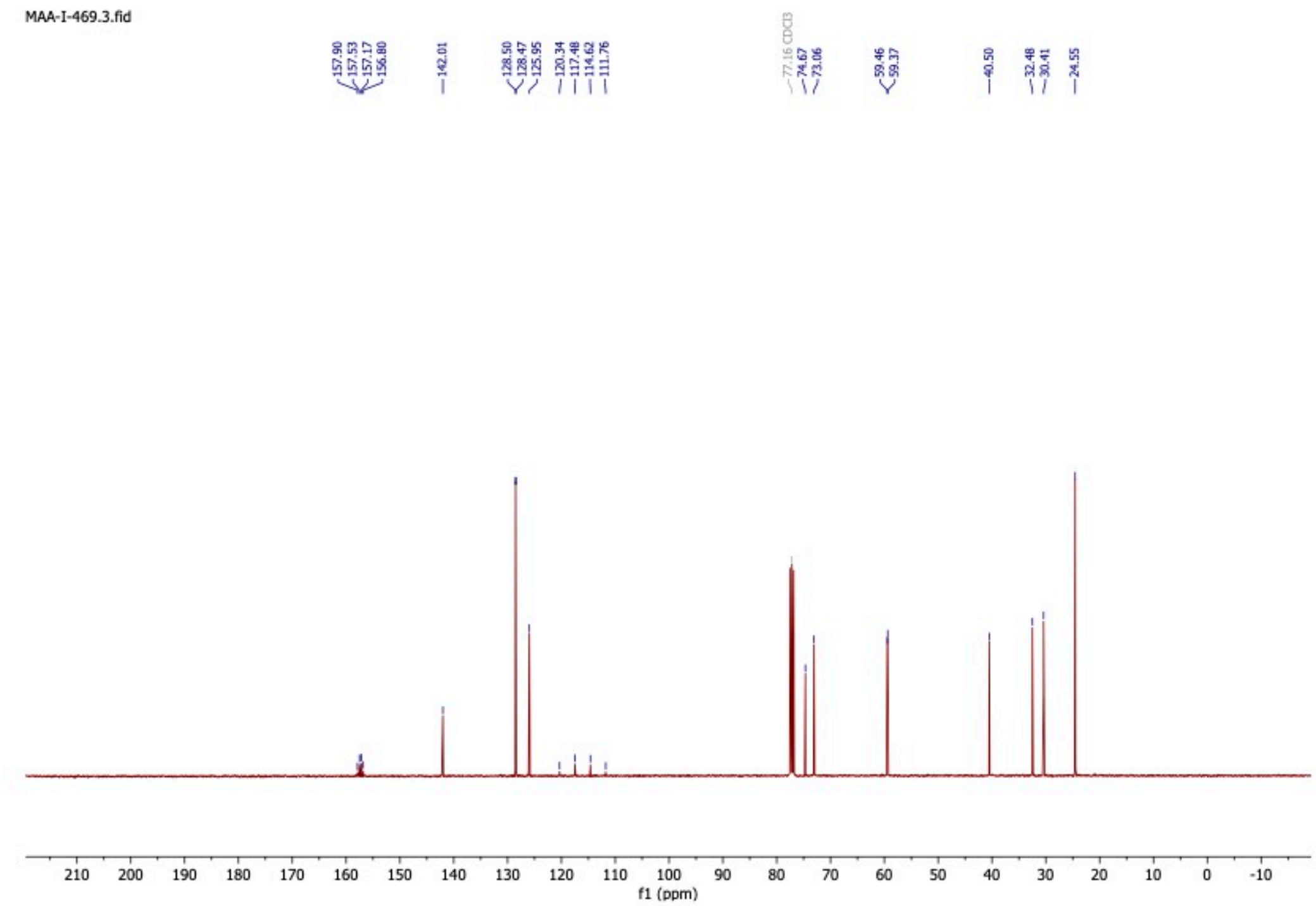
MAA-I-469.3.fid
157.90
157.53
157.17
156.80
142.01
128.50
128.47
125.95
120.34
117.48
114.62
111.76
77.16 CDCl3
74.67
73.06
59.46
59.37
40.50
32.48
30.41
24.55
f1 (ppm)

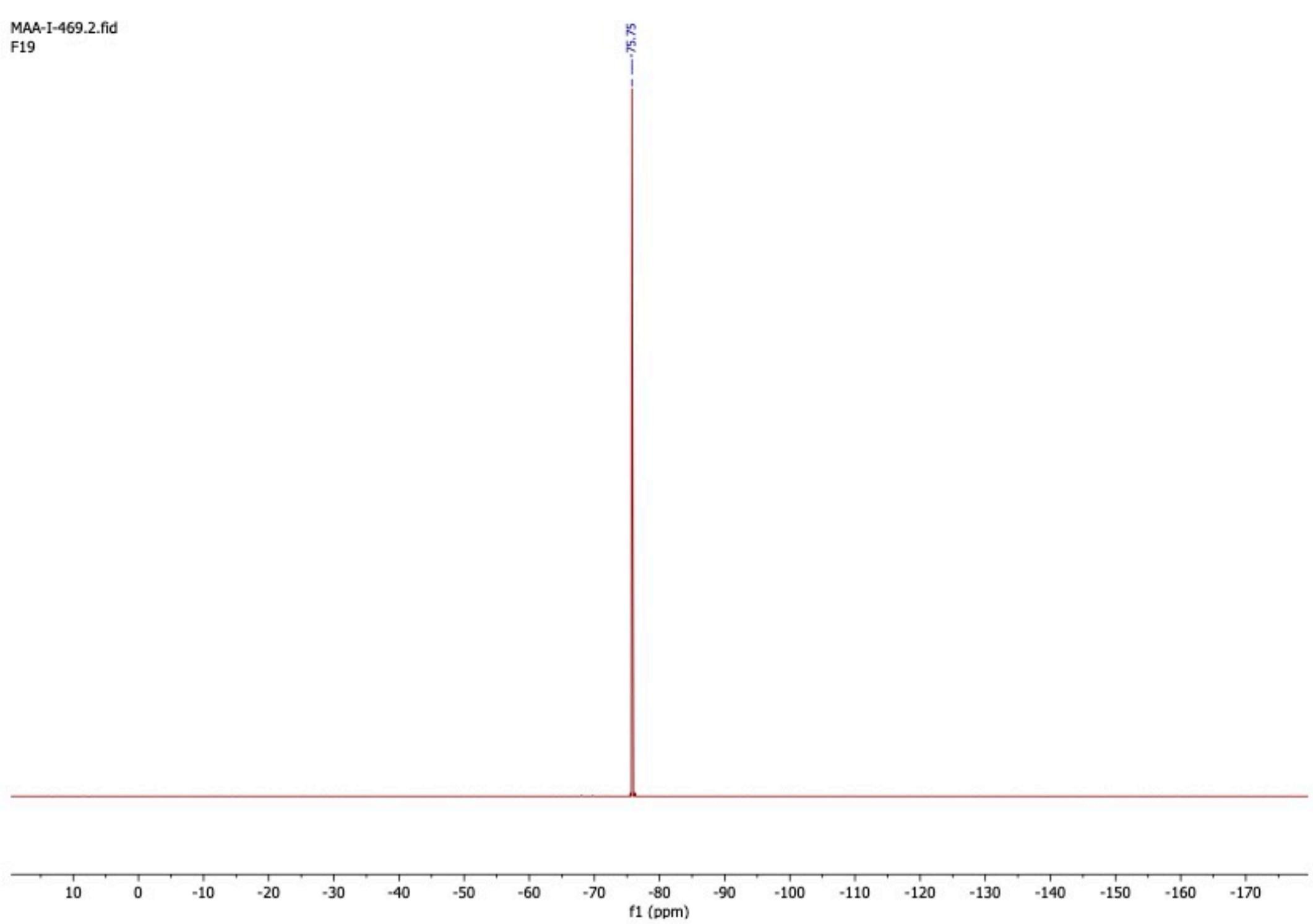
MAA-I-469.2.fid
F19
-75.75
f1 (ppm)

N-(2,5-dimethyl-6-((3-phenylpropoxy)amino)hexan-2-yl)-2,2,2-trifluoroacetamide (9)

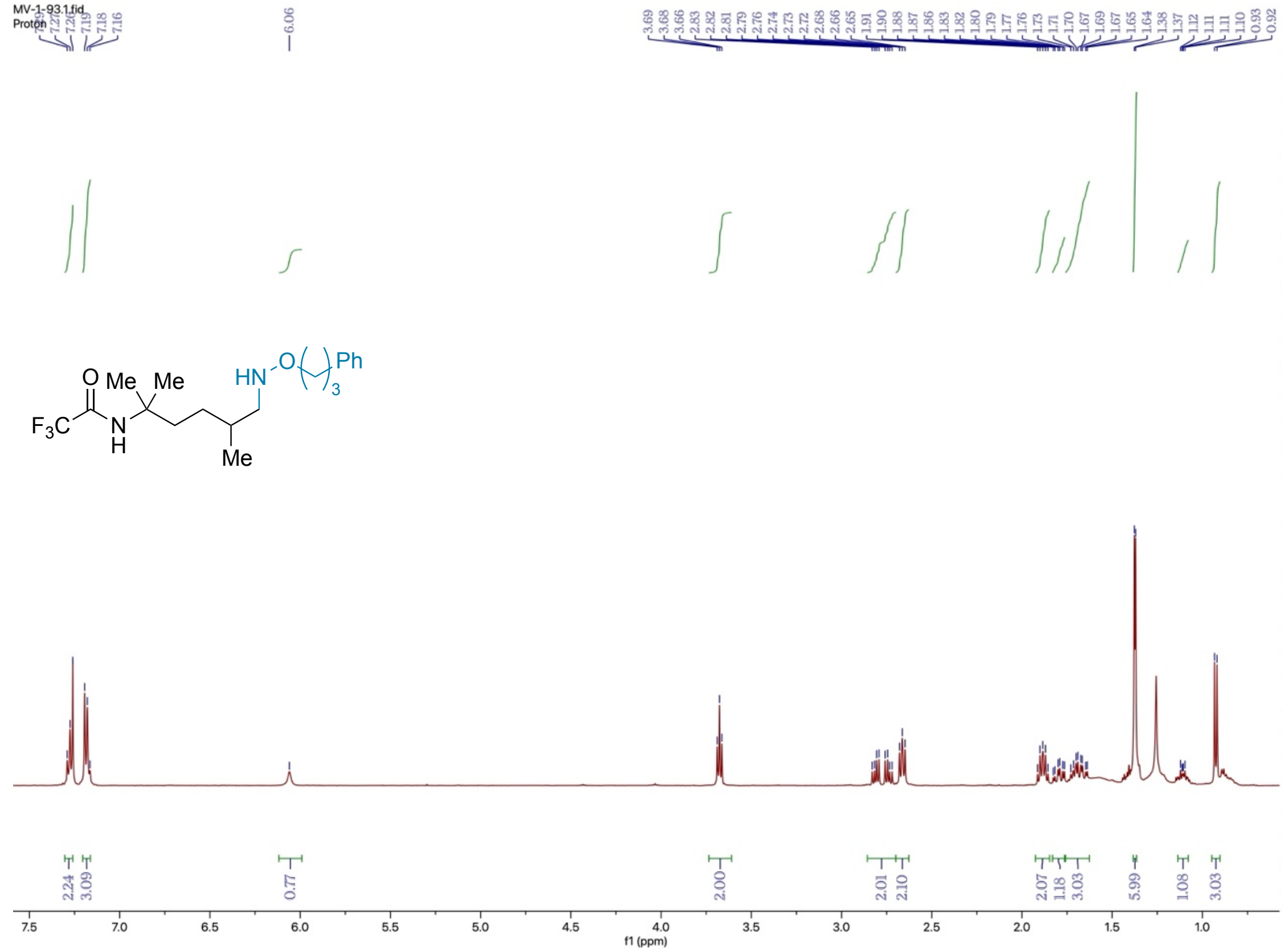

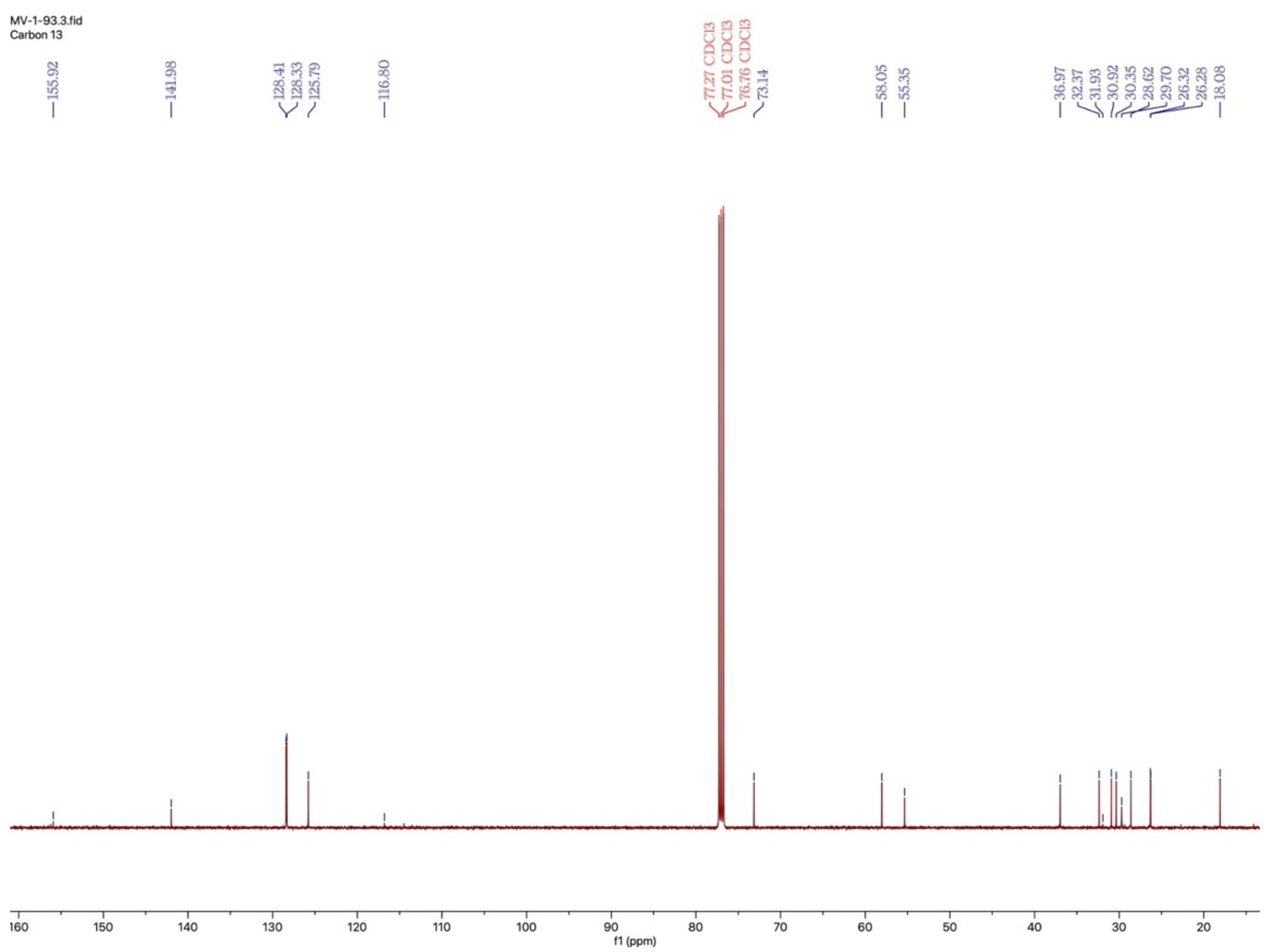

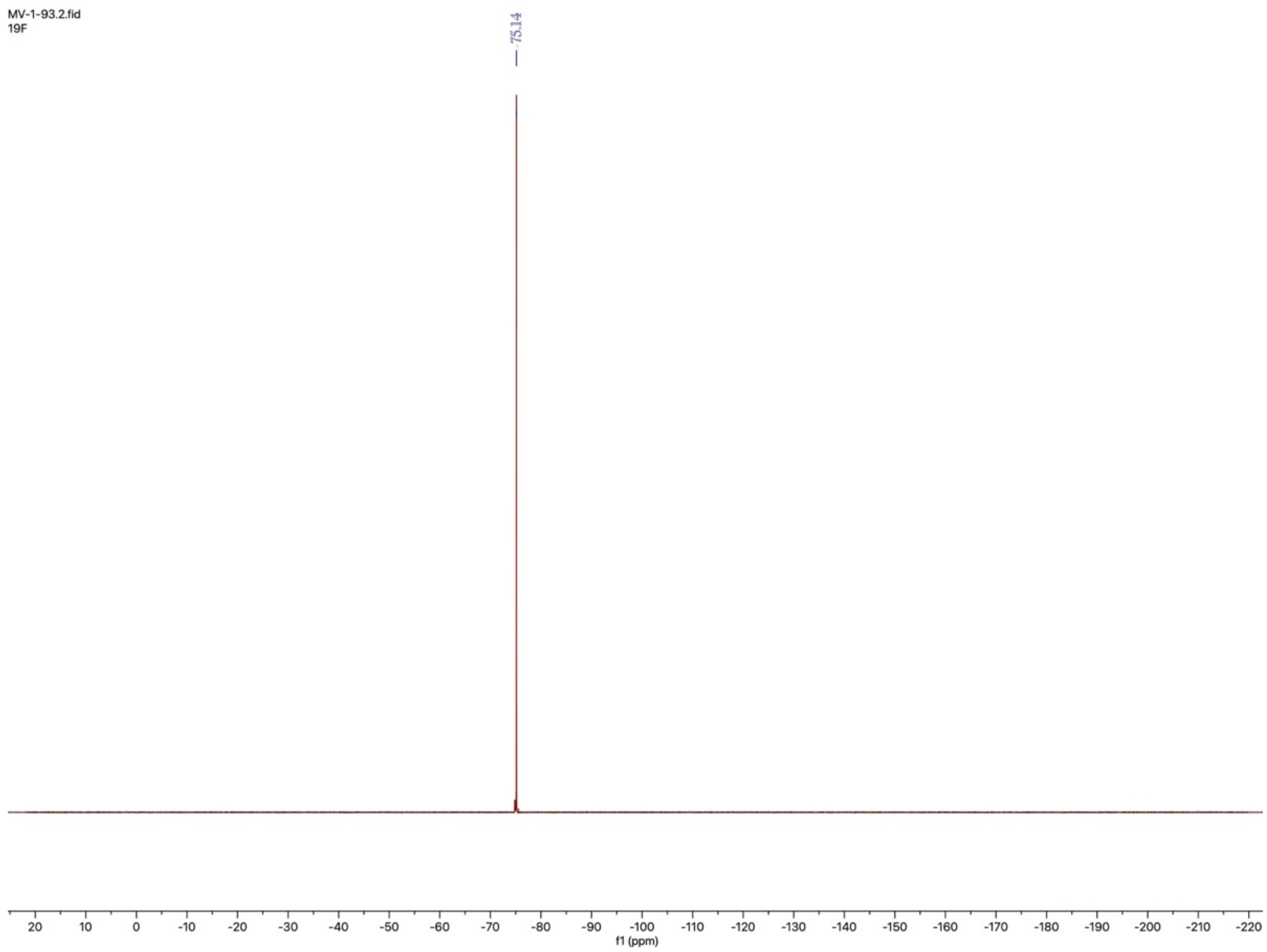

N-(4-((tert-butyldimethylsilyl)oxy)-5-((3-phenylpropoxy)amino)pentyl)-2,2,2-trifluoroacetamide (10)

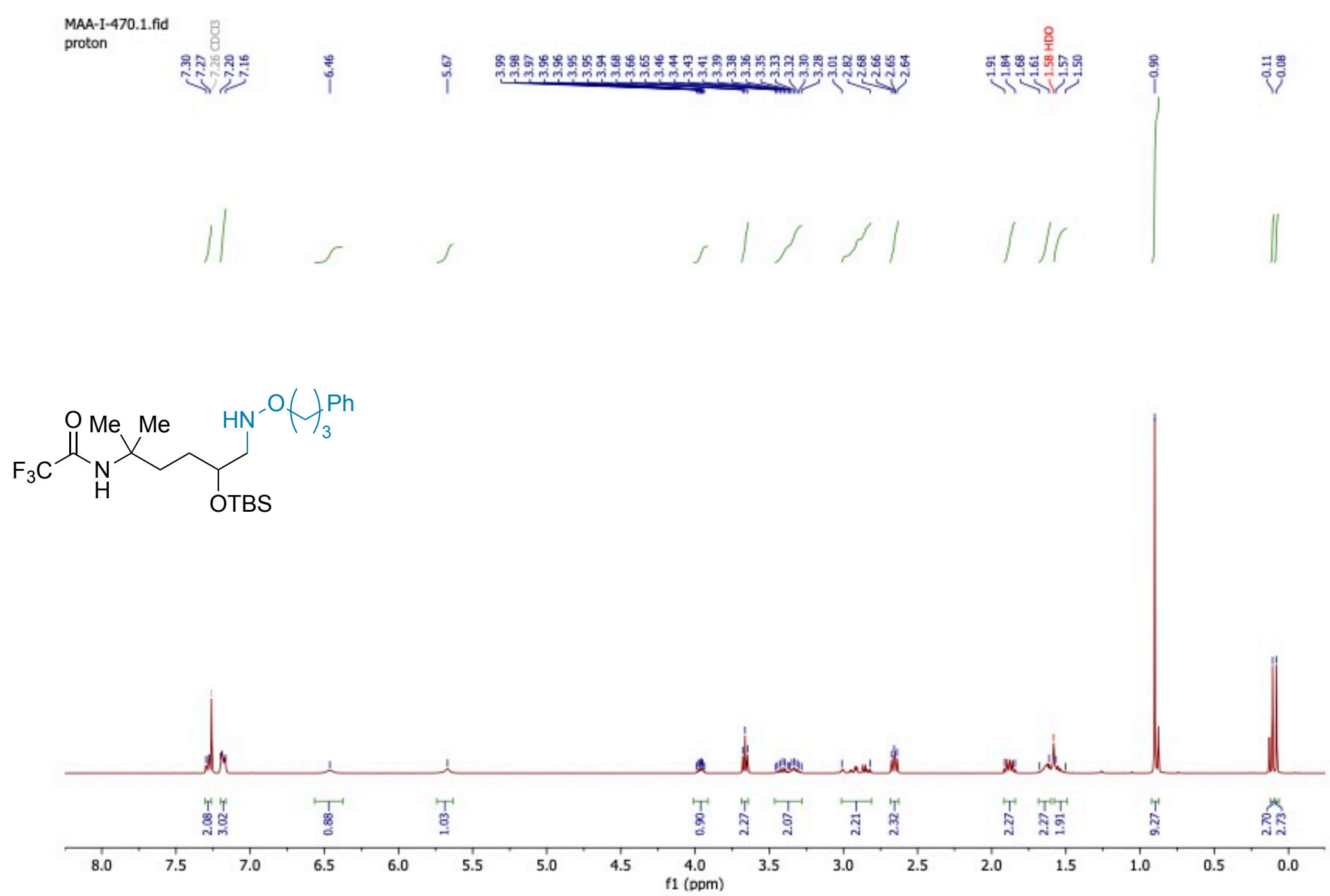

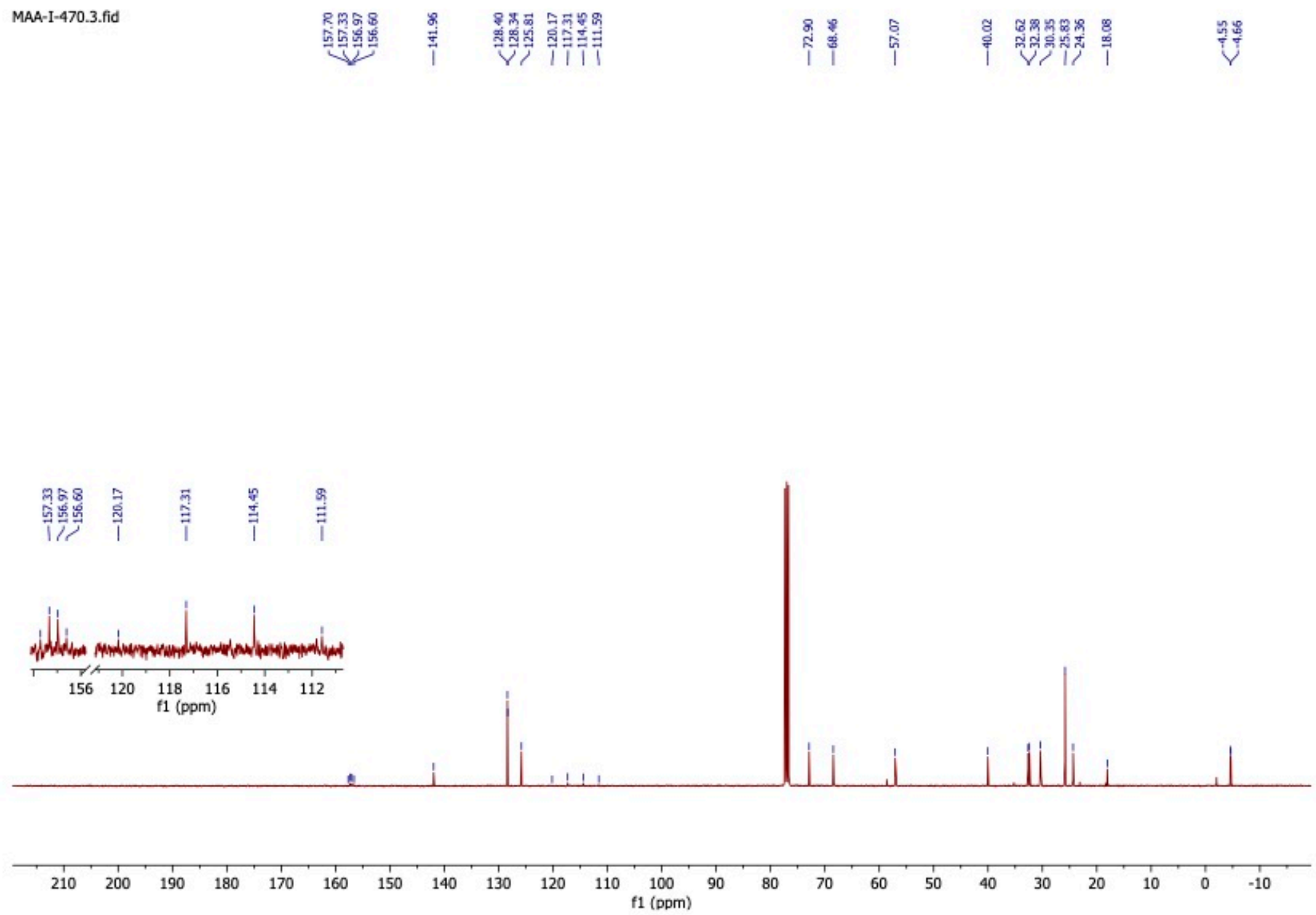
MAA-I-470.3.fid
157.70
157.33
156.97
156.60
141.96
128.40
128.34
125.81
120.17
117.31
114.45
111.59
72.90
68.46
57.07
40.02
32.62
32.38
30.35
25.83
24.36
18.08
-4.55
-4.66
f1 (ppm)

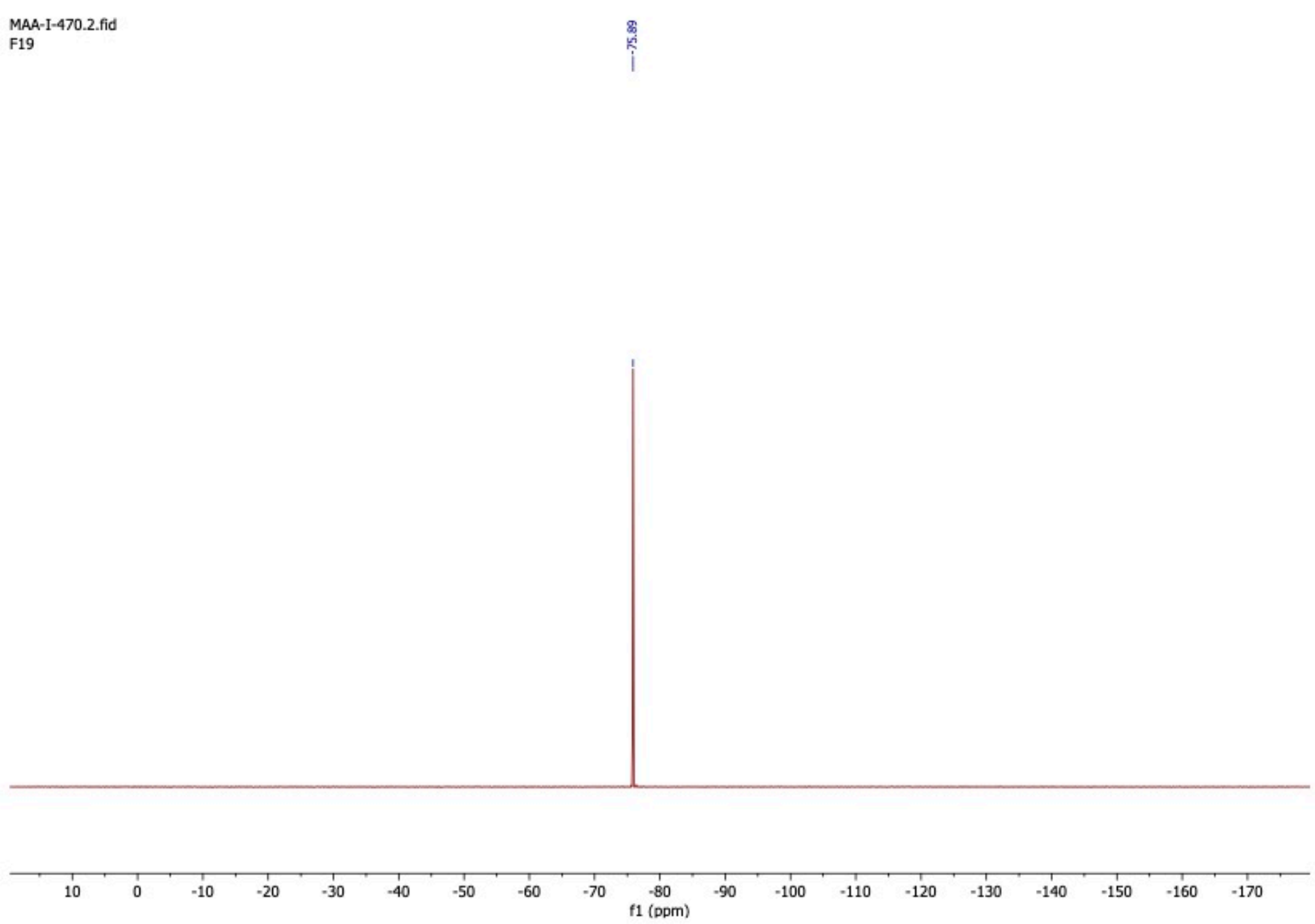
MAA-I-470.2.fid
F19
-75.89
f1 (ppm)

2,2,2-trifluoro-N-(2-(2-(((3-phenylpropoxy)amino)methyl)cyclohexyl)ethyl)acetamide (11)

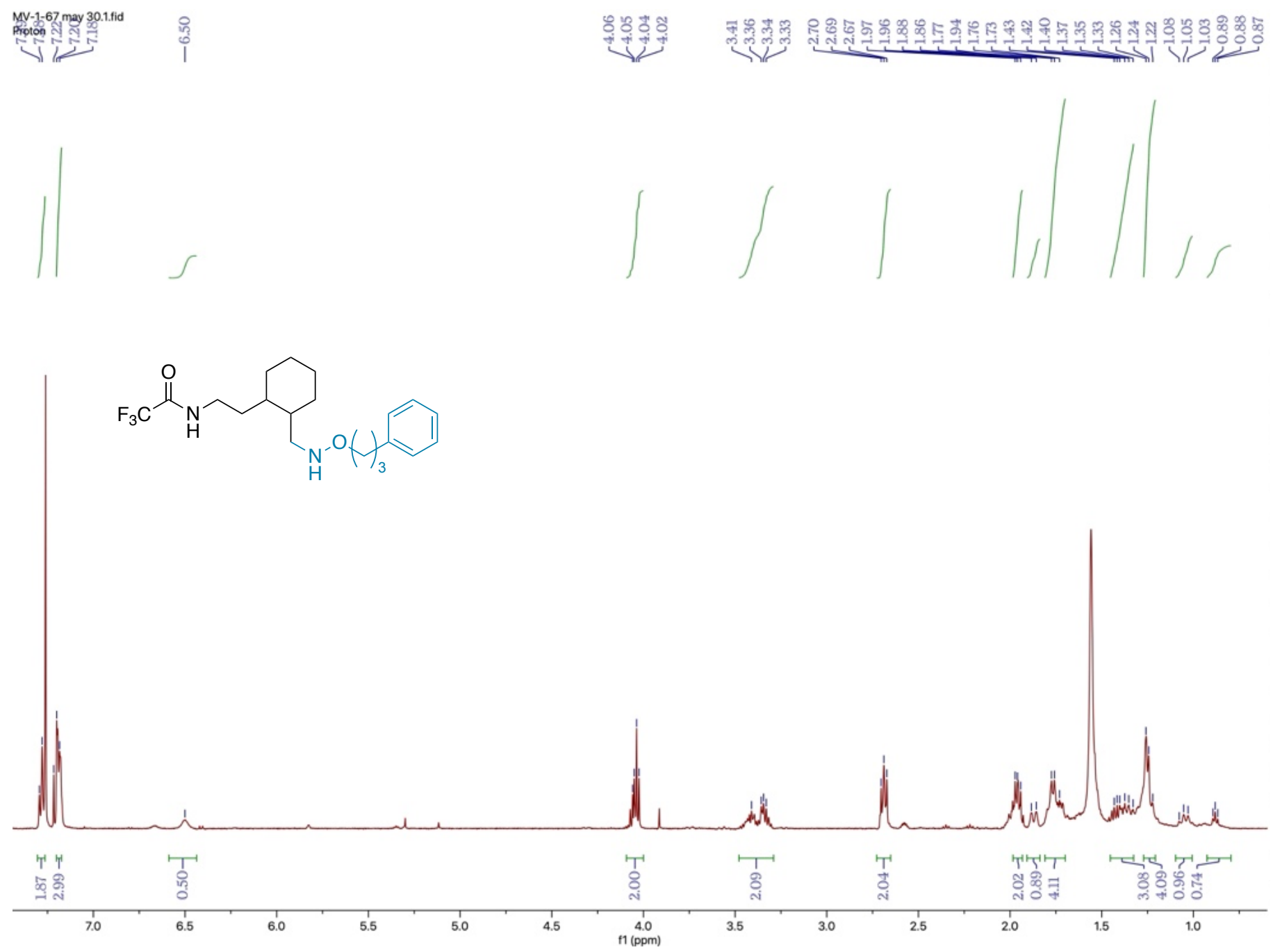

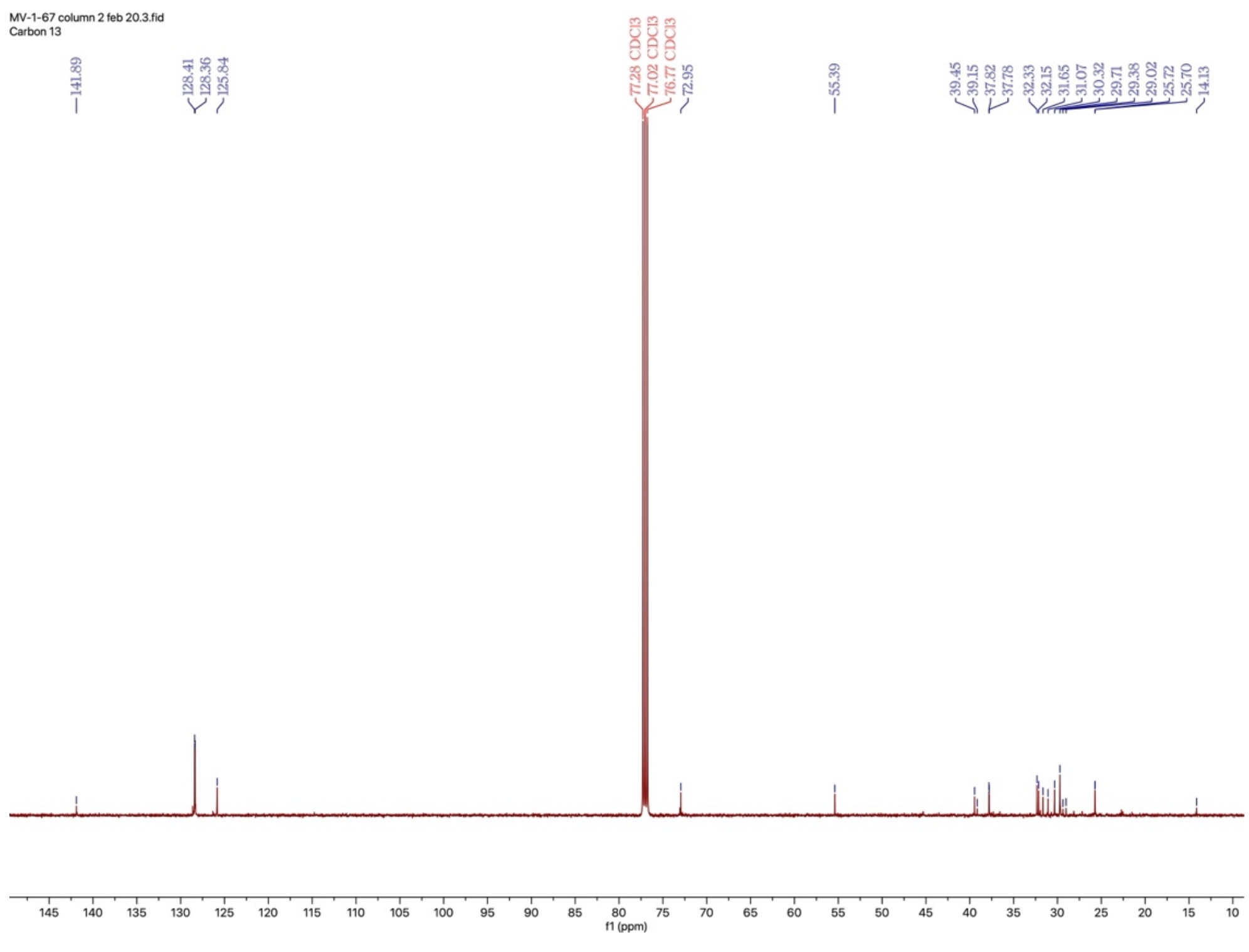

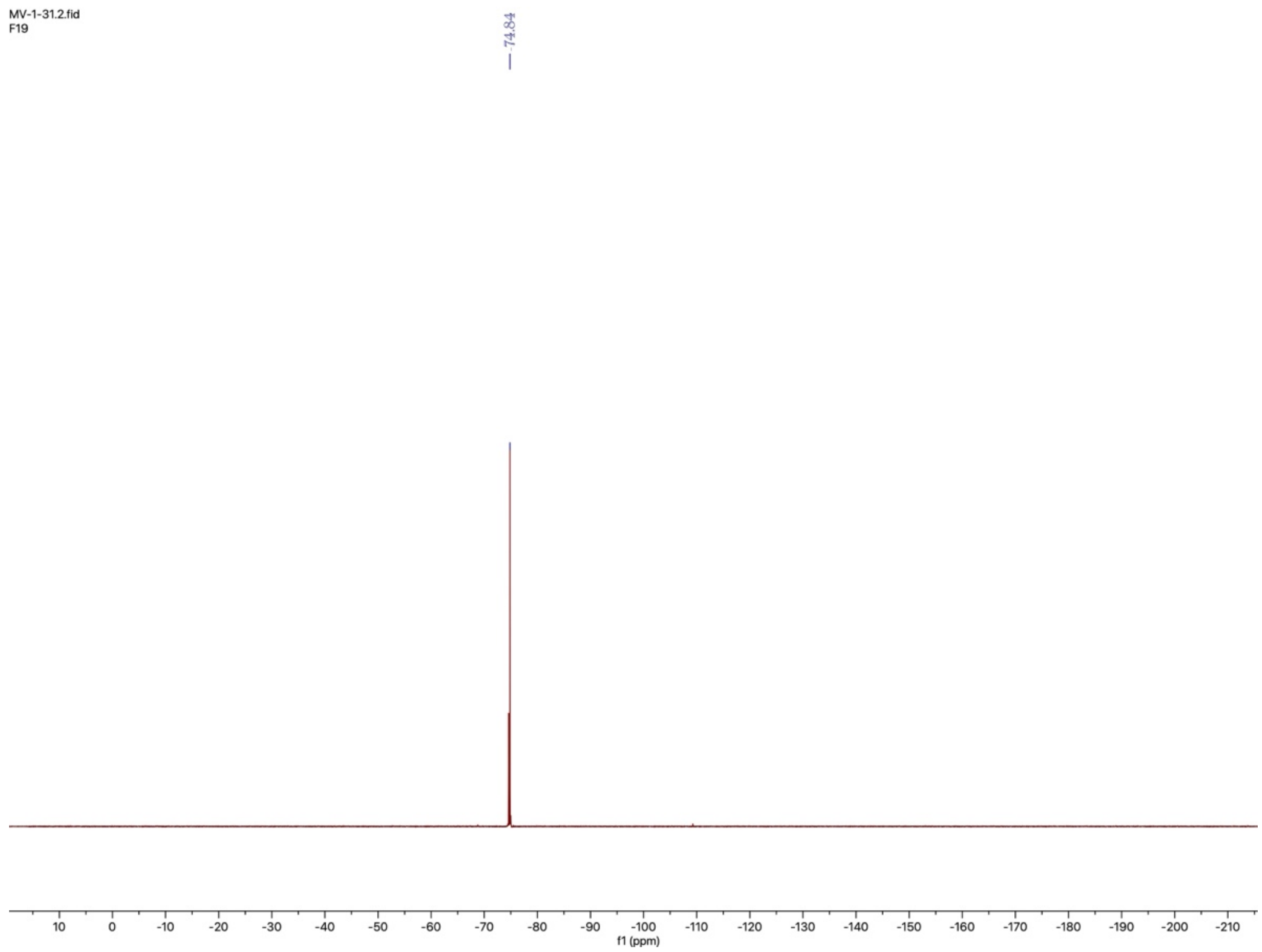

2,2,2-trifluoro-N-(6,6,6-trifluoro-4,4-dimethyl-5-((3-phenylpropoxy)amino)hexyl)acetamide (12)

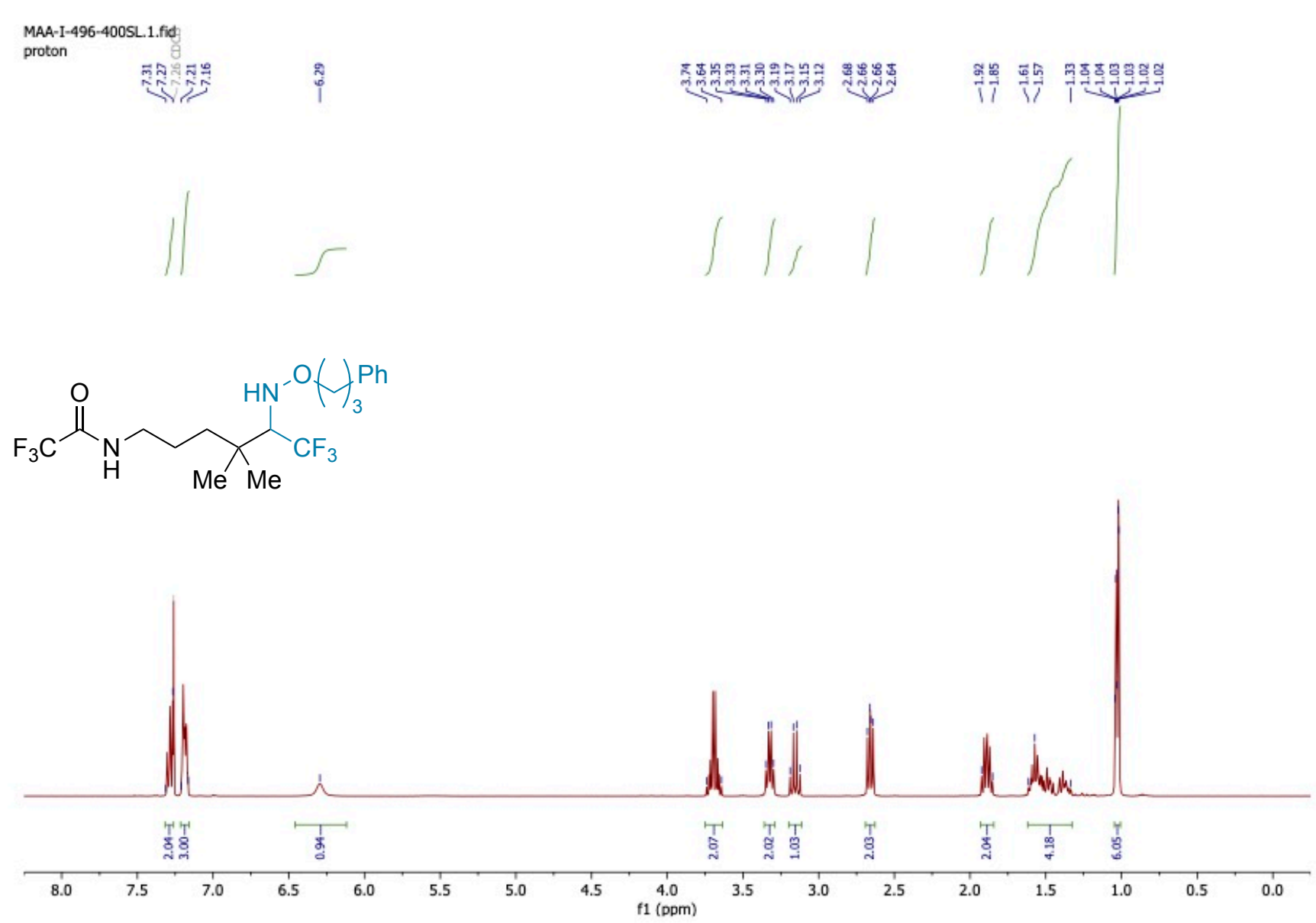

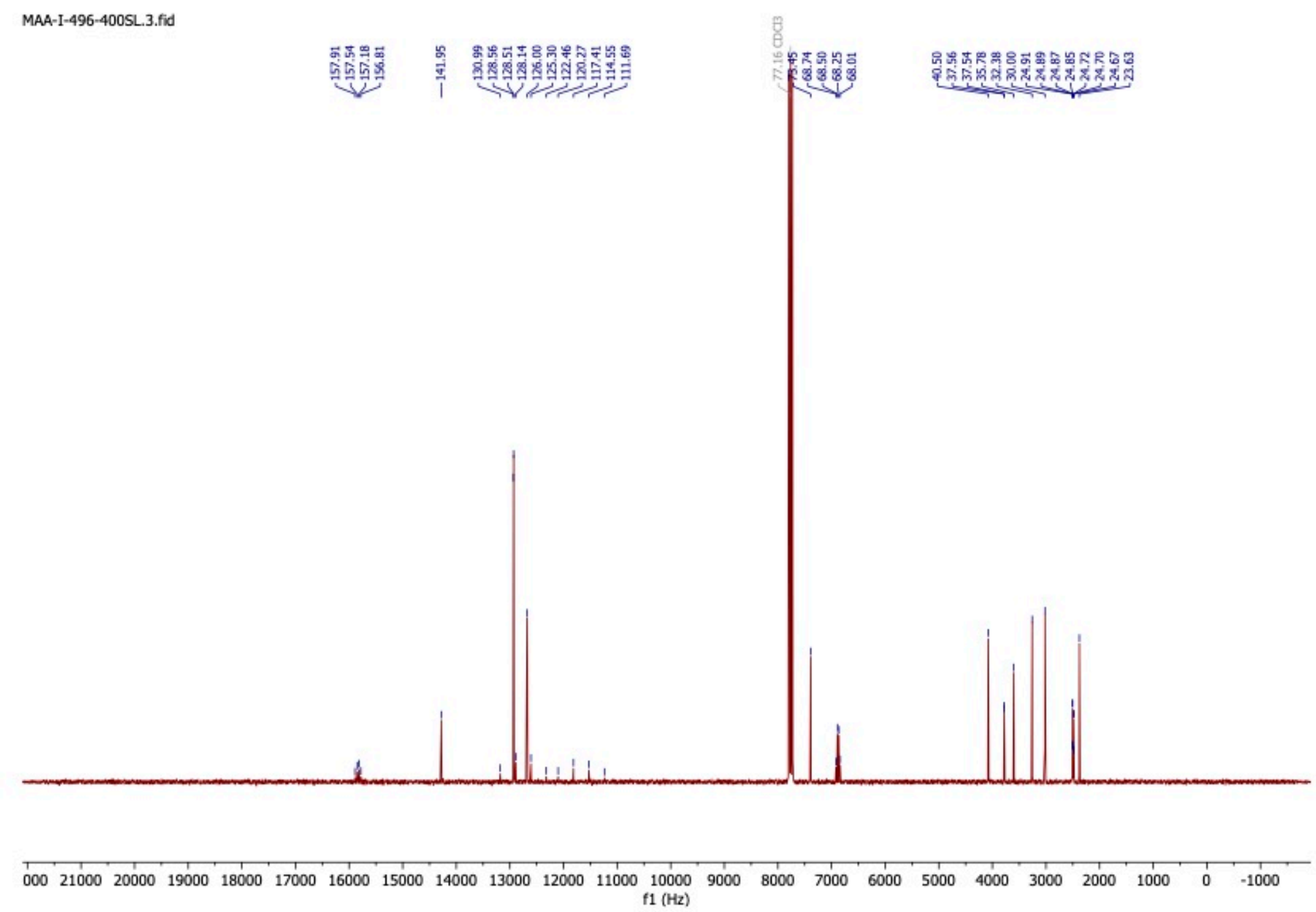
MAA-I-496-400SL.3.fid
157.91
157.54
157.18
156.81
141.95
130.99
128.56
128.51
128.14
126.00
125.30
122.46
120.27
117.41
114.55
111.69
77.16 CDCl3
73.45
68.74
68.50
68.25
68.01
40.50
37.56
37.54
35.78
32.38
30.00
24.91
24.89
24.87
24.85
24.72
24.70
24.67
23.63
f1 (Hz)

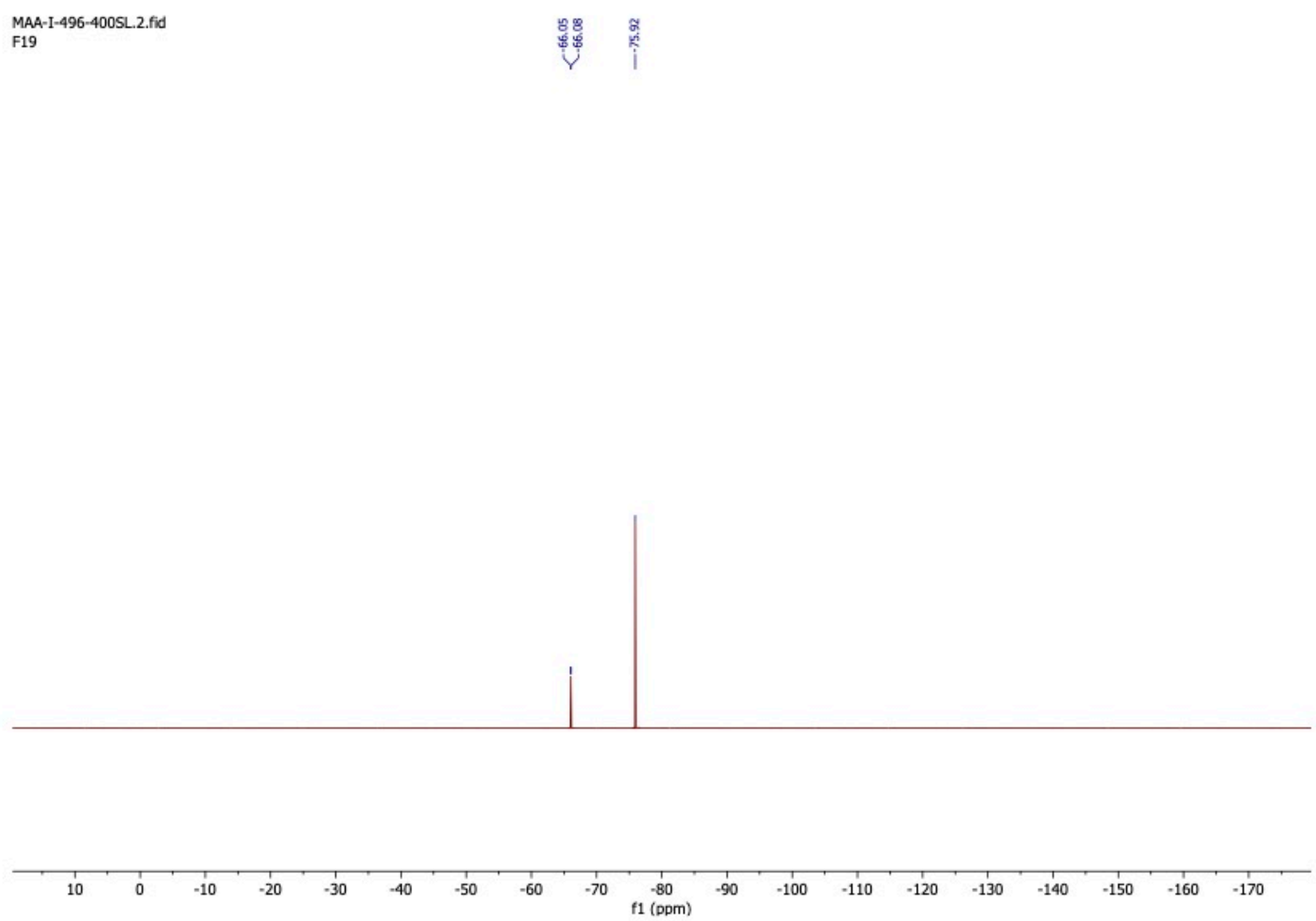
MAA-I-496-400SL.2.fid
F19
-66.05
-66.08
-75.92
f1 (ppm)

N-(4,4-dimethyl-5-(perfluorophenyl)-5-((3-phenylpropoxy)amino)pentyl)-2,2,2-trifluoroacetamide (13)

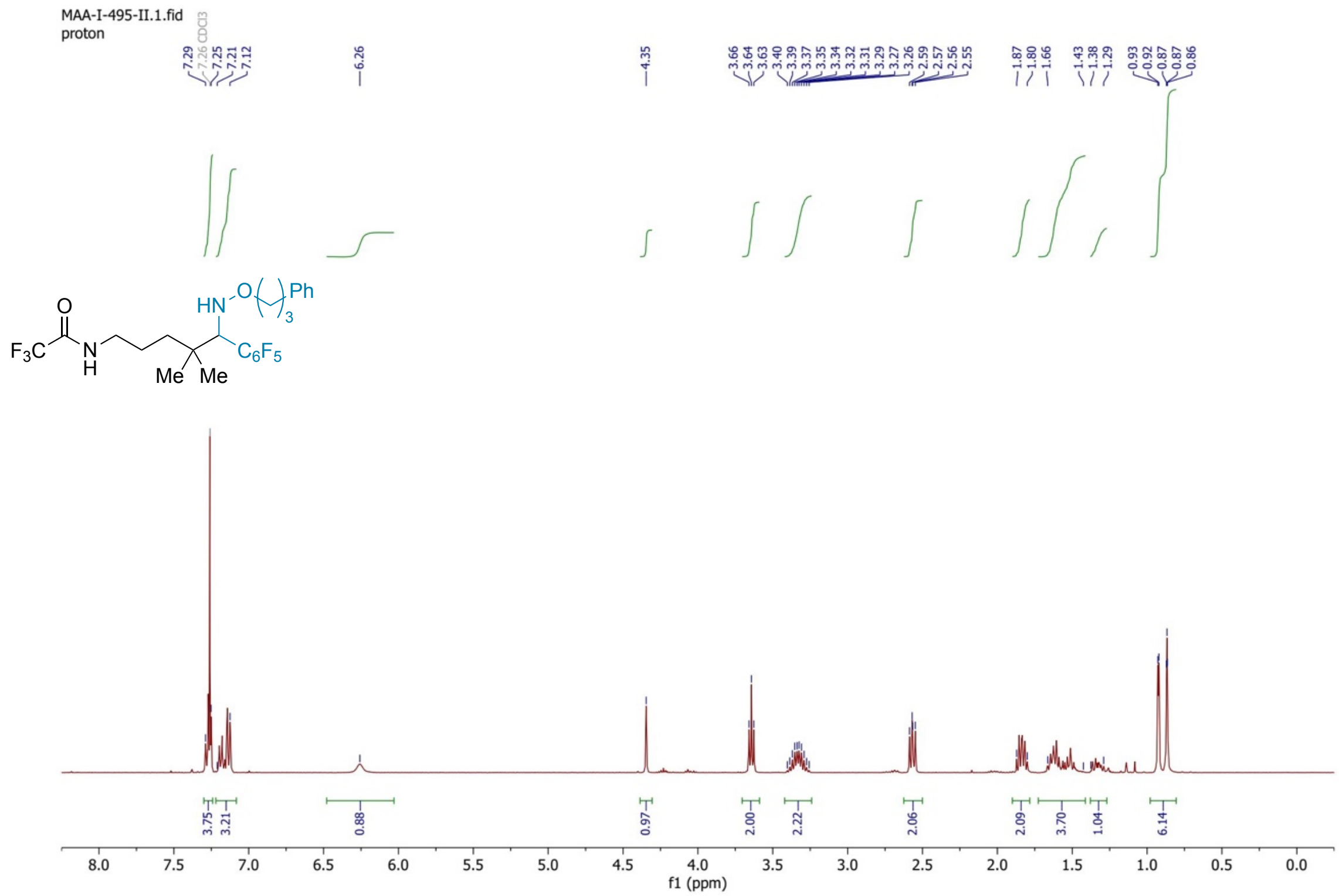

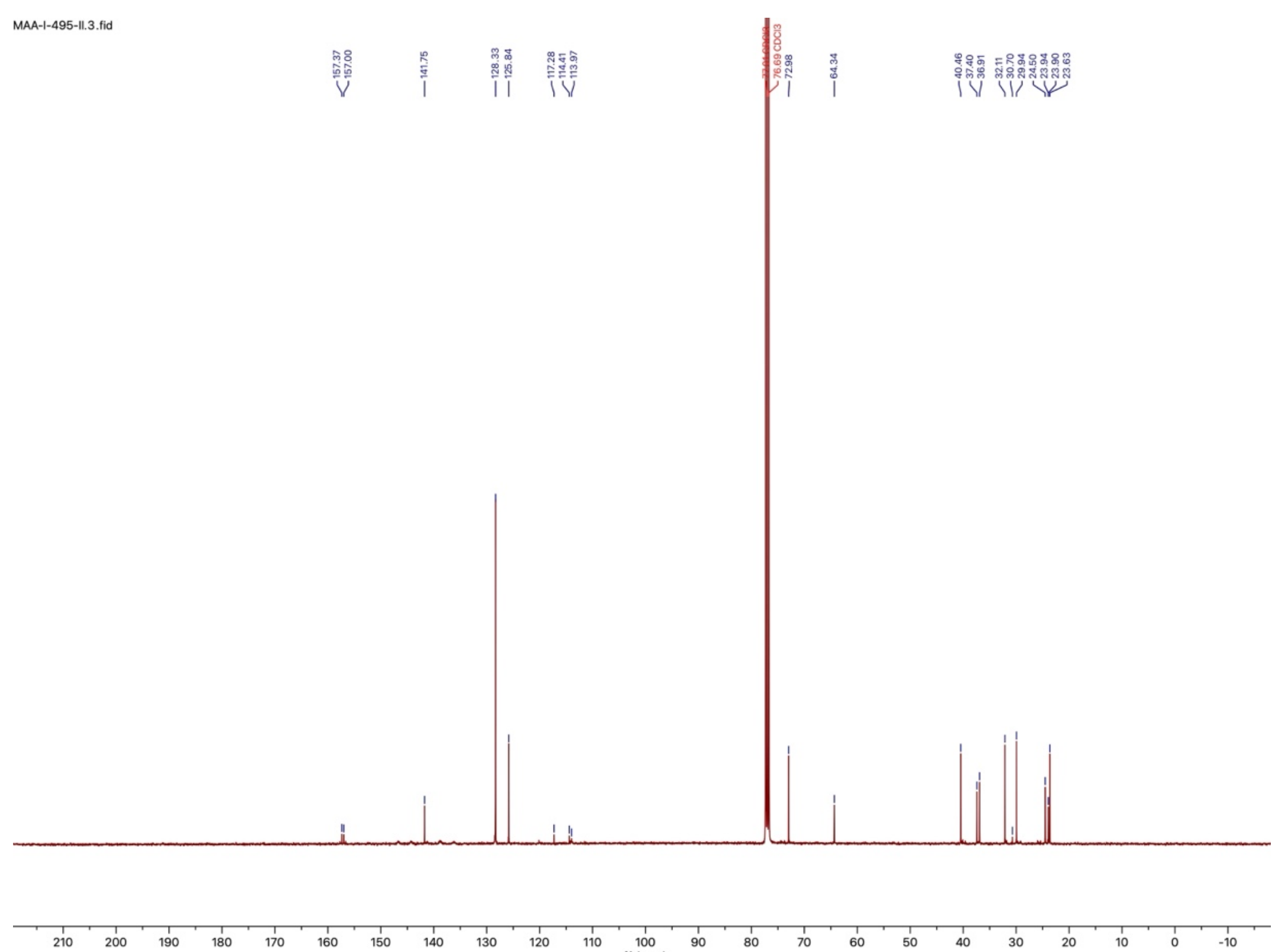

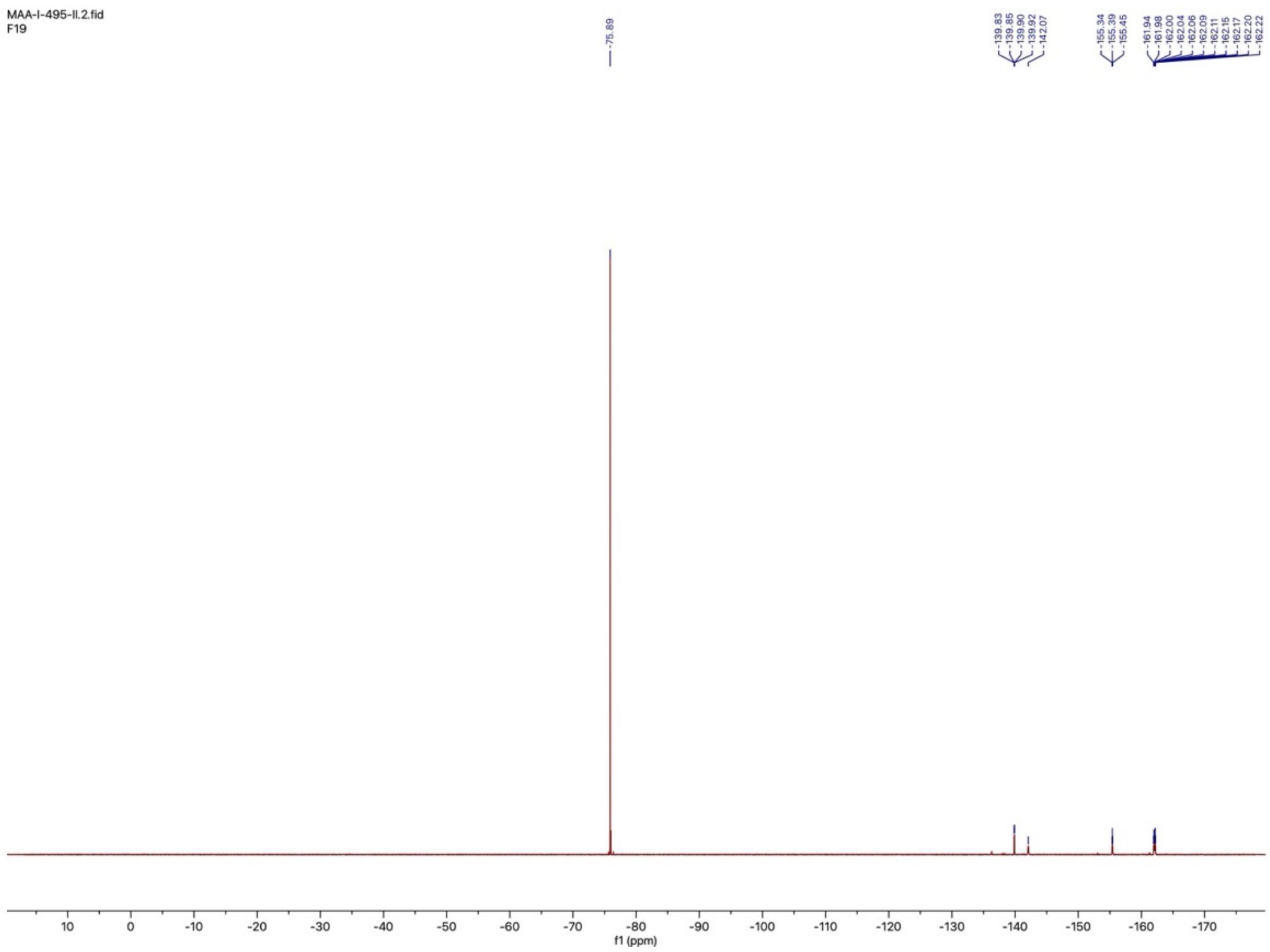

N-(4,4-dimethyl-5-((3-phenylpropoxy)amino)-5-(pyrazin-2-yl) pentyl)-2,2,2-trifluoroacetamide (14)

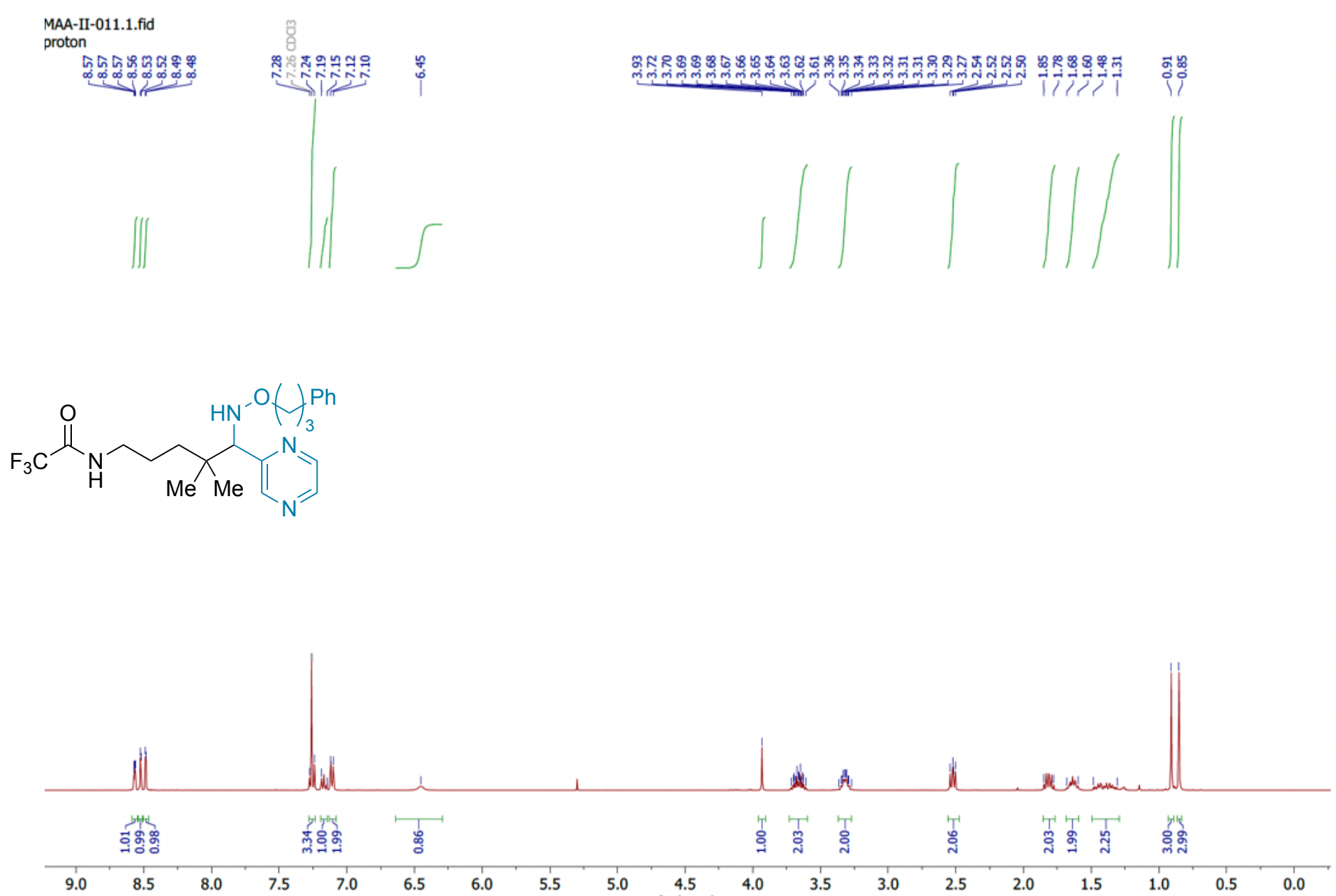

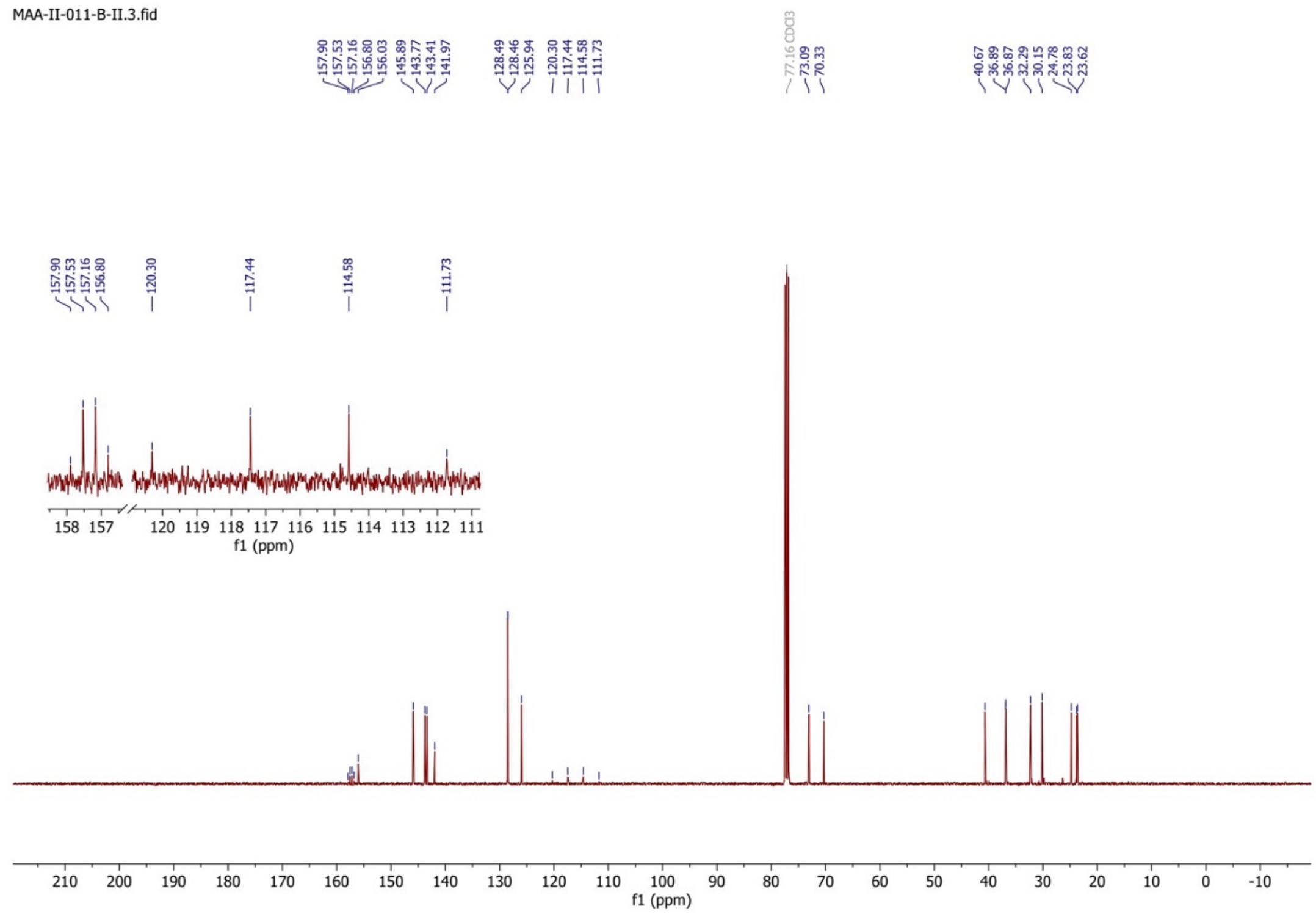
MAA-II-011-B-II.3.fid
157.90
157.53
157.16
156.80
156.03
145.89
143.77
143.41
141.97
128.49
128.46
125.94
120.30
117.44
114.58
111.73
77.16 CDCl3
73.09
70.33
40.67
36.89
36.87
32.29
30.15
24.78
23.83
23.62
f1 (ppm)

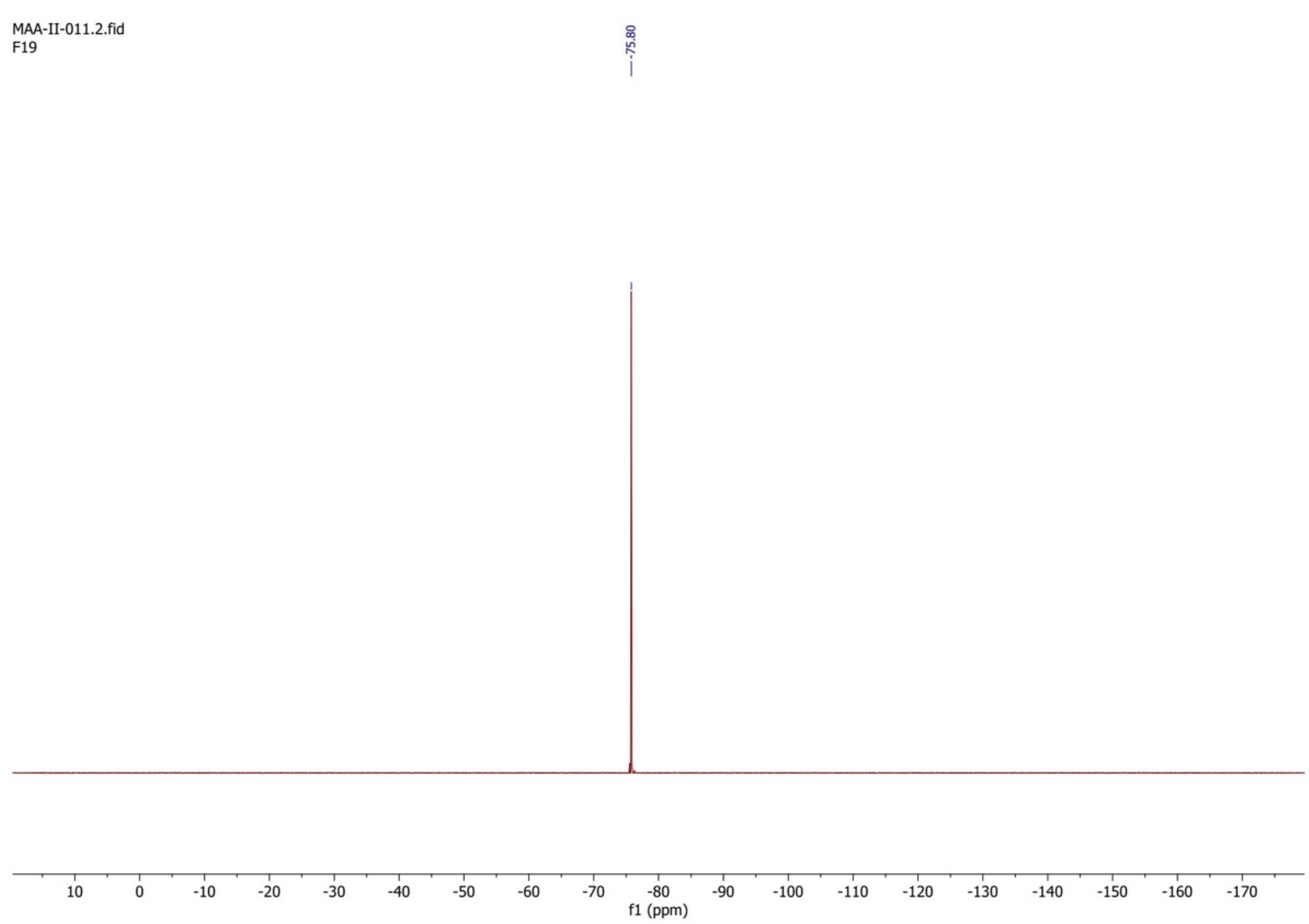
MAA-II-011.2.fid
F19
-75.80
f1 (ppm)

References

1) Seebach, D.; Imwinkelried, R.; Stucky, G. *Helv. Chim. Acta* **1987**, *70*, 448.

2) Leitch, J. A.; Rogova, T.; Duarte, F.; Dixon, D. J. *Angew. Chem. Int. Ed.* **2020**, *59* (10), 4121-4130.

3) Stache, E. E.; Ertel, A. B.; Rovis, T.; Doyle, A. G. *ACS Catal.* **2018**, *8*, 11134-11139.

4) Chu, J. C. K.; Rovis, T. *Nature* **2016**, *539*, 272-275.

5) Kvasovs, N.; Iziumchenko, V.; Palchykov, V.; Gevorgyan, V. *ACS Catal.* **2021**, *11*, 6, 3749–3754
